# Multinational evaluation of AnthropoAge as a measure of biological age in the USA, England, Mexico, Costa Rica, and China: a population-based longitudinal study

**DOI:** 10.1038/s41514-025-00232-1

**Published:** 2025-06-21

**Authors:** Carlos A. Fermín-Martínez, Daniel Ramírez-García, Neftali Eduardo Antonio-Villa, Jerónimo Perezalonso Espinosa, Diego Aguilar-Ramírez, Carmen García-Peña, Luis Miguel Gutiérrez-Robledo, Jacqueline A. Seiglie, Omar Yaxmehen Bello-Chavolla

**Affiliations:** 1 Research Division, Instituto Nacional de Geriatría, Mexico City, Mexico; 2 MD/PhD (PECEM) Program, Facultad de Medicina, Universidad Nacional Autónoma de México, Mexico City, Mexico; 3 Facultad de Medicina, Universidad Nacional Autónoma de México, Mexico City, Mexico; 4 Departamento de Endocrinología, Instituto Nacional de Cardiología Ignacio Chávez, Mexico City, Mexico; 5 Clinical Trial Service Unit & Epidemiological Studies Unit, Nuffield Department of Population Health, University of Oxford, Oxford, United Kingdom; 6 Department of Medicine, Harvard Medical School, Boston, MA USA; 7 Diabetes Unit, Massachusetts General Hospital, Boston, MA USA

**Keywords:** Biomarkers, Epidemiology

## Abstract

We validated AnthropoAge, a biological age (BA) metric, for prediction of mortality and age-related outcomes using harmonized data from the US, England, Mexico, Costa Rica, and China. We estimated AnthropoAge and AnthropoAgeAccel as proxies of BA and age acceleration using body mass index and waist-to-height ratio. We compared mortality prediction of AnthropoAge vs. chronological age (CA) using Cox models and assessed its association with age-related outcomes with generalized estimating equations. Among 57,080 participants aged 50–94 years, AnthropoAgeAccel (c-statistic 0.806) improved mortality prediction of CA (0.803) and identified distinct aging trends for each country. Accelerated aging (AnthropoAgeAccel>0) increased mortality risk by ~37% independently of age and covariates, and predicted health deterioration, new deficits in activities of daily living, and age-related diseases. AnthropoAge is a robust BA metric with potential applications in identifying functional deficits, health decline, and mortality risk. However, it requires further validation and potential recalibration for broader applicability in underrepresented populations like Latin America.

## Introduction

Aging is a highly heterogeneous and complex process that causes a progressive deterioration in multiple body systems, ultimately leading to disease, disability, and death^1,2^. Although chronological age (CA) is a key risk factor for these outcomes, it is often unable to capture the variability inherent to the aging process, and numerous efforts have been made to quantify this heterogeneity. Biological age (BA) metrics, often called aging clocks^3^, are measures expressed in units of years that combine numerous aging biomarkers and have been proposed as an alternative to CA as they capture changes in biological systems more precisely, better reflecting the true status of health and biological decline of an individual^4,5^. In the context of epigenetic aging clocks, first generation clocks are trained to predict CA from individual biomarkers, while second generation clocks are trained to predict age-related outcomes, such as mortality risk and comorbidities^6^; finally, third generation clocks are able to model the pace of aging by capturing longitudinal changes in aging biomarkers^7,8^. Recent evidence suggests that BA clocks capture unique aging processes that reflect trajectories of functional, cognitive, and physical decline and may be superior to CA alone^9–11^. Despite this evidence, the utility of BA metrics remains controversial, as many other frameworks closely related to biological aging have been widely implemented in clinical geriatric assessments for years^12^. Nonetheless, the development of aging clocks continues to be relevant, currently, there is a lack of reproducible, precise, and simple methods that can serve as surrogates for the aging rate of an individual, which would allow to study population aging patterns, evaluate interventions aimed at healthy aging, and even uncover new biological mechanisms that explain the heterogeneity of this process^13,14^. Most BA measures have been developed using blood methylation data, while more recent approaches have incorporated a wide array of omics to integrate data of different system levels into the assessment of aging^15^. However, due to the complexity of implementing omics-based measures in epidemiological studies, the application of BA to measure aging at a population-level remains limited^16^.

Our team recently developed AnthropoAge, a non-invasive surrogate of BA using anthropometry to capture body composition aging, and mortality risk^17^. This metric represents an accessible and potentially useful aging biomarker to assess population aging^5,16^. Although AnthropoAge was developed using nationally representative data of participants with diverse ethnical and racial backgrounds, this may not fully capture the wide range of global diversity since all participants from the original study were from the U.S. Whether our metric yields an adequate performance in diverse populations across different geographic contexts, and whether repeated assessments of AnthropoAge may be useful to detect functional and health outcomes has not been examined. In this population-based, multinational, longitudinal study, we leveraged harmonized data from the Gateway to Global Aging (G2A) from the United States (US Health and Retirement Study, HRS), England (English Longitudinal Study of Ageing, ELSA), Mexico (Mexican Health and Aging Study, MHAS), Costa Rica (Costa Rican Longevity and Healthy Aging Study, CRELES), and China (China Health and Retirement Longitudinal Study, CHARLS) with the main objective of validating AnthropoAge as a BA metric predictive of all-cause mortality, health deterioration and functional deficits in diverse populations, and using it to characterize trends of population aging.

## Results

### Study population

**Fig. 1:**
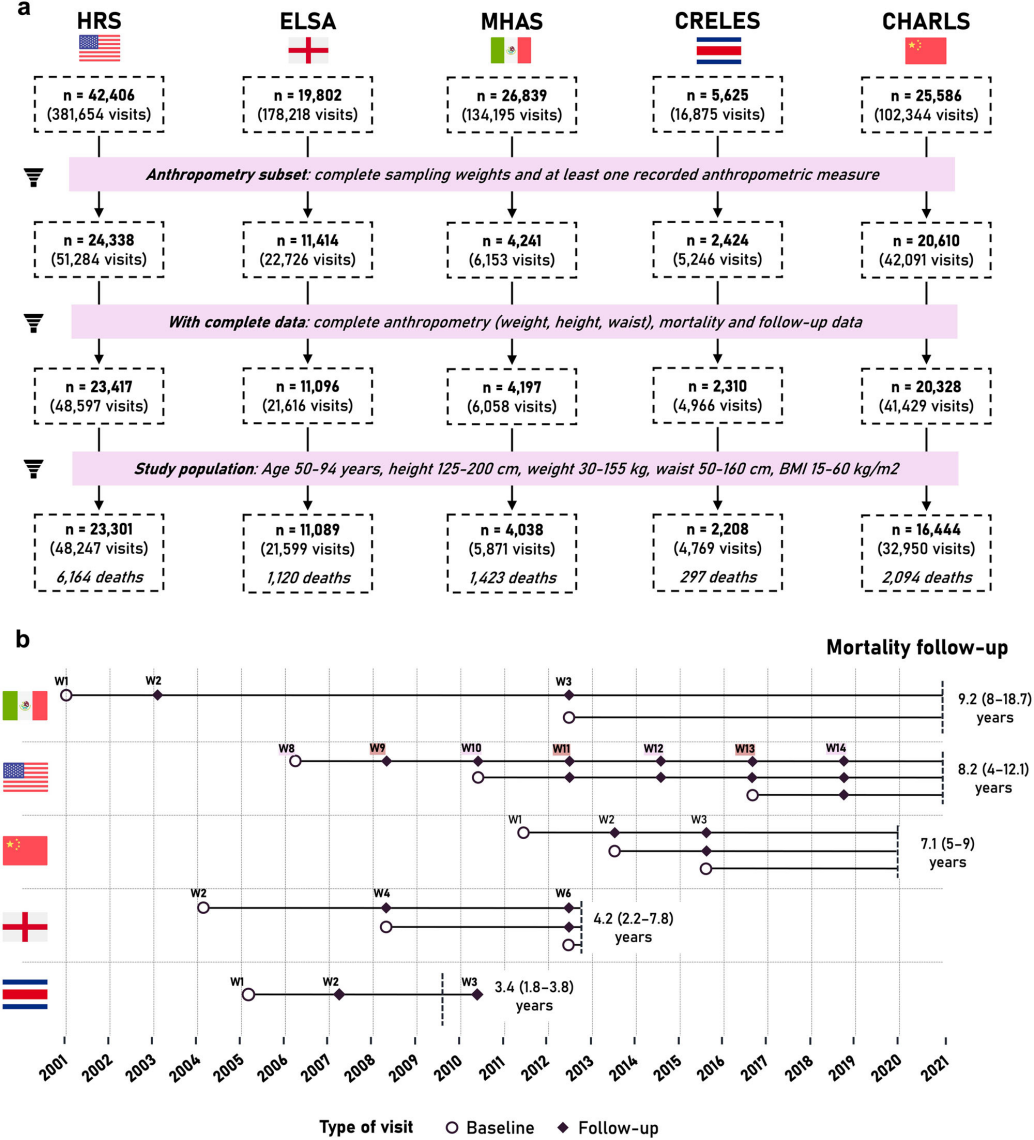
Study participant selection and follow-up timeline. **a** Flowchart depicting selection for each survey in the harmonized Gateway to Global Aging (G2A) data. Participants with at least one recorded anthropometric measure were included (anthropometry subset). We excluded those with missing data on mortality, follow-up, or anthropometry (complete data subset). Further exclusions were made for participants younger than 50 or older than 94 years and for those with anthropometric measurements outside specified ranges to enhance comparability between surveys and ensure stable estimations. **b** Overview of follow-up periods for included participants. All surveys, except CRELES, included multiple cohorts. Circles indicate the first visit for each cohort, while diamonds represent subsequent visits. Numbers above each timepoint represent the survey wave. In HRS, anthropometric measurements were obtained for half of the respondents every two waves (highlighted in the same color). The final study sample comprised 57,080 participants, with a total of 11,098 deaths over a joint median follow-up of 7.5 years (IQR: 3.9–9.1 years).

Among a total of 120,258 participants across all five G2A studies, 63,027 were selected for anthropometry assessment, and 61,348 had complete anthropometric (weight, height, and waist circumference) and mortality data. Of them, 57,080 (study population) were 50–94 years old and had anthropometric measurements within specified ranges, comprising a total of 113,436 longitudinal evaluations. Detailed flowcharts of participant selection and follow-up are presented in Fig. 1 and Supplementary Figs. 1–5. Participants had a median age of 61 years (IQR 55–70 years), most of them were female (55%), and predominantly Non-Hispanic White (45%), followed by Asian (30%) and Hispanic/Latino participants (17%). Participants in CRELES were older (75 [69–82] years), while participants in CHARLS were younger (59 [53–66] years). Median body mass index (BMI) was 26.6 kg/m^2^ (IQR 23.3–30.6), and median waist-to-height ratio (WHtR) was 0.58 (0.53–0.64), with CHARLS participants being leaner (BMI 23.3 [20.9–25.9] kg/m^2^, WHtR 0.54 [0.50–0.59]). Most participants had an education of primary or less (50%), were never smokers (50%), and did not consume alcohol (47%); however, these percentages varied across countries. A complete description of baseline characteristics is outlined in Supplementary Table 1. Differences between subsets of participants and missing data analyses for each country are shown in Supplementary Tables 2–6 and Supplementary Figs. 5–10, respectively.

### Biological aging and anthropometric metrics

We previously developed AnthropoAge as a proxy of BA by using CA and anthropometry to predict sex- and ethnicity-specific mortality risk. Here, we employed the simplified version of AnthropoAge, which uses CA in years, BMI, and WHtR^17^. At baseline, median AnthropoAge was 61 years (IQR 55–70) for the overall population, 63 (56–72) years for HRS, 62 (55–70) years for ELSA, 63 (56–71) years for MHAS, 76 (69–83) years for CRELES, and 57 (51–64) years for CHARLS participants. We derived a measure of age acceleration, called AnthropoAgeAccel, by adjusting AnthropoAge to remove the influence of CA (See Methods). Having AnthropoAgeAccel values > 0 (indicative of accelerated aging) implies that mortality risk predicted by anthropometric measures exceeds the risk expected based on CA alone. At baseline, the median AnthropoAgeAccel was -0.4 (-2.1, 1.4) years, and 43% of participants had accelerated aging, which was associated with lower education level, lower weight and height, higher waist circumference and WHtR, a higher frequency of smoking, and higher proportions of ADL/IADL deficits and self-reported comorbidities (Supplementary Table 7). To evaluate whether AnthropoAge and AnthropoAgeAccel more effectively capture mortality risk compared to other anthropometry-based markers, we used Body Roundness Index (BRI)^18^, Weight-Adjusted Waist Index (WWI)^19^, and A Body Shape Index (ABSI)^20^ as benchmarks for comparison. AnthropoAge and AnthropoAgeAccel can be estimated using the AnthropoAge R package (https://github.com/oyaxbell/AnthropoAgeR) and a Shiny App (https://bellolab.shinyapps.io/anthropoage/).

### Performance of AnthropoAgeAccel for all-cause mortality

**Fig. 2:**
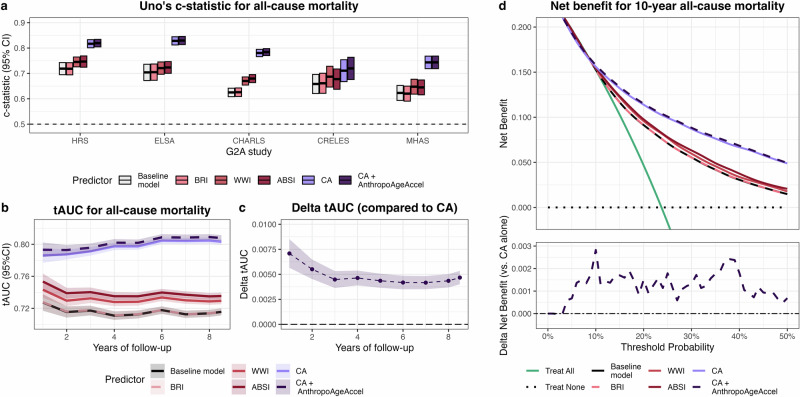
Comparison of predictive performance for all-cause mortality across models. **a** Predictive performance stratified by G2A survey. The baseline model included sex, race/ethnicity, education level, smoking, alcohol consumption, and self-report of hypertension, diabetes, myocardial infarction, stroke, cancer, chronic lung disease, and arthritis. Additional models incorporated chronological age (CA) alone, CA plus AnthropoAgeAccel, or anthropometric indices: body roundness index (BRI), weight-adjusted waist index (WWI) and a body shape index (ABSI). Predictive performance was assessed using Uno’s c-statistic from Cox models. **b** Time-dependent areas under the receiving operating characteristic curve (tAUC) over eight years of follow-up for the same models. **c** Delta tAUC, calculated as the difference in tAUC between the CA plus AnthropoAgeAccel model versus CA alone model, where values greater than zero indicate superior performance of the former. **d** Decision curve analysis illustrating the net benefit of each model in predicting mortality risk. In the upper panel, curves closer to the top right represent models with better discrimination and greater net benefit. The lower panel shows the delta net benefit compared to CA alone. The combination of CA plus AnthropoAgeAccel provides a higher net benefit relative to CA alone and other anthropometric biomarkers, supporting its clinical utility. Analyses were conducted among participants with complete covariate data (*n* = 51,527).

Here, we tested performance to predict all-cause mortality after a total follow-up of 402,067 person-years. During this period, 11,098 all-cause deaths were recorded (19%), with higher mortality rates observed in MHAS (1423 deaths, 35%) and HRS (6164 deaths, 26%) (Fig. 1). AnthropoAgeAccel was associated with all-cause mortality (HR 1.05, 95%CI 1.04–1.05 per 1-year increase) independently of CA, sex, race/ethnicity, education, smoking, drinking, and comorbidities, and its addition improved predictive performance (Uno’s c-statistic 0.806 [95%CI 0.791–0.822]) compared to CA alone (0.803 [0.787–0.819], *p* < 0.001 for difference). This was replicated for each study except for MHAS, where the c-statistic for AnthropoAgeAccel + CA was not significantly greater than that of CA (Supplementary Table 8). Across all populations, AnthropoAgeAccel was superior to BRI, WWI, and ABSI after adjusting for covariates (Fig. 2a). Its performance was better in White, Asian, younger, and comorbidity-free participants, and broadly similar across sex and BMI/WHtR quintiles (Supplementary Fig. 11). We removed 2020–2021 from follow-up as a sensitivity analysis to rule out whether distinct mortality patterns during the COVID-19 pandemic influenced results but found no relevant differences (Supplementary Table 9).

We used time-dependent area under curves (tAUC) to assess performance at specific follow-up times (Fig. 2b). Throughout the follow-up (8 years), AnthropoAgeAccel improved the discriminatory capacity of CA alone, which also outperformed BRI, WWI, and ABSI, as shown by the difference between tAUC’s (CA + AnthropoAgeAccel minus CA alone) being consistently greater than zero (Fig. 2c). When disaggregated by study, AnthropoAge had a consistently superior tAUC for HRS, ELSA, CHARLS, and CRELES; however, this was not the case for MHAS (Supplementary Figs. 12–13). Next, we performed a decision curve analysis to assess the net benefit of using each biomarker for mortality risk prediction, which reflects the ability to correctly identify individuals at high mortality risk while minimizing unnecessary interventions (i.e., any action or decision made based on risk assessment) for those at low risk (Fig. 2d). We found that adding AnthropoAgeAccel to CA consistently provided a greater net benefit for mortality risk prediction compared to CA alone, BRI, WWI, and ABSI (Fig. 2d). This added value was further confirmed through ROC curve analysis, where CA + AnthropoAgeAccel showed significantly higher AUCs compared to CA alone (*p* < 0.001), CA + BRI (*p* < 0.001), and CA + WWI (*p* < 0.001) in adjusted models (Supplementary Table 10).

### Accelerated aging and all-cause mortality

**Fig. 3:**
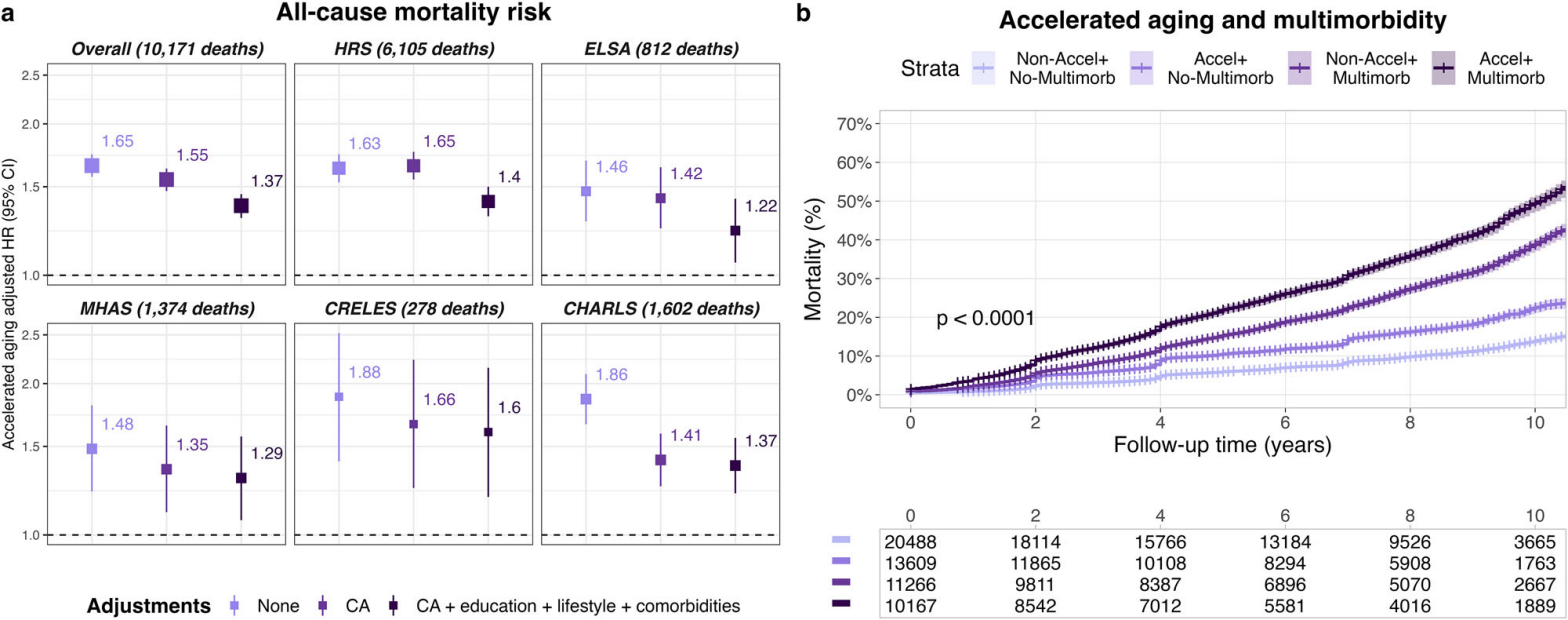
Association of accelerated aging with all-cause mortality. **a** Hazard ratios (HR, 95% CI) for all-cause mortality associated with accelerated aging (AnthropoAgeAccel >0) in the overall G2A sample and stratified by survey. Cox models were stratified by sex and race/ethnicity and sequentially adjusted as follows: 1) unadjusted, 2) adjusted for chronological age (CA), and 3) adjusted for CA, education level, smoking, alcohol consumption, and comorbidities (hypertension, diabetes, myocardial infarction, stroke, cancer, chronic lung disease and arthritis). The top numbers represent adjusted HRs for each group. This analysis was conducted among participants with complete covariate data (*n* = 51,527). **b** Kaplan-Meier curve comparing 10-year cumulative mortality risk by accelerated aging (AnthropoAgeAccel >0) and multimorbidity ( ≥ 2 comorbidities) in the overall G2A sample (*n* = 57,080). *P*-value for a log-rank test for comparison of survival curves.

We observed that weight, waist circumference, BMI, and WHtR exhibited well-documented U-shaped associations with mortality risk^21^. In contrast, AnthropoAge and AnthropoAgeAccel followed distinct patterns: AnthropoAge displayed a monotonically increasing linear relationship with mortality risk, while AnthropoAgeAccel exhibited a sigmoid association, with a steeper risk gradient near the median that attenuated at extreme values (Supplementary Fig. 14). We explored whether accelerated aging (AnthropoAgeAccel >0) could independently predict all-cause mortality risk. Participants with accelerated aging had an increased risk of death independently of CA, sex, race/ethnicity, education level, smoking, drinking, and comorbidities compared to those without accelerated aging in the overall population (HR 1.37 [95% CI 1.30–1.45]), and in HRS (1.40 [1.31–1.50]), ELSA (1.22 [1.06–1.42]), MHAS (1.29 [1.07–1.57]), CRELES (1.60 [1.19–2.15]) and CHARLS (1.37 [1.21–1.56]) individually (Fig. 3a). Accelerated aging was associated with a higher mortality risk regardless of the presence of multimorbidity (two or more comorbidities), and we observed an additive effect, with participants experiencing both accelerated aging and multimorbidity facing the highest mortality risk (Fig. 3b, Supplementary Figs. 15, 16). Lastly, we used longitudinal measurements to account for the effect that changes in AnthropoAgeAccel would have on mortality risk. When comparing the highest vs. lowest AnthropoAgeAccel quartiles, we observed a HR for all-cause mortality of 1.73 (95% CI 1.61–1.87) in the overall population, which was also observed in HRS (1.97 [1.79–2.14]), ELSA (1.79 [1.42–2.25]), CRELES (3.12 [1.93–5.07]) and CHARLS (1.67 [1.41–1.97]) after adjusting for covariates; for MHAS, we noted a significantly higher risk in those with accelerated aging (1.30 [1.10–1.54]) (Supplementary Table 11).

### AnthropoAge trends over time across G2A studies

**Fig. 4:**
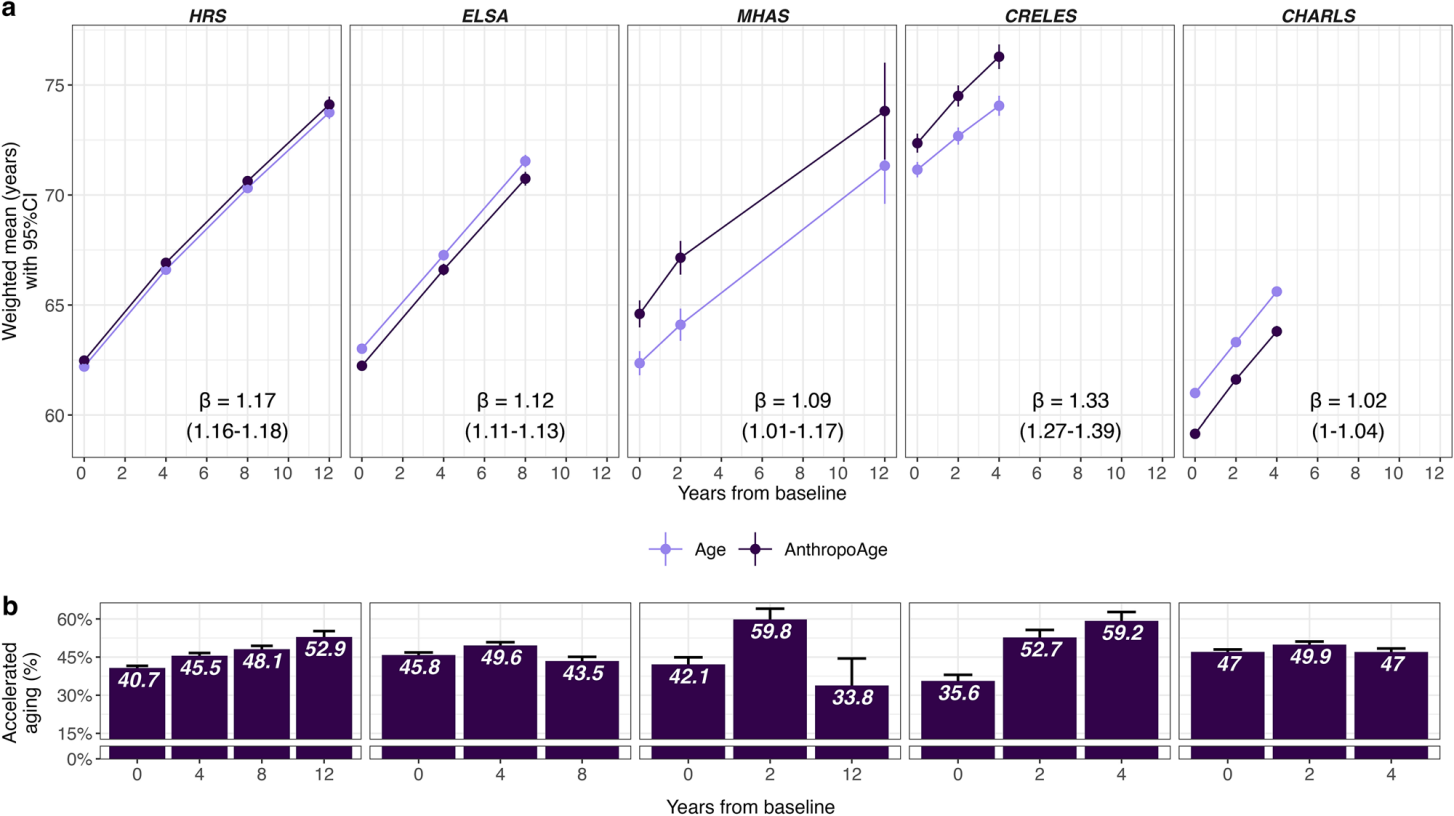
Longitudinal trends in chronological age and AnthropoAge across G2A surveys. **a** Mean chronological age and mean AnthropoAge over follow-up time (years from baseline) in each G2A survey. β-coefficients were estimated with generalized estimating equation models with a Gaussian function; *β* > 1 indicates that the population aged, on average, faster than expected per year of follow-up. Means with 95% confidence intervals were calculated using survey weights. **b** Weighted prevalence with 95% confidence intervals of accelerated aging (AnthropoAgeAccel >0) over follow-up time (years from baseline) for each G2A study.

Next, we characterized changes in AnthropoAge and AnthropoAgeAccel over time. Overall, mean AnthropoAge values increased 12.11 years after 12 years of follow-up, which translated into an increase of 0.24 years in mean AnthropoAgeAccel. Expected changes of these metrics stratified by study and sex across different follow-up times are shown in Supplementary Tables 12, 13. To quantify how faster than expected did AnthropoAge increase over time, we obtained β coefficients from linear GEE models for HRS (*β* = 1.17 [95% CI 1.16–1.18]), ELSA (1.12 [1.11–1.13]), MHAS (1.09 [1.01–1.17]), CRELES (1.33, [1.27–1.39]), and CHARLS (1.02, [1.00–1.04]) (Fig. 4a). After adjusting for covariates, we observed a substantial attenuation of these trends, with the adjustment for comorbidities having the greatest impact (Supplementary Table 14). The prevalence of accelerated aging with each follow-up year gradually increased for HRS and CRELES, although this pattern was not visible in the rest of studies (Fig. 4b). These trends were broadly similar for men and women in ELSA, MHAS, and CHARLS, however, men displayed a steeper increase in AnthropoAge in HRS (β for men 1.09 [1.07–1.10]), while women had a more pronounced change in CRELES (β for women 1.43 [1.35–1.51]) (Supplementary Figs. 17, 18). In all studies, individuals with the highest AnthropoAgeAccel values showed steeper increases in AnthropoAge over time, while those with the lowest AnthropoAgeAccel values had increases that remained below those of CA over time (Supplementary Fig. 19).

### Association of AnthropoAgeAccel with comorbidities, SRH, ADL, and IADL deficits

We observed a significant cross-sectional and longitudinal associations of AnthropoAgeAccel with number of comorbidities and self-reported health, although these associations were more apparent in participants aged <70 years (Supplementary Figs. 20, 21). Thus, we explored the ability of longitudinal AnthropoAgeAccel measures to prospectively predict new onset of comorbidities, ADL/IADL deficits, and changes in self-reported health. We excluded participants who already had the outcome of interest at the time of recruitment (e.g., when assessing diabetes, we excluded participants with a prior diagnosis of diabetes at baseline), allowing us to assess new onset of every condition, defined as a self-report in a subsequent interview. AnthropoAgeAccel (per 1-year increase) predicted new-onset of ADL (1.11 [1.09–1.14]) and IADL deficits (1.13 [1.10–1.15]), as well as new-onset of poor SRH (1.12 [1.09–1.16]), diabetes (1.04 [1.01–1.07]), hypertension (1.03 [1.01–1.06]), stroke (1.06 [1.01–1.12]), cancer (1.09 [1.06–1.12]), and chronic lung disease (1.12 [1.09–1.16]), independently of CA, sex, race/ethnicity, education level, smoking, and alcohol consumption, and these results were also observed for AnthropoAgeAccel > 0 (accelerated aging) (Table 1). We also tested for AnthropoAgeAccel*time interactions, which were significant for ADL/IADL deficits, poor SRH, cancer, and chronic lung disease, where each year of follow-up amplified AnthropoAgeAccel risk by around 2%. For all outcomes, addition of AnthropoAgeAccel led to QIC minimization compared to models with CA alone, indicating improved model performance (Table 1). All associations, except for stroke and myocardial infarction, remained significant after further adjusting for comorbidities (Supplementary Table 15). When disaggregating by G2A study, we observed similar trends for HRS, ELSA, and CRELES; however, for CHARLS and MHAS, AnthropoAgeAccel was only associated with ADL/IADL deficits (Supplementary Fig. 22).

**Table 1:** Longitudinal associations between AnthropoAgeAccel and the risk of new functional and health deficits in the harmonized G2A dataset

*Outcome*	*Population at risk at baseline*	*Events (n)*	^*a*^*Rate Ratio (RR) with 95% CI* ^b^*Odds Ratio (OR) with 95% CI*			*ΔQIC*
			AnthropoAgeAccel (per 1 y increase)	AnthropoAgeAccel x time interaction	AnthropoAgeAccel >0 (accelerated aging)	AnthropoAge Accel + CA vs. CA alone
New onset ADL deficit^a^	46,350	4162	1.114 (1.089–1.141)	1.019 (1.015–1.022)	1.422 (1.316–1.536)	−291.69
New onset IADL deficit^a^	45,215	3930	1.125 (1.101–1.150)	1.019 (1.016–1.023)	1.439 (1.331–1.555)	−289.02
New onset poor SRH^b^	46,893	3052	1.124 (1.091–1.158)	1.022 (1.015–1.029)	1.305 (1.202–1.416)	−111.01
New onset diabetes^b^	45,616	2225	1.041 (1.013–1.071)	1.006 (0.999–1.014)	1.165 (1.067–1.272)	−10.48
New onset hypertension^b^	30,260	3713	1.032 (1.010–1.054)	1.002 (0.996–1.008)	1.120 (1.043–1.204)	−21.81
New onset myocardial infarction^b^	45,049	2797	1.019 (0.997–1.042)	1.003 (0.998–1.008)	1.030 (0.947–1.120)	−8.57
New onset stroke^b^	50,887	966	1.063 (1.011–1.117)	1.006 (0.995–1.016)	1.230 (1.075–1.408)	−3.80
New onset cancer^b^	49,459	1472	1.087 (1.055–1.119)	1.012 (1.005–1.019)	1.315 (1.188–1.456)	−39.94
New onset chronic lung disease^b^	48,501	1526	1.124 (1.087–1.162)	1.024 (1.018–1.030)	1.322 (1.188–1.471)	−60.15

Associations are reported per 1-year increase in AnthropoAgeAccel, with a time interaction, and as a binary outcome (accelerated aging, AnthropoAgeAccel > 0). Outcomes include new ADL/IADL deficits, new onset of poor self-reported health (SRH), and new self-reported diagnosis of age-related comorbidities. Analyses were restricted to participants without each outcome at baseline (population at risk). Odds ratios (OR) with 95% confidence intervals (95% CI) were estimated for SRH and comorbidities using weighted generalized estimating equations with a binomial variance function. For ADL/IADL deficits, rate ratios (RR, 95% CI) were estimated using a Poisson variance function. All models were adjusted for chronological age (CA), sex, race/ethnicity, education level, smoking, and alcohol consumption, and clustered at the individual level. Model comparisons were conducted using differences in quasi-likelihood information criterion (ΔQIC), where negative values indicate improved model fit with the addition of AnthropoAgeAccel.

^a^Rate Ratio.

^b^Odds Ratio.

## Discussion

In this population-based, longitudinal, and cross-nationally harmonized study including 113,436 follow-up assessments of 57,080 participants aged 50–94 years from the U.S., England, Mexico, Costa Rica, and China, we examined the use of AnthropoAge and AnthropoAgeAccel as proxies of BA across different populations. Adding AnthropoAgeAccel to CA improved predictive performance and net benefit for identifying subjects at high mortality risk. This improvement persisted throughout follow-up, but varied across countries, with lower predictive value observed in Hispanic/Latino populations. Each 1-year increase in AnthropoAgeAccel was independently linked to ~5% higher mortality risk. Accelerated aging (AnthropoAgeAccel >0) was associated with ~37% higher mortality risk, ~42% higher rates of new-onset difficulties in ADL/IADL, and higher odds of reporting a poor health status, diabetes, hypertension, stroke, cancer, and lung disease independently of CA, sex, race/ethnicity, education level, smoking, alcohol consumption, and comorbidity profiles in all countries. Additionally, we found that all populations, on average, exhibit faster-than-expected increases in AnthropoAge over time, suggesting trends of age acceleration. Altogether, these results highlight the value of AnthropoAge and AnthropoAgeAccel as potential longitudinal biomarkers of aging and age-related diseases.

Aging is characterized by a progressive deterioration of multiple biological systems over time. Body composition has been proposed as a domain of aging, and sex- and race/ethnicity-dependent changes in anthropometric measures are relevant in aging populations^1,22^. Compared to traditional anthropometric measures, AnthropoAge offers unique advantages in assessing the impact of body composition aging beyond traditional anthropometry. For instance, weight and height increase in early stages of life, but gradually decrease at older ages, likely reflecting adipose tissue redistribution, loss in muscle mass, and decreases in bone mineral density^23–25^. Weight loss can be accompanied by decreases in BMI and adiposity at the expense of visceral adipose tissue accumulation, which is reflected by gradual increases of in waist circumference and WHtR^21,24,26^. In contrast, non-linear relationships for anthropometric measures included in AnthropoAge are able to model the complex interplay of body composition and mortality risk. This finding is supported by our previous work, which demonstrated that AnthropoAge captures a sexual dimorphism in body composition aging driven by age-related changes in sex hormones^23^, whereby accelerated aging in females is associated with body-fat distribution (i.e., increased visceral fat depots and reduced subcutaneous adiposity) and with muscle mass decline and abdominal adiposity in males^17^. Furthermore, both AnthropoAge and AnthropoAgeAccel may be useful to predict age-related outcomes in older adults from various ethnic and sociodemographic backgrounds, as results from the overall G2A population were broadly replicated in participants from HRS, ELSA, CRELES, and CHARLS, although this was not the case for MHAS in most analyses. These findings support that limitations of traditional anthropometric measures to reflect body composition aging may be overcome by explicitly modeling them in complex measures, such as AnthropoAge.

All the hallmarks of aging ultimately lead to functional, cognitive, and physical decline, culminating in disability, dependence, and comorbidity burden in aging individuals^27,28^. Despite the heterogeneity of this process, implementation of BA measures into clinical assessments of older adults remains limited^29^. In contrast, more widely recognized and implemented constructs to assess aging in a clinical setting such as frailty (deficit accumulation and phenotype definitions) and multimorbidity (two or more coexisting diseases) often involve overt clinical manifestations whose progression may be halted but not reversed^7,30,31^, reflecting a persistent decay of interacting physiologic systems, reduced functional and homeostatic reserve, and decreased resilience^30^. Here, we were unable to assess frailty in due to limited availability of these measures in harmonized datasets; however, in a previous work we found that accelerated aging (defined using AnthropoAgeAccel) predicted progression in the frailty phenotype over time in community-dwelling older adults from Mexico^32^. Frailty and multimorbidity may be influenced by molecular and sociodemographic determinants which often overlap with biological aging; this is why BA metrics may also predict frailty risk and increased comorbidity burden^7^ and reflect deterioration of biological systems at a stage where they could potentially be reversed and/or prevented. Furthermore, the aging process in earlier stages of life may present biological changes without overt clinical manifestations, which are often undetected by conventional clinical assessments^12,33^. In the current study, we found that the predictive capacity of AnthropoAge for all-cause mortality was also observed in individuals without multimorbidity, supporting the view that this metric may detect changes in body-composition which underlie biological aging without overt clinical manifestations.

The use of metrics of age acceleration such as AnthropoAgeAccel may aid to detect individuals at the highest risks of developing age-related outcomes. Specifically, we found that subjects in the highest compared to the lowest AnthropoAgeAccel quartile in the United States, England, Costa Rica, and China had a 97%, 79%, 212%, and 67% higher risk of all-cause mortality, respectively. We did not observe this effect in the whole Mexican study; however, the risk increase was 75% when assessing the 2012 cohort. We also demonstrated that AnthropoAgeAccel significantly improves the net benefit of CA for identifying subjects with high mortality risks. These findings support the idea that BA metrics may serve as a valuable alternative to conventional clinical assessments in older adults, and help define accelerated aging as a clinical entity, potentially enabling earlier detection of aging-related changes that could benefit from preventive measures to promote healthier aging^34,35^.

Numerous anthropometry-based markers, such as BMI, WHtR, BRI, WWI, and ABSI^18–20^, have been developed as surrogates of adiposity to predict mortality and health outcomes. While these markers are valuable indicators of adiposity and cardio-metabolic risk, they may not fully capture the complexities of the aging process in body composition. In contrast, AnthropoAgeAccel offers sex-stratified and age-independent estimates of mortality risk, showing a consistent, monotonic increase in risk with higher values of AnthropoAgeAccel, even after adjusting for CA and other covariates. We also observed a consistent risk increase associated to AnthropoAgeAccel, as well as a better improvement in mortality risk prediction when added to CA, compared to the addition of other anthropometric markers. Individuals with accelerated aging (AnthropoAgeAccel > 0) tend to have lower weight and height, but larger waist circumference and WHtR, compared to those with non-accelerated aging, consistent with findings from our previous work^17^. Additionally, AnthropoAgeAccel captures the trajectories of comorbidity profiles over time, particularly in younger populations. Taken together, these findings suggest that AnthropoAge and AnthropoAgeAccel offer a predictive advantage over traditional adiposity markers. Their value for future aging research and their ability to capture distinct aspects of biological aging, beyond what is captured by conventional anthropometry, may be due in part to the non-linear relationships incorporated in their calculation, and the explicit consideration of the effect of CA in its estimation, which is lacking for traditional anthropometric biomarkers.

Recent aging research has focused on the development of longitudinal biomarkers of aging, particularly through the implementation of multi-omics (e.g., third generation epigenetic clocks). However, large-scale implementation of these BA metrics in epidemiological studies remains limited due to costs and inaccessibility; furthermore, given that aging is a heterogeneous process, biomarkers of aging often evaluate distinct underlying processes^9,15^. Thus, BA metrics based on simple and accessible measurements may provide an easy overview of individual aging trajectories across different body systems^1,2^. Here, we show that AnthropoAge and AnthropoAgeAccel and their changes over time are useful to detect mortality risk, declines in self-reported health and functionality, and new onset of age-related diseases over time, indicating that these metrics may be useful longitudinal biomarkers of aging. Future studies elucidating the relationship between AnthropoAge and additional outcomes not assessed in this study, such as cognitive decline and dementia, psychiatric conditions, falls, and sensory impairments (e.g., hearing or vision loss), will further demonstrate the clinical value of our metric. Similarly, detailed research focused on the molecular mechanisms of aging (e.g., telomere length, DNA methylation, metabolomic and genomic data, etc.) captured by AnthropoAge and AnthropoAgeAccel could be used to characterize the pathways linking phenotypic changes to hallmarks of biological aging.

Our results also represent a proof-of-concept of how the implementation of readily available BA biomarkers may be useful to track trends of biological aging at the population level, an approach which has the potential to contribute to the understanding of exposures that influence age-related outcomes in diverse populations^36^. The changes in AnthropoAge observed in our study likely reflect the potential impact of changing health behaviors and the increasing prevalence of chronic disease. Further studies exploring the determinants and modifiers of accelerated aging are warranted. Although AnthropoAge was created to estimate mortality, previous research has shown that the use of prospective data in second-generation aging biomarkers may yield a better performance to detect age-related outcomes^37^, as demonstrated in our study.

Our study had several strengths, including a population-based multi-national sample of older individuals which is representative at the national level, which allowed us to externally validate AnthropoAge across several race/ethnicities and socioeconomic backgrounds as a marker of mortality and age-related outcomes independently of common determinants of healthy aging. Second, by combining statistical methods developed to evaluate individual-level prediction with population-level average effects using longitudinal data, we were able to show that AnthropoAge may be a useful longitudinal aging biomarker and that its performance in selected populations (i.e., Hispanic/Latino individuals) may warrant further recalibrations to account for ethnic differences in the contribution of body composition to mortality risk. Third, by exploring an accessible biomarker of aging which can be reproduced in large-scale epidemiological studies, we were able to show that AnthropoAge detects changes in the rate of biological aging over time at the population level. However, we should also acknowledge some limitations which have to be considered to adequately frame the results of our work. By using harmonized datasets from the G2A platform and considering the effect of the study in all statistical analyses we were able to reduce differences between cohorts; however, intrinsic cultural and geographic differences may influence interpretations of results, furthermore, because definitions of exposures and outcomes are heterogeneous across different G2A studies, some variability was observed. For instance, the item “walking across the room” was unavailable in CHARLS and was substituted for “dressing” for ADL assessment, additionally, mortality ascertainment was done via verbal autopsy by proxy interview for all studies except CRELES, for which death registry linkage was used; these differences may introduce differential measurement errors and increase bias and variability when directly comparing results across G2A studies. Additionally, comorbidities were determined based on self-reports from each assessment, using the question, ‘Has a doctor ever told you that you have…?’ This approach may introduce inaccuracies, particularly for conditions with complex diagnostic criteria or limited screening practices^38,39^. Furthermore, we included only diseases available across all studies to enhance comparability; however, this prevented us from accounting for important confounders related to mortality and age-related outcomes, such as chronic kidney disease and neurological disorders. Follow-up times for longitudinal assessments were determined based on interview dates, making it impossible to pinpoint the exact timeframe in which a participant developed a deficit or disease. Additionally, the end of follow-up was defined using approximate dates of death, as reported by a relative of the deceased participant, introducing a potential source of bias. Finally, because we implemented the simplified version of AnthropoAge, we may not be able to fully capture the spectrum of body composition aging reflected by the full measure with more diverse anthropometric measurements.

In conclusion, AnthropoAge and AnthropoAgeAccel are useful proxies of BA and accelerated aging which capture mortality risk, functional decline, and risk of age-related diseases in diverse populations. We showed that longitudinal assessments of age acceleration capture mortality risk, functional decline, and risk of chronic diseases independent of CA, comorbidities, education, and lifestyle, and that AnthropoAgeAccel detects individuals at a particularly high risk. Implementation of AnthropoAge in population-based longitudinal studies may allow for modeling trends of biological aging, permitting accessible assessment of faster or slower rates of aging at the population level for planning of public health interventions. Lastly, despite its strong overall and time-dependent performance for prediction of mortality, we observed heterogeneous results across different races/ethnicities, and recalibration of AnthropoAge may be warranted to improve its applicability in populations which are often underrepresented in aging studies, including Latin American, Southeast Asian, and African older adults.

## Methods

### Data access

The Gateway to Global Aging Data platform (G2A, https://g2aging.org/) gathers studies from multiple countries designed to be comparable with each other; these studies are longitudinal, multidisciplinary, and nationally representative of adults ≥50 years. We included individuals with complete data on core interviews, anthropometry, and linked mortality from HRS^40^, ELSA^41^, MHAS^42^, CRELES^43^, and CHARLS^44^. An overview of datasets, timepoints, and references to additional documentation is provided in Supplementary Methods.

### Subsets of anthropometric data

In addition to demographic, socioeconomic, and health-related questionnaires, a subset of participants underwent biochemical and physical measurements (including anthropometry) that were conducted at selected cycles:

- HRS: participants were eligible for anthropometry every two waves from 2006 to 2018 (waves 8–14) among each half of respondents (i.e., one half had anthropometry for waves 8, 10, 12, and 14, while the other half for waves 9, 11, and 13)^40^.
- ELSA: all participants from waves 2, 4, and 6 (2004, 2008, and 2012) were eligible for anthropometry^41^.
- MHAS: a random subsample of participants from waves 1 and 2 (2001, 2003) was eligible for anthropometry; however, measurements were restricted to four states (one highly urbanized, one with high-migration, one relatively poor and one with a high prevalence of diabetes) in participants from wave 3 (2012)^42^.
- CRELES: all participants were eligible for anthropometry, however, we focused exclusively on participants from waves 1–3 (2005, 2007, 2010), which comprised the original pre-1945 cohort^43^ (see Supplementary Methods).
- CHARLS: participants from waves 1, 2, and 3 (2011, 2013, and 2015) were eligible for anthropometry^44^.

With this data, we used measured height, weight, and waist circumference to calculate BMI (weight in kilograms divided by squared height in meters) and WHtR (waist in centimeters divided by height in centimeters); self-reported anthropometry was not used. An overview of measuring techniques in each G2A study is provided in Supplementary Methods.

### AnthropoAge and AnthropoAgeAccel

We previously developed and validated AnthropoAge in the National Health and Nutrition Examination Survey (NHANES) as a proxy of BA^17^. AnthropoAge was derived from CA and anthropometry to predict 10-year mortality risk using two proportional hazards models following the Gompertz distribution: 1) Using only CA as predictor. 2) Using CA and non-linear contributions of anthropometric measurements. Both models were stratified by sex, and the second model included race/ethnicity (Non-Hispanic White, Non-Hispanic Black, Hispanic/Latino, Other) in the shape parameter of the distribution. For this study, we used the simplified version of AnthropoAge, which uses CA, BMI, and WHtR.

To obtain an estimate of BA in age units, we assumed the cumulative distribution functions (CDFs) of each model to be approximately equal:

$${CDF}\left(120,\,{{age}}_{i}\right)\approx {\rm{CDF}}\left(120,\,{{xb}}_{i}\right)$$

Where ${{age}}_{i}$ is the linear combination of coefficients from the Gompertz model including only CA, and ${{xb}}_{i}$ the linear combination coefficients from the model including both CA and anthropometry. The solution to this equation is the i-th individual’s AnthropoAge, which can be interpreted as the risk of dying within the next 10 years predicted by CA and anthropometry, in units of age.

$${\rm{AnthropoAge}}=\frac{\mathrm{ln}\left(-\frac{\mathrm{ln}(1-{\rm{M}})}{{{{\rm{\gamma }}}_{0}}^{-1}\left({{\rm{e}}}^{{{\rm{\gamma }}}_{0}{\rm{t}}}-1\right)}\right)-{{\rm{\beta }}}_{0}}{{{\rm{\beta }}}_{1}},t > 0$$

Where:

- *t* is follow-up time in months.
- γ_0_ (shape), β_0_ (rate), β_1_ (beta coefficient for CA), are the parameters of the Gompertz regression using only CA as a predictor.
- M = ${{CDF}}_{2}\left(120,\,{{xb}}_{i}\right)=1-{{\rm{e}}}^{-{({\rm{e}}}^{\left({\rm{xb}}\right)}{{{\rm{\gamma }}}_{{\rm{j}}}}^{-1}\,({{\rm{e}}}^{{{\rm{\gamma }}}_{{\rm{j}}}{\rm{t}}}-1))}$, the CDF of the model that uses both CA and anthropometry as predictors.
  - γ_j_ = shape for each j-race/ethnicity.
  - ${xb}$ = combination of rate + CA + body mass index (BMI, log-transformed, 2 polynomial degrees) + waist to height ratio (WHtR, cubic root-transformed).

A full table with precise coefficients to calculate AnthropoAge is available in Supplementary Methods.

To measure biological age acceleration, we estimated AnthropoAgeAccel by removing the effect of CA from AnthropoAge^17,45^. Briefly, we regressed AnthropoAge onto CA and obtained the residuals using a mixed effects linear model, where we included a random intercept for participant ID to account for intra-individual dependence of longitudinal data, and a second random intercept for G2A study to reduce potential clustering of the effect in each country. For analyses using only baseline assessments (e.g., mortality risk associated to baseline AnthropoAge), only a random intercept for G2A study (but not for participant ID) was used. This was done separately for men and women to address sex-based differences in body composition and aging^46^. Accelerated aging was defined as AnthropoAgeAccel values > 0, while non-accelerated or delayed aging was defined with values ≤ 0. We also used AnthropoAgeAccel quartiles to assess participants with more pronounced deviations from CA. All materials and code used to estimate AnthropoAge and AnthropoAgeAccel are available in the *AnthropoAge* R package (https://github.com/oyaxbell/AnthropoAgeR). Additionally, we developed a Shiny App to facilitate broader use of AnthropoAge, allowing both individual and batch calculations via CSV upload (https://bellolab.shinyapps.io/anthropoage/).

### Classification of race and ethnicity

To address differences in anthropometry across populations, AnthropoAge uses race/ethnicity in the shape parameter of the Gompertz distribution. In HRS, the assessment of race and ethnicity adheres to the Office of Management and Budget Standards^47,48^, based on this, we used a race/ethnicity classification where HRS participants were coded as either (Non-Hispanic) White, (Non-Hispanic) Black, Hispanic/Latino or Other (which includes Asian, Native American, or Pacific Islander); given the lack of consensus to define race and ethnicity across the world, the rest of surveys included in this study also follow this classification: participants from ELSA were coded as White, those from MHAS and CRELES as Hispanic/Latino, and those from CHARLS as Other (referring to Asian, and more specifically, Chinese participants).

### Functionality, general health status, and comorbidities

Functional decline in older adults is closely related to the diagnosis of cognitive impairment and dementia^49–51^ and is associated with a higher mortality risk in this population^52^. Here, we used self-reported deficits in activities of daily living (ADL) and instrumental activities of daily living (IADL)^53^ to assess the functional status of participants. Although there are validated clinical scales to appraise ADL/IADL^54^, we only included a few items to enable comparisons across G2A studies. For ADL deficits, patients were asked if they had some difficulty in performing any of the following: bathing, eating, transferring in and out of bed, using the toilet, and walking across the room. For IADL deficits, the same question was asked about: managing money, taking medication, shopping for groceries, and preparing hot meals. Based on this, we created scores ranging from 0–5 (ADL) and 0–4 (IADL) representing the number of activities in which participants had any difficulty. In the case of CHARLS, “walking across the room” was unavailable and was substituted for “dressing” to facilitate comparisons using five ADL items (Supplementary Material). Participants’ self-reported general health status was evaluated across all cohorts by asking “Would you say your health is”: 1) excellent, 2) very good, 3) good, 4) fair, 5) poor. Self-reported diagnoses of hypertension, diabetes, cancer, chronic lung disease, myocardial infarction, stroke, and arthritis were included, as these were the only conditions consistently available across all G2A cohorts, ensuring comparability. Each disease was used individually as both an outcome and an adjustment factor, while multimorbidity ( ≥ 2 comorbidities) was used as a stratification factor in select analyses.

### End-of-life data

For HRS, ELSA, MHAS, and CHARLS, mortality was ascertained via a verbal autopsy by a family member proxy during an interview in a follow-up wave. For CRELES, all Costa Rican citizen participants were linked to the birth and death registries using their ID-card number. HRS and MHAS collected information on mortality status up to the year 2021, however, this information was only available up to 2020 for CHARLS, 2012 for ELSA and 2010 for CRELES (Fig. 1). All studies collected the approximate date of death of deceased individuals, which was used along with date of baseline interview to estimate follow-up time in person-years (date of last interview was used for deceased individuals for whom date of death was not available). Individuals who had not died at the end of follow-up were censored.

### Statistical analysis

All analyses were restricted to individuals aged 50–94 years with complete mortality and anthropometric data. Participants also had to have anthropometric measurements within the ranges observed in NHANES, where AnthropoAge was originally developed (height: 125–200 cm, weight: 30–150 kg, waist: 50–160 cm, BMI: 10–60 kg/m^2^), to ensure stable estimations of AnthropoAge. Analysis of missing data patterns showed that missingness in anthropometry, outcomes, and covariates (education, smoking, and alcohol consumption) was higher among older individuals and those who died. Because of this, we considered that multiple imputation was likely to represent a source of bias and opted instead for a complete case analysis. A full report of missing data can be found in Supplementary Figs. 6–10, and in Supplementary Tables 2–6.

### Assessment of AnthropoAge predictive performance

We validated the capability of baseline AnthropoAgeAccel to predict all-cause mortality using sex-stratified Cox proportional hazard regression models. Predictive performance was evaluated using Uno’s c-statistic (*survival* R package^55^), and time-dependent areas under the receiver operating characteristic curves (tAUC) (*riskRegression* R package^56^), both of which are based on the non-parametric approach of inverse-probability of censoring weights^57,58^. Uno’s c-statistic was used to assess the performance of AnthropoAge over the entire follow-up period (overall and stratified by race/ethnicity, sex, age, BMI, and WHtR); while tAUC was used to assess its performance at specific follow-up times (up to 12 years for HRS and MHAS, 8 years for ELSA and CHARLS, and 4 years for CRELES). Comparisons of c-statistics were conducted using non-parametric z-score tests (*survcomp* R package^59^). Results were computed for all the studies combined (overall population), as well as for each individual G2A study separately. We next estimated AUCs for 10-year all-cause mortality using Cox models with varying levels of adjustment, with CA as the predictor. We tested whether the addition of AnthropoAgeAccel significantly improved AUC using nonparametric ROC tests with bootstrapping (*b* = 1000). For these analyses, we compared AnthropoAge and AnthropoAgeAccel to other anthropometry-based biomarkers, namely the Body Roundness Index (BRI), Weight-Adjusted Waist Index (WWI), and A Body Shape Index (ABSI), whose calculation has been described elsewhere^18–20^. We tested the proportional hazards assumption of fully adjusted models using the proportional hazards test (*survival* R package), and by visualizing Schoenfeld residuals for AnthropoAgeAccel (Supplementary Fig. 23) and accelerated aging (Supplementary Fig. 24). Briefly, we observed that the assumption was fulfilled for the individual variables and global models in ELSA, MHAS, CRELES, and CHARLS. For the overall population and HRS, although the tests yielded *p*-values < 0.05, no substantial trends were observed in the Schoenfeld residuals, these results are likely due to minor deviations in a large sample size.

### Decision curve analysis

We performed decision curve analysis (DCA) to evaluate the clinical utility of the predictive models by assessing their net benefit across a range of mortality risk thresholds. This approach accounts for the trade-off between correctly identifying individuals at high mortality risk and minimizing unnecessary interventions for those at low risk. The DCA compared a baseline model (sex, ethnicity, cohort, education level, smoking, alcohol intake) to models adding different predictors: BRI, WWI, ABSI, CA alone, and CA + AnthropoAgeAccel. For each model, the net benefit was plotted as a function of the threshold probability, representing the minimum predicted risk at which an individual would be considered for intervention (*dcurves* R package)^60^.

### Accelerated aging and all-cause mortality risk

We estimated the hazard ratio (HR) with 95% confidence intervals (95% CI) for all-cause mortality for accelerated aging (AnthropoAgeAccel >0) and AnthropoAgeAccel quartiles using Cox proportional hazard regression models with standardized survey weights. These models were stratified by sex and race/ethnicity and sequentially adjusted for CA, education level (primary or less, secondary, tertiary), smoking (never, former smoker, <10 cigarettes/day, ≥10 cigarettes/day), alcohol consumption (never, less than weekly, less than daily, daily) and comorbidities (hypertension, diabetes, myocardial infarction, stroke, cancer, chronic lung disease, arthritis). This analysis was performed on baseline measurements, however, we also used time varying Cox models clustered by ID to account for longitudinal measurements as a sensitivity analysis. Lastly, to visualize the additive effect of comorbidities and accelerated aging on mortality risk, we used Kaplan-Meier curves stratified by the presence of accelerated aging and multimorbidity ( ≥ 2 comorbidities).

### Changes in AnthropoAge and AnthropoAgeAccel over time

To evaluate trends of AnthropoAge as a function of time, we fitted weighted generalized estimating equation (GEE) models with a Gaussian variance function, where AnthropoAge was the dependent variable and years of follow-up was the predictor; robust sandwich standard errors and an autoregressive correlation structure (measures closer in time are more correlated) were used (*geepack* R package^61,62^). If this model had a β-coefficient >1, we considered that the rate of population aging occurred, on average, faster than expected with each year of follow-up, on the other hand, if the β-coefficient was <1 then the population aged, on average, slower than expected with each year of follow-up. These trends were visualized using weighted means of age and AnthropoAge over follow-up time (years from baseline). We also conducted a sensitivity analysis stratifying these trends by sex and AnthropoAgeAccel quartiles. To identify whether covariates had an effect on age acceleration, we sequentially adjusted GEE models for sex, race/ethnicity, education, smoking, alcohol consumption, and comorbidities.

### Association of AnthropoAgeAccel with ADL, IADL, and comorbidities

We hypothesized that changes of AnthropoAgeAccel would be predictive of functional decline, changes in self-reported health, and new onset of chronic age-related diseases independently of CA. To test this, we fitted weighted GEE models with robust sandwich standard errors and autoregressive correlation structures. We used a Poisson variance function to estimate rate ratios (RR) for counts of ADL/IADL deficits, and a binomial variance function to estimate odds ratios (OR) for reporting a poor SRH and new diagnosis of chronic diseases during follow-up. All models were fully adjusted as specified above and considered clustering at an individual level. For all outcomes, we fitted a GEE model with only CA as a predictor and a second model adding AnthropoAgeAccel, and compared them using the quasi-likelihood information criterion (QIC), where negative values of the QIC difference between models (ΔQIC = QIC_M2_ – QIC_M1_) indicate that the addition of AnthropoAgeAccel improves the model’s fit. In the overall G2A analysis, these models were fitted in population at-risk (i.e., we excluded participants who already had the outcome at baseline) to identify new onset of functional deficits and comorbidities. However, when we stratified by country, we did not exclude individuals with the conditions at baseline to increase statistical power. All statistical analyses were conducted using R version 4.2.1, and *p*-value thresholds are estimated for a two-sided significance level of *α* = 0.05.

## Supplementary information

Supplementary Material_V4.0

## Data Availability

All datasets and materials are available for reproducibility of results at https://github.com/oyaxbell/g2a_anthropoage/.

## Code availability

All code are available for reproducibility of results at https://github.com/oyaxbell/g2a_anthropoage/.
