# Supplementary material for: Multinational evaluation of AnthropoAge as a measure of biological age in the USA, England, Mexico, Costa Rica, and China: a population-based longitudinal study
Source: NPJ Aging. 2025 Jun 21;11(1):52. doi: 10.1038/s41514-025-00232-1 (PMC12182590; doi:10.1038/s41514-025-00232-1)

<u>SUPPLEMENTARY MATERIAL</u> - Multinational evaluation of AnthropoAge as a measure of biological age in the USA, England, Mexico, Costa Rica, and China: a population-based longitudinal study

Carlos A. Fermín-Martínez, Daniel Ramírez-García, Neftali Eduardo Antonio-Villa, Jerónimo Perezalonso Espinosa, Diego Aguilar-Ramírez, Carmen García-Peña, Luis Miguel Gutiérrez-Robledo, Jacqueline A. Seiglie, Omar Yaxmehen Bello-Chavolla

SUPPLEMENTARY METHODS

Overview of G2A longitudinal surveys

The following overview of the Gateway to Global Aging (G2A) surveys was obtained from the G2A Health and Retirement Studies overview page (https://g2aging.org/survey/overview), as well as from the individual cohort profiles of each study.

- ***Health and Retirement Study (HRS)***[1]**:**
  - Started in 1992 and is currently comprised of 15 biennial waves (last one in 2020).
  - Nationally representative data of adults aged ≥51 years from private households in the U.S., with oversampling for African American and Hispanic households.
  - Currently comprised of 8 cohorts (original HRS sample plus 7 refreshments), of which only 3 are included in our study due to availability of anthropometric data.
  - Anthropometry is available from waves 8-14 every two waves among each half-sample of respondents (i.e., one half of participants has anthropometry for waves 8, 10, 12 and 14, while the other half has anthropometry for waves 9, 11 and 13).

- ***English Longitudinal Study of Ageing (ELSA)***[2]**:**
  - Started in 2002, comprised of 9 biennial waves (last in 2019); end of life interviews were only conducted up to wave 6 (2012).
  - Nationally representative, adults from England aged ≥50 years, no oversampling.
  - Comprised of 6 cohorts (original plus 5 refreshments), only 3 included in our study.
  - Anthropometry is available in waves 2, 4 and 6 (2004, 2008 and 2012). It is also available for 50% of participants in wave 8 and for the remaining 50% of participants in wave 9; however, we did not include them in our study due to missing mortality data.

- ***Mexican Health and Aging Study (MHAS)***[3]***:***
    - Started in 2001, comprised of 5 waves (2001, 2003, 2012, 2015, 2019).
    - Nationally representative at both rural and urban levels, adults from Mexico aged ≥50 years, oversampling (original cohort) in the six states that accounted for 40% of all migration to the USA (Durango, Guanajuato, Jalisco, Michoacán, Nayarit, Zacatecas) (https://enasem.org/Documentation/SurveyDesign_Esp.aspx).
    - Comprised of 3 cohorts (original plus 2 refreshments), only 2 included in our study.
    - Anthropometry available in a random subsample of participants from waves 1-2, and in the full sample of four states (one highly urbanized, one with high-migration, one relatively poor and one with a high prevalence of diabetes) from wave 3.

- ***Costa Rican Longevity and Healthy Aging Study (CRELES)***[4]***:***
    - Original CRELES pre-1945 cohort.
        - Includes waves 1-3 (2004-2006, 2007-2008, 2009-2010).
        - Nationally representative sample of Costa Rican adults born before 1945 (aged ≥60 years), with oversampling in participants aged ≥95 years old.
        - All participants were eligible for anthropometric measurements.
        - This is the cohort we included in our study.
    - CRELES 1945-1955 retirement cohort.
        - Includes waves 4-5 (2010-2011, 2012-2014).
        - Nationally representative of Costa Rican adults born between 1945 and 1955 (aged 55 to 65 years old), with no oversampling.
        - All participants were eligible for anthropometric measurements.
        - This cohort was excluded from our study.

- ***China Health and Retirement Longitudinal Study (CHARLS)***[5]***:***
    - Started in 2011, comprised of 4 waves (2011, 2013, 2015, 2019).
    - Nationally representative at both rural and urban levels, adults from China aged ≥45 years, with no oversampling.
    - Comprised of 4 cohorts (original plus 3 refreshments), only 3 included in our study.
    - All participants from waves 1-3 were eligible for anthropometric measurements.

Assessment of anthropometric variables across G2A studies

<table>
<tr><th colspan="2">Measurement</th><th>HRS</th><th>ELSA</th><th>MHAS</th><th>CRELES</th><th>CHARLS</th></tr>
<tr><td rowspan="2">Weight</td><td>Availability</td><td>Waves 8-14</td><td>Waves 2,4,6</td><td colspan="3">Waves 1-3</td></tr>
<tr><td>Technique</td><td>Healthometer 830KL</td><td>Not detailed</td><td>Electronic portable scales</td><td>Life source scale</td><td>Unspecified scale</td></tr>
<tr><td rowspan="2">Height</td><td>Availability</td><td>Waves 8-14</td><td>Waves 2,4,6</td><td colspan="3">Waves 1-3</td></tr>
<tr><td>Technique</td><td>Tape measure</td><td colspan="4">Stadiometer</td></tr>
<tr><td rowspan="2">Waist circumference</td><td>Availability</td><td>Waves 8-14</td><td>Waves 2,4,6</td><td colspan="3">Waves 1-3</td></tr>
<tr><td>Technique</td><td>Height of navel</td><td>Not detailed</td><td colspan="2">Midway between iliac crest and costal margin</td><td>Height of navel</td></tr>
</table>

AnthropoAge calculation

AnthropoAge was developed to predict 10-year all-cause mortality based on the work by Levine et al.[6] using two parametric proportional hazards models following the Gompertz distribution:

1. Using only chronological age (CA) as the predictor.
2. Using CA and anthropometric measurements (weight, height, and waist in the simplified version).

Both models were stratified by sex, and the second model included race/ethnicity (Non-Hispanic White, Non-Hispanic Black, Hispanic/Latino, Other) in the shape parameter of the distribution.

In order to obtain an estimate of biological age (BA) in age units, we assumed the cumulative distribution functions (CDF's) of each model to be approximately equal, implying:

$$CDF(120, age_i) \approx \mathrm{CDF}(120, xb_i)$$

Where age_i is the linear combination of coefficients from the Gompertz model including only CA, and xb_i the linear combination coefficients from the model including both CA and anthropometric measurements. The solution to this equation is the i-th individual's AnthropoAge, which is implied to be a function of the CDF at 120 months for the linear combination of anthropometric measurements (i.e., the risk of dying within the next 10 years predicted by CA and anthropometric measurements), expressed in units of age.

$$\text{AnthropoAge} = \frac{\ln\left(-\frac{\ln(1-\text{M})}{\gamma_0^{-1}(e^{\gamma_0 t}-1)}\right) - \beta_0}{\beta_1}, \quad t > 0$$

Where:

- t is the follow-up time in months.
- γ_0 (shape), β_0 (rate), β_1 (beta coefficient for CA), are the parameters of the Gompertz regression using only CA as a predictor (model 1).

Sex	γ_0	β_0	β_1
Men	0.00585	-11.28580	0.07811
Women	0.00736	-12.39100	0.08600

- M = $CDF_2(120, xb_i)$, the CDF of the model that uses CA and anthropometric measurements as predictors (model 2).

$$CDF_2(120, xb_i) = 1 - e^{-(e^{(xb)}\gamma_j^{-1}(e^{\gamma_j t}-1))}$$

  - γ_j = shape for each j-race/ethnicity.
  - xb = combination of rate + CA + body mass index (BMI, log-transformed, 2 polynomial degrees) + waist to height ratio (WHtR, cubic root-transformed).

Sex	***Shape γ_j***	***xb***	
		Parameter	*Coefficient*
Men	▪ NH White: 0.00605<br>▪ NH Black: +0.00098<br>▪ Hispanic: -0.00009<br>▪ Other: -0.00440	Rate<br>CA<br>BMI (OP,1)<br>BMI (OP,2)<br>WHtR	-19.0818<br>0.0733<br>-26.6759<br>12.3235<br>9.7851
Women	▪ NH White: 0.00772<br>▪ NH Black: +0.00039<br>▪ Hispanic: -0.00079<br>▪ Other: -0.00255	Rate<br>CA<br>BMI (OP,1)<br>BMI (OP,2)<br>WHtR	-19.2580<br>0.0818<br>-20.8035<br>9.2458<br>8.5259

Assessment of functionality across G2A studies

Activities of Daily Living (ADL) and Instrumental Activities of Daily Living (IADL) are commonly used to evaluate functional status and independence in multidimensional geriatric assessments[7]. ADLs comprise fundamental tasks regarding self-care and well-being (i.e., being able to bathe, eat, and use the bathroom), while IADLs are more complex tasks that involve executive functions and are essential to sustain independent life within home and in the community[8]. Although there are several validated clinical scores such as the Katz Index[9] or the Lawton and Brody scale[10] to assess ADL and IADL, only the following items were available across the G2A cohorts used in this study:

Metric	Activity	HRS	ELSA	MHAS	CRELES	CHARLS
ADL	**Bathing**	Waves 1-15	Waves 1-9	Waves 1-4	Waves 1-5	Waves 1-4
	**Eating**	Waves 1-15	Waves 1-9	Waves 1-4	Waves 1-5	Waves 1-4
	**Transferring in/out of bed**	Waves 1-15	Waves 1-9	Waves 1-4	Waves 1-5	Waves 1-4
	**Using toilet**	Waves 2-15	Waves 1-9	Waves 1-4	Waves 1-5	Waves 1-4
	**Walking across a room**	Waves 1-15	Waves 1-9	Waves 1-4	Waves 1-5	-
	Dressing	Waves 1-15	Waves 1-9	Waves 1-4 (not in proxy interviews)	-	Waves 1-4
	Sphincter control	-	-	-	-	Waves 1-4
IADL	**Managing money**	Waves 2-15	Waves 1-9	Waves 1-5	Waves 1-5	Waves 1-4
	**Taking medication**	Waves 2-15	Waves 1-9	Waves 1-5	Waves 1-5	Waves 1-4
	**Shopping for groceries**	Waves 2-15	Waves 1-9	Waves 1-5	Waves 1-5	Waves 1-4
	**Preparing hot meals**	Waves 2-15	Waves 1-9	Waves 1-5	Waves 1-5	Waves 1-4
	Using a telephone	Waves 2-15	Waves 1-9	-	-	Waves 2-4
	Using a map	Waves 1-15	Waves 1-9	-	-	-

These variables were coded 0 if participants had no difficulty and 1 if they reported some difficulty in each activity. Based on this, we constructed scores to assess ADL and IADL by summing the variables that were available across all five studies. Scores were constructed in participants in whom at least one item was not missing, as recommended in the G2A Harmonized Codebooks (available from https://g2aging.org/hrd/get-data):

- ***ADL score*** (range: 0-5) = Bathing + Eating + Transferring in/out of bed + Using the toilet + Walking across the room.
  - For CHARLS, we used dressing instead of walking across the room.
- ***IADL score*** (range: 0-4) = Managing money + Taking medications + Shopping for groceries + Preparing hot meals.

Ethical disclosures for G2A studies

- HRS was approved by the Health Sciences/Behavioral Sciences Institutional Review Board at the University of Michigan.
- ELSA was approved by the National Health Service Research Ethics Committees under the National Research and Ethics Service.
- MHAS was approved by the Institutional Review Board or Ethics Committee of the University of Texas Medical Branch, the National Institute of Statistics and Geography, and the National Institute of Public Health.
- CRELES was approved by the Ethical Science Committee of the University of Costa Rica.
- CHARLS was approved by the Biomedical Ethics Review Committee of Peking University.

References

1. Sonnega, A. *et al.* Cohort Profile: the Health and Retirement Study (HRS). *Int J Epidemiol* **43**, 576–585 (2014).
2. Steptoe, A., Breeze, E., Banks, J. & Nazroo, J. Cohort profile: the English longitudinal study of ageing. *Int J Epidemiol* **42**, 1640–1648 (2013).
3. Wong, R., Michaels-Obregon, A. & Palloni, A. Cohort Profile: The Mexican Health and Aging Study (MHAS). *Int J Epidemiol* **46**, e2 (2017).

4. Rosero-Bixby, L., Dow, W. H. & Brenes, G. Costa Rican Longevity and Healthy Aging Study. in *Encyclopedia of Gerontology and Population Aging* (eds. Gu, D. & Dupre, M. E.) 1192–1196 (Springer International Publishing, Cham, 2021). doi:10.1007/978-3-030-22009-9_334.
5. Zhao, Y., Hu, Y., Smith, J. P., Strauss, J. & Yang, G. Cohort profile: the China Health and Retirement Longitudinal Study (CHARLS). *Int J Epidemiol* **43**, 61–68 (2014).
6. Levine, M. E. *et al.* An epigenetic biomarker of aging for lifespan and healthspan. *Aging (Albany NY)* **10**, 573–591 (2018).
7. Elsawy, B. & Higgins, K. E. The geriatric assessment. *Am Fam Physician* **83**, 48–56 (2011).
8. Pashmdarfard, M. & Azad, A. Assessment tools to evaluate Activities of Daily Living (ADL) and Instrumental Activities of Daily Living (IADL) in older adults: A systematic review. *Med J Islam Repub Iran* **34**, 33 (2020).
9. Katz, S., Downs, T. D., Cash, H. R. & Grotz, R. C. Progress in development of the index of ADL. *Gerontologist* **10**, 20–30 (1970).
10. Lawton, M. P. & Brody, E. M. Assessment of older people: self-maintaining and instrumental activities of daily living. *Gerontologist* **9**, 179–186 (1969).

SUPPLEMENTARY TABLES

Supplementary Table 1. Baseline sociodemographic, anthropometric, functional assessment, health, and mortality data of participants aged 50-94 years (overall and by cohort). Continuous variables are presented as median (interquartile range), and categorical variables as n (%).

Attribute	Overall N = 57,080	HRS N = 23,301	ELSA N = 11,089	MHAS N = 4,038	CRELES N = 2,208	CHARLS N = 16,444
Sociodemographic data						
**Age (years)**	61 (55, 70)	62 (56, 71)	62 (56, 71)	61 (55, 68)	75 (69, 82)	59 (53, 66)
**Sex (%)**						
Men	25,933 (45%)	10,086 (43%)	5,010 (45%)	1,825 (45%)	982 (44%)	8,030 (49%)
Women	31,147 (55%)	13,215 (57%)	6,079 (55%)	2,213 (55%)	1,226 (56%)	8,414 (51%)
**Race/ethnicity (%)**						
Non-Hispanic White	25,818 (45%)	14,729 (63%)	11,089 (100%)	0 (0%)	0 (0%)	0 (0%)
Non-Hispanic Black	4,481 (7.9%)	4,481 (19%)	0 (0%)	0 (0%)	0 (0%)	0 (0%)
Hispanic/Latino	9,423 (17%)	3,177 (14%)	0 (0%)	4,038 (100%)	2,208 (100%)	0 (0%)
Other (Asian)	17,358 (30%)	914 (3.9%)	0 (0%)	0 (0%)	0 (0%)	16,444 (100%)
**Education (%)**						
Primary or less	28,243 (50%)	4,414 (19%)	3,661 (36%)	3,574 (89%)	2,009 (91%)	14,585 (89%)
Secondary	20,317 (36%)	13,637 (59%)	4,889 (48%)	113 (2.8%)	99 (4.5%)	1,579 (9.6%)
Tertiary	7,619 (14%)	5,246 (23%)	1,656 (16%)	345 (8.6%)	94 (4.3%)	278 (1.7%)
Missing	901	4	883	6	6	2
Anthropometric measurements						
**Weight (kg)**	71 (59, 84)	81 (69, 94)	76 (66, 87)	68 (59, 78)	63 (54, 72)	58 (51, 66)
**Height (cm)**	162 (155, 170)	166 (159, 173)	165 (159, 173)	156 (150, 164)	155 (148, 162)	158 (152, 164)
**Waist (cm)**	94 (85, 104)	100 (91, 110)	96 (87, 105)	98 (90, 105)	93 (86, 101)	86 (79, 93)
**BMI (kg/m^2)**	26.6 (23.3, 30.6)	29.0 (25.5, 33.3)	27.3 (24.7, 30.8)	27.6 (24.6, 30.8)	26.1 (23.2, 29.3)	23.3 (20.9, 25.9)
**WHtR**	0.58 (0.53, 0.64)	0.60 (0.55, 0.67)	0.57 (0.53, 0.63)	0.62 (0.57, 0.67)	0.60 (0.55, 0.65)	0.54 (0.50, 0.59)
**AnthropoAge (yrs)**	61 (55, 71)	63 (56, 72)	62 (55, 70)	63 (56, 71)	76 (69, 84)	57 (51, 65)
**AnthropoAge-Accel (yrs)**	-0.4 (-2.1, 1.4)	-0.7 (-2.7, 1.7)	-0.5 (-2.1, 1.3)	-0.4 (-2.2, 1.7)	-0.6 (-2.4, 1.6)	-0.2 (-1.3, 1.1)
**Accelerated aging (%)**	24,447 (43%)	9,612 (41%)	4,640 (42%)	1,751 (43%)	949 (43%)	7,495 (46%)

Attribute	Overall<br>N = 57,080	HRS<br>N = 23,301	ELSA<br>N = 11,089	MHAS<br>N = 4,038	CRELES<br>N = 2,208	CHARLS<br>N = 16,444
Health behaviours						
**Smoking (%)**						
Never smoker	27,434 (50%)	10,088 (44%)	4,202 (38%)	2,330 (58%)	1,270 (58%)	9,544 (64%)
Former smoker	17,826 (32%)	9,135 (39%)	5,168 (47%)	1,095 (27%)	777 (35%)	1,651 (11%)
<10/day	3,258 (5.9%)	1,289 (5.6%)	756 (6.8%)	425 (11%)	75 (3.4%)	713 (4.8%)
≥10/day	6,785 (12%)	2,659 (11%)	945 (8.5%)	186 (4.6%)	81 (3.7%)	2,914 (20%)
Missing	1,777	130	18	2	5	1,622
**Alcohol intake (%)***						
Never	26,165 (47%)	9,935 (43%)	941 (9.6%)	2,823 (70%)	1,604 (73%)	10,862 (69%)
Less than weekly	9,427 (17%)	4,388 (19%)	2,156 (22%)	594 (15%)	266 (12%)	2,023 (13%)
Less than daily	14,671 (27%)	7,398 (32%)	5,503 (56%)	546 (14%)	294 (13%)	930 (5.9%)
Daily	4,870 (8.8%)	1,549 (6.7%)	1,194 (12%)	62 (1.5%)	39 (1.8%)	2,026 (13%)
Missing	1,947	31	1,295	13	5	603

**For CRELES, frequency of alcohol intake was recorded as: never, on special occasions, occasionally, and daily.*

Attribute	Overall	HRS	ELSA	MHAS	CRELES	CHARLS
Functional assessment						
**ADL deficits**						
0	49,361 (87%)	20,475 (88%)	9,749 (88%)	3,686 (92%)	1,859 (84%)	13,592 (83%)
1	4,303 (7.6%)	1,603 (6.9%)	848 (7.6%)	169 (4.2%)	167 (7.6%)	1,516 (9.3%)
2	1,660 (2.9%)	648 (2.8%)	278 (2.5%)	95 (2.4%)	67 (3.0%)	572 (3.5%)
3	856 (1.5%)	325 (1.4%)	127 (1.1%)	40 (1.0%)	52 (2.4%)	312 (1.9%)
4	455 (0.8%)	153 (0.7%)	61 (0.6%)	18 (0.4%)	38 (1.7%)	185 (1.1%)
5	246 (0.4%)	61 (0.3%)	25 (0.2%)	6 (0.1%)	24 (1.1%)	130 (0.8%)
Missing	199	36	1	24	1	137
**IADL deficits**						
0	47,523 (86%)	19,274 (89%)	10,092 (91%)	3,683 (92%)	1,469 (67%)	13,005 (80%)
1	4,386 (7.9%)	1,505 (6.9%)	647 (5.8%)	200 (5.0%)	251 (11%)	1,783 (11%)
2	1,813 (3.3%)	566 (2.6%)	253 (2.3%)	63 (1.6%)	152 (6.9%)	779 (4.8%)
3	914 (1.7%)	224 (1.0%)	64 (0.6%)	37 (0.9%)	108 (4.9%)	481 (3.0%)
4	619 (1.1%)	118 (0.5%)	32 (0.3%)	21 (0.5%)	227 (10%)	221 (1.4%)
Missing	1,825	1,614	1	34	1	175

Attribute	Overall N = 57,080	HRS N = 23,301	ELSA N = 11,089	MHAS N = 4,038	CRELES N = 2,208	CHARLS N = 16,444
Health status and comorbidities						
**Self-reported health**						
**1**	4,373 (8.1%)	2,451 (11%)	1,582 (14%)	90 (2.2%)	124 (5.6%)	126 (0.9%)
**2**	11,753 (22%)	6,860 (29%)	3,285 (30%)	174 (4.3%)	216 (9.8%)	1,218 (9.1%)
**3**	14,781 (27%)	7,383 (32%)	3,463 (31%)	1,204 (30%)	685 (31%)	2,046 (15%)
**4**	16,688 (31%)	4,973 (21%)	2,017 (18%)	1,979 (49%)	979 (44%)	6,740 (50%)
**5**	6,342 (12%)	1,621 (7.0%)	737 (6.6%)	562 (14%)	199 (9.0%)	3,223 (24%)
**Missing**	3,143	13	5	29	5	3,091
**Diabetes (%)**	7,904 (14%)	4,737 (20%)	795 (7.2%)	750 (19%)	501 (23%)	1,121 (7.3%)
Missing	1,164	0	0	74	6	1,084
**Arterial hypertension (%)**	24,070 (43%)	12,586 (54%)	4,081 (37%)	1,712 (43%)	1,267 (58%)	4,424 (29%)
Missing	1,067	0	0	70	6	991
**Myocardial infarction (%)**	8,737 (16%)	4,595 (20%)	1,727 (16%)	159 (4.0%)	129 (5.9%)	2,127 (14%)
Missing	1,099	0	0	65	5	1,029
**Cancer (%)**	4,006 (7.2%)	2,808 (12%)	788 (7.1%)	97 (2.4%)	144 (6.6%)	169 (1.1%)
Missing	1,096	0	1	62	13	1,020
**Chronic lung disease (%)**	5,041 (9.0%)	1,975 (8.5%)	609 (5.5%)	274 (6.9%)	417 (19%)	1,766 (11%)
Missing	1,085	0	1	63	14	1,007
**Stroke (%)**	2,530 (4.5%)	1,517 (6.5%)	382 (3.4%)	88 (2.2%)	104 (4.7%)	439 (2.8%)
Missing	1,058	0	0	65	6	987
**Arthritis (%)**	22,237 (40%)	11,899 (51%)	3,544 (32%)	891 (22%)	375 (17%)	5,528 (36%)
Missing	1,060	0	1	69	25	965
**No. comorbidities**						
0	16,202 (29%)	4,575 (20%)	3,979 (36%)	1,493 (38%)	441 (21%)	5,714 (38%)
1	17,895 (32%)	6,539 (28%)	3,783 (34%)	1,368 (35%)	855 (40%)	5,350 (35%)
≥2	21,433 (39%)	12,187 (52%)	3,326 (30%)	1,067 (27%)	840 (39%)	4,013 (27%)
Missing	1,550	0	1	110	72	1,367
Mortality data						
**No. of deaths (%)**	11,098 (19%)	6,164 (26%)	1,120 (10%)	1,423 (35%)	297 (13%)	2,094 (13%)
**Follow-up (yrs)**	7.5 (3.9, 9.1)	8.2 (4.0, 12.1)	4.2 (2.2, 7.8)	9.2 (8.0, 18.7)	3.4 (1.8, 3.8)	7.1 (5.0, 9.0)

Supplementary Table 2. Comparison of baseline characteristics between anthropometry subset (at least 1 recorded measurement), complete data subset and study population subset (aged 50-94 years, anthropometry within ranges) for **HRS**. Continuous variables are presented as median (IQR), and categorical as n (%). P-values computed with Kruskal-Walli's rank sum test or Pearson's Chi-squared test, where appropriate.

**HRS Attribute**	**Anthropometry subset** N = 24,338 (100%)	**With complete data** N = 23,417 (96.2%)	**Study population** N = 23,301 (95.7%)	**p-value**
**Age (years)**	61 (55, 71)	62 (56, 72)	62 (56, 71)	0.2
Missing	0	0	0	
**Women (%)**	13,823 (57%)	13,292 (57%)	13,215 (57%)	>0.9
Missing	0	0	0	
**Race/ethnicity (%)**				>0.9
Non-Hispanic White	15,349 (63%)	14,787 (63%)	14,729 (63%)	
Non-Hispanic Black	4,742 (19%)	4,505 (19%)	4,481 (19%)	
Hispanic/Latino	3,288 (14%)	3,196 (14%)	3,177 (14%)	
Other	947 (3.9%)	918 (3.9%)	914 (3.9%)	
Missing	12	11	0	
**Primary or less education (%)**	4,625 (19%)	4,445 (19%)	4,414 (19%)	>0.9
Missing	4	4	4	
**BMI (kg/m^2)**	29 (25, 33)	29 (25, 33)	29 (25, 33)	0.4
Missing	1,261	0	0	
**WHtR**	0.60 (0.55, 0.67)	0.60 (0.55, 0.67)	0.60 (0.55, 0.67)	0.030
Missing	658	0	0	
**AnthropoAge (years)**	63 (56, 72)	63 (56, 72)	63 (56, 72)	0.4
Missing	1,430	11	0	
**Current smoker (%)**	4,228 (17%)	4,093 (17%)	4,078 (18%)	>0.9
Missing	133	130	130	
**Current alcohol intake (%)**	13,849 (57%)	13,391 (57%)	13,335 (57%)	0.7
Missing	36	31	31	
**≥1 ADL deficit (%)**	3,113 (13%)	2,828 (12%)	2,790 (12%)	0.012

**HRS Attribute**	**Anthropometry subset** N = 24,338 (100%)	**With complete data** N = 23,417 (96.2%)	**Study population** N = 23,301 (95.7%)	**p-value**
Missing	42	39	36	
**≥1 IADL deficit (%)**	2,668 (12%)	2,437 (11%)	2,413 (11%)	0.052
Missing	1,703	1,631	1,614	
**Self-reported health**	3.00 (2.00, 4.00)	3.00 (2.00, 4.00)	3.00 (2.00, 4.00)	0.15
Missing	15	13	13	
**Diabetes mellitus (%)**	5,051 (21%)	4,759 (20%)	4,737 (20%)	0.4
Missing	0	0	0	
**Arterial hypertension (%)**	13,228 (54%)	12,649 (54%)	12,586 (54%)	0.7
Missing	0	0	0	
**Myocardial infarction (%)**	4,826 (20%)	4,623 (20%)	4,595 (20%)	>0.9
Missing	0	0	0	
**Cancer (%)**	2,906 (12%)	2,827 (12%)	2,808 (12%)	0.9
Missing	0	0	0	
**Chronic lung disease (%)**	2,092 (8.6%)	1,985 (8.5%)	1,975 (8.5%)	0.9
Missing	0	0	0	
**Stroke (%)**	1,626 (6.7%)	1,527 (6.5%)	1,517 (6.5%)	0.7
Missing	0	0	0	
**Arthritis (%)**	12,457 (51%)	11,956 (51%)	11,899 (51%)	>0.9
Missing	0	0	0	
**Multimorbidity (%)**	12,811 (53%)	12,248 (52%)	12,187 (52%)	0.7
Missing	0	0	0	
**Number of deaths (%)**	6,578 (27%)	6,225 (27%)	6,164 (26%)	0.3
Missing	0	0	0	
**Follow-up time (years)**	8.3 (4.0, 12.1)	8.2 (4.0, 12.0)	8.2 (4.0, 12.1)	>0.9
Missing	0	0	0	

Supplementary Table 3. Comparison of baseline characteristics between anthropometry subset (at least 1 recorded measurement), complete data subset and study population subset (aged 50-94 years, anthropometry within ranges) for **ELSA**. Continuous variables are presented as median (IQR), and categorical as n (%). P-values computed with Kruskal-Walli's rank sum test or Pearson's Chi-squared test, where appropriate.

ELSA Attribute	Anthropometry subset N = 11,414 (100%)	With complete data N = 11,096 (97.2%)	Study population N = 11,089 (97.2%)	p-value
**Age (years)**	62 (56, 71)	62 (56, 71)	62 (56, 71)	0.6
Missing	0	0	0	
**Women (%)**	6,251 (55%)	6,081 (55%)	6,079 (55%)	>0.9
Missing	0	0	0	
**Primary or less education (%)**	3,821 (36%)	3,661 (36%)	3,661 (36%)	0.7
Missing	915	885	883	
**BMI (kg/m^2)**	27.3 (24.7, 30.8)	27.3 (24.7, 30.8)	27.3 (24.7, 30.8)	>0.9
Missing	423	0	0	
**WHtR**	0.57 (0.52, 0.63)	0.57 (0.53, 0.63)	0.57 (0.53, 0.63)	0.7
Missing	448	0	0	
**AnthropoAge (years)**	61 (55, 70)	62 (55, 70)	62 (55, 70)	0.4
Missing	555	0	0	
**Current smoker (%)**	1,781 (16%)	1,720 (16%)	1,719 (16%)	>0.9
Missing	1,369	1,295	1,295	
**Current alcohol intake (%)**	9,050 (90%)	8,858 (90%)	8,853 (90%)	0.7
Missing	1,369	1,295	1,295	
**≥1 ADL deficit (%)**	1,463 (13%)	1,341 (12%)	1,339 (12%)	0.2
Missing	1	1	1	
**≥1 IADL deficit (%)**	1,113 (9.8%)	998 (9.0%)	996 (9.0%)	0.073
Missing	1	1	1	
**Self-reported health**	3.00 (2.00, 4.00)	3.00 (2.00, 3.00)	3.00 (2.00, 3.00)	0.5
Missing	6	5	5	
**Diabetes mellitus (%)**	817 (7.2%)	795 (7.2%)	795 (7.2%)	>0.9

**ELSA Attribute**	**Anthropometry subset** N = 11,414 (100%)	**With complete data** N = 11,096 (97.2%)	**Study population** N = 11,089 (97.2%)	**p-value**
Missing	0	0	0	
**Arterial hypertension (%)**	4,214 (37%)	4,087 (37%)	4,081 (37%)	>0.9
Missing	0	0	0	
**Myocardial infarction (%)**	1,808 (16%)	1,727 (16%)	1,727 (16%)	0.8
Missing	0	0	0	
**Cancer (%)**	804 (7.0%)	788 (7.1%)	788 (7.1%)	>0.9
Missing	1	1	1	
**Chronic lung disease (%)**	637 (5.6%)	610 (5.5%)	609 (5.5%)	>0.9
Missing	1	1	1	
**Stroke (%)**	407 (3.6%)	382 (3.4%)	382 (3.4%)	0.8
Missing	0	0	0	
**Arthritis (%)**	3,697 (32%)	3,546 (32%)	3,544 (32%)	0.7
Missing	1	1	1	
**Multimorbidity (%)**	3,465 (30%)	3,329 (30%)	3,326 (30%)	0.8
Missing	1	1	1	
**Number of deaths (%)**	1,279 (11%)	1,121 (10%)	1,120 (10%)	0.007
Missing	0	0	0	
**Follow-up time (years)**	4.25 (2.17, 7.75)	4.17 (2.17, 7.75)	4.17 (2.17, 7.75)	0.7
Missing	0	0	0	

Supplementary Table 4. Comparison of baseline characteristics between anthropometry subset (at least 1 recorded measurement), complete data subset and study population subset (aged 50-94 years, anthropometry within ranges) for **MHAS**. Continuous variables are presented as median (IQR), and categorical as n (%). P-values computed with Kruskal-Walli's rank sum test or Pearson's Chi-squared test, where appropriate.

**MHAS Attribute**	**Anthropometry subset** N = 4,241 (100%)	**With complete data** N = 4,197 (99%)	**Study population** N = 4,038 (95.2%)	**p-value**
**Age (years)**	60 (54, 68)	60 (54, 68)	61 (55, 68)	0.017
Missing	0	0	0	
**Women (%)**	2,352 (55%)	2,329 (55%)	2,213 (55%)	0.8
Missing	0	0	0	
**Primary or less education (%)**	3,746 (88%)	3,705 (88%)	3,574 (89%)	>0.9
Missing	6	6	6	
**BMI (kg/m^2)**	27.6 (24.6, 30.8)	27.6 (24.6, 30.8)	27.6 (24.6, 30.8)	>0.9
Missing	37	0	0	
**WHtR**	0.62 (0.57, 0.67)	0.62 (0.57, 0.67)	0.62 (0.57, 0.67)	>0.9
Missing	51	0	0	
**AnthropoAge (years)**	62 (56, 71)	62 (56, 71)	63 (56, 71)	0.023
Missing	60	0	0	
**Current smoker (%)**	637 (15%)	635 (15%)	613 (15%)	>0.9
Missing	3	2	2	
**Current alcohol intake (%)**	1,249 (30%)	1,240 (30%)	1,202 (30%)	>0.9
Missing	14	13	13	
**≥1 ADL deficit (%)**	354 (8.4%)	338 (8.1%)	328 (8.2%)	0.9
Missing	25	25	24	
**≥1 IADL deficit (%)**	346 (8.2%)	328 (7.9%)	321 (8.0%)	0.8
Missing	42	37	34	
**Self-reported health**	4.00 (3.00, 4.00)	4.00 (3.00, 4.00)	4.00 (3.00, 4.00)	>0.9
Missing	36	31	29	
**Diabetes mellitus (%)**	772 (19%)	763 (19%)	750 (19%)	0.9

**MHAS Attribute**	**Anthropometry subset** N = 4,241 (100%)	**With complete data** N = 4,197 (99%)	**Study population** N = 4,038 (95.2%)	**p-value**
Missing	75	75	74	
**Arterial hypertension (%)**	1,767 (42%)	1,750 (42%)	1,712 (43%)	0.7
Missing	72	72	70	
**Myocardial infarction (%)**	161 (3.9%)	162 (3.9%)	159 (4.0%)	>0.9
Missing	66	66	65	
**Cancer (%)**	99 (2.4%)	99 (2.4%)	97 (2.4%)	>0.9
Missing	64	63	62	
**Chronic lung disease (%)**	284 (6.8%)	281 (6.8%)	274 (6.9%)	>0.9
Missing	64	64	63	
**Stroke (%)**	93 (2.2%)	91 (2.2%)	88 (2.2%)	>0.9
Missing	67	66	65	
**Arthritis (%)**	911 (22%)	900 (22%)	891 (22%)	0.7
Missing	70	70	69	
**Multimorbidity (%)**	1,093 (26%)	1,081 (26%)	1,067 (27%)	0.7
Missing	114	112	110	
**Number of deaths (%)**	1,459 (34%)	1,436 (34%)	1,423 (35%)	0.6
Missing	0	0	0	
**Follow-up time (years)**	9.2 (7.9, 18.5)	9.2 (8.1, 18.5)	9.2 (8.0, 18.7)	0.5
Missing	2	0	0	

Supplementary Table 5. Comparison of baseline characteristics between anthropometry subset (at least 1 recorded measurement), complete data subset and study population subset (aged 50-94 years, anthropometry within ranges) for **CRELES**. Continuous variables are presented as median (IQR), and categorical as n (%). P-values computed with Kruskal-Walli's rank sum test or Pearson's Chi-squared test, where appropriate.

**CRELES Attribute**	**Anthropometry subset** N = 2,424 (100%)	**With complete data** N = 2,310 (95.3%)	**Study population** N = 2,208 (91.1%)	**p-value**
**Age (years)**	76 (69, 84)	76 (69, 83)	75 (69, 82)	<0.001
Missing	0	0	0	
**Women (%)**	1,349 (56%)	1,278 (55%)	1,226 (56%)	>0.9
Missing	0	0	0	
**Primary or less education (%)**	2,198 (91%)	2,097 (91%)	2,009 (91%)	>0.9
Missing	18	13	6	
**BMI (kg/m^2)**	25.9 (23.1, 29.2)	25.9 (23.0, 29.2)	26.1 (23.2, 29.3)	0.3
Missing	129	0	0	
**WHtR**	0.60 (0.55, 0.65)	0.60 (0.55, 0.65)	0.60 (0.55, 0.65)	0.7
Missing	135	0	0	
**AnthropoAge (years)**	77 (70, 85)	77 (70, 86)	76 (69, 84)	0.028
Missing	142	0	0	
**Current smoker (%)**	168 (6.9%)	166 (7.2%)	161 (7.3%)	0.9
Missing	8	7	5	
**Current alcohol intake (%)**	638 (26%)	624 (27%)	599 (27%)	0.8
Missing	9	5	5	
**≥1 ADL deficit (%)**	515 (21%)	415 (18%)	348 (16%)	<0.001
Missing	1	1	1	
**≥1 IADL deficit (%)**	936 (39%)	828 (36%)	738 (33%)	0.001
Missing	1	1	1	
**Self-reported health**	4.00 (3.00, 4.00)	4.00 (3.00, 4.00)	4.00 (3.00, 4.00)	0.9
Missing	6	5	5	
**Diabetes mellitus (%)**	538 (22%)	510 (22%)	501 (23%)	0.9

**CRELES Attribute**	**Anthropometry subset** N = 2,424 (100%)	**With complete data** N = 2,310 (95.3%)	**Study population** N = 2,208 (91.1%)	**p-value**
Missing	7	7	6	
**Arterial hypertension (%)**	1,374 (57%)	1,309 (57%)	1,267 (58%)	0.9
Missing	7	7	6	
**Myocardial infarction (%)**	142 (5.9%)	133 (5.8%)	129 (5.9%)	>0.9
Missing	8	8	5	
**Cancer (%)**	168 (7.0%)	155 (6.8%)	144 (6.6%)	0.9
Missing	17	15	13	
**Chronic lung disease (%)**	464 (19%)	440 (19%)	417 (19%)	>0.9
Missing	21	18	14	
**Stroke (%)**	136 (5.6%)	110 (4.8%)	104 (4.7%)	0.3
Missing	7	6	6	
**Arthritis (%)**	431 (18%)	397 (17%)	375 (17%)	0.8
Missing	28	27	25	
**Multimorbidity (%)**	930 (40%)	870 (39%)	840 (39%)	0.9
Missing	88	81	72	
**Number of deaths (%)**	425 (18%)	360 (16%)	297 (13%)	<0.001
Missing	0	0	0	
**Follow-up time (years)**	3.42 (1.75, 3.83)	3.42 (1.75, 3.83)	3.42 (1.75, 3.83)	0.7
Missing	0	0	0	

Supplementary Table 6. Comparison of baseline characteristics between anthropometry subset (at least 1 recorded measurement), complete data subset and study population subset (aged 50-94 years, anthropometry within ranges) for **CHARLS**. Continuous variables are presented as median (IQR), and categorical as n (%). P-values computed with Kruskal-Walli's rank sum test or Pearson's Chi-squared test, where appropriate.

**CHARLS Attribute**	**Anthropometry subset** N = 20,610 (100%)	**With complete data** N = 20,328 (98.6%)	**Study population** N = 16,444 (79.8%)	**p-value**
**Age (years)**	57 (49, 64)	57 (49, 64)	59 (53, 66)	<0.001
Missing	135	0	0	
**Women (%)**	10,856 (53%)	10,710 (53%)	8,414 (51%)	0.005
Missing	0	0	0	
**Primary or less education (%)**	18,280 (89%)	18,028 (89%)	14,585 (89%)	>0.9
Missing	8	2	2	
**BMI (kg/m^2)**	23.4 (21.0, 26.0)	23.4 (21.0, 26.0)	23.3 (20.9, 25.9)	0.003
Missing	261	0	0	
**WHtR**	0.54 (0.49, 0.59)	0.54 (0.49, 0.59)	0.54 (0.50, 0.59)	<0.001
Missing	233	0	0	
**AnthropoAge (years)**	54 (46, 62)	54 (46, 62)	57 (51, 65)	<0.001
Missing	442	1	0	
**Current smoker (%)**	6,376 (31%)	6,311 (31%)	5,249 (32%)	0.092
Missing	1,694	1,674	1,622	
**Current alcohol intake (%)**	6,334 (32%)	6,284 (32%)	4,979 (31%)	0.4
Missing	761	746	603	
**≥1 ADL deficit (%)**	3,030 (15%)	2,901 (15%)	2,715 (17%)	<0.001
Missing	504	499	137	
**≥1 IADL deficit (%)**	3,810 (19%)	3,660 (18%)	3,264 (20%)	<0.001
Missing	190	191	175	
**Self-reported health**	4.00 (3.00, 4.00)	4.00 (3.00, 4.00)	4.00 (3.00, 4.00)	<0.001
Missing	4,214	4,111	3,091	
**Diabetes mellitus (%)**	1,174 (6.5%)	1,147 (6.4%)	1,121 (7.3%)	0.002

**CHARLS Attribute**	**Anthropometry subset** N = 20,610 (100%)	**With complete data** N = 20,328 (98.6%)	**Study population** N = 16,444 (79.8%)	**p-value**
Missing	2,450	2,408	1,084	
**Arterial hypertension (%)**	4,790 (26%)	4,690 (26%)	4,424 (29%)	<0.001
Missing	2,345	2,308	991	
**Myocardial infarction (%)**	2,312 (13%)	2,274 (13%)	2,127 (14%)	0.002
Missing	2,376	2,338	1,029	
**Cancer (%)**	184 (1.0%)	181 (1.0%)	169 (1.1%)	0.7
Missing	2,378	2,340	1,020	
**Chronic lung disease (%)**	1,881 (10%)	1,855 (10%)	1,766 (11%)	<0.001
Missing	2,368	2,328	1,007	
**Stroke (%)**	487 (2.7%)	461 (2.6%)	439 (2.8%)	0.3
Missing	2,336	2,300	987	
**Arthritis (%)**	6,116 (33%)	6,038 (33%)	5,528 (36%)	<0.001
Missing	2,304	2,269	965	
**Multimorbidity (%)**	4,283 (24%)	4,187 (24%)	4,013 (27%)	<0.001
Missing	2,740	2,694	1,367	
**Number of deaths (%)**	2,368 (11%)	2,245 (11%)	2,094 (13%)	<0.001
Missing	0	0	0	
**Follow-up time (years)**	8.75 (5.00, 9.00)	8.75 (5.00, 9.00)	7.08 (5.00, 9.00)	<0.001
Missing	0	0	0	

Supplementary Table 7. Comparison of baseline characteristics between participants **without accelerated aging vs. with accelerated aging** (i.e., AnthropoAgeAccel <0 vs. AnthropoAgeAccel ≥0). Continuous variables are presented as median (IQR), and categorical as n (%). P-values computed with Kruskal-Walli's rank sum test or Pearson's Chi-squared test, where appropriate.

Attribute	**Overall** N = 57,080	**Non-accelerated aging (**AnthropoAgeAccel <0) N = 32,633	**Accelerated aging (**AnthropoAgeAccel ≥0) N = 24,447	p-value
**Age (years)**	61 (55, 70)	61 (55, 70)	61 (55, 71)	<0.001
**Women (%)**	31,147 (55%)	17,829 (55%)	13,318 (54%)	0.7
**G2A study (%)**				<0.001
HRS	23,301 (41%)	13,689 (42%)	9,612 (39%)	
ELSA	11,089 (19%)	6,449 (20%)	4,640 (19%)	
MHAS	4,038 (7.1%)	2,287 (7.0%)	1,751 (7.2%)	
CRELES	2,208 (3.9%)	1,259 (3.9%)	949 (3.9%)	
CHARLS	16,444 (29%)	8,949 (27%)	7,495 (31%)	
**Primary or less education (%)**	28,243 (50%)	14,835 (46%)	13,408 (56%)	<0.001
Missing	901	543	358	
**Weight (kg)**	71 (59, 84)	72 (62, 82)	69 (54, 90)	<0.001
**Height (cm)**	162 (155, 170)	163 (157, 171)	161 (153, 168)	<0.001
**Waist (cm)**	94 (85, 104)	93 (85, 100)	100 (85, 113)	<0.001
**BMI (kg/m^2)**	26.6 (23.3, 30.6)	26.7 (24.3, 29.3)	26.3 (21.6, 34.0)	>0.9
**WHtR**	0.58 (0.53, 0.64)	0.57 (0.52, 0.60)	0.63 (0.54, 0.69)	<0.001
**AnthropoAge (years)**	61 (55, 71)	59 (53, 68)	64 (58, 74)	<0.001
**Current smoker (%)**	10,043 (18%)	5,061 (16%)	4,982 (21%)	<0.001
Missing	1,777	867	910	
**Current alcohol intake (%)**	28,968 (53%)	17,458 (55%)	11,510 (49%)	<0.001
Missing	1,947	977	970	
**≥1 ADL deficit (%)**	7,520 (13%)	3,331 (10%)	4,189 (17%)	<0.001
Missing	199	109	90	
**≥1 IADL deficit (%)**	7,732 (14%)	3,439 (11%)	4,293 (18%)	<0.001

Attribute	Overall<br>N = 57,080	Non-accelerated aging<br>(AnthropoAgeAccel <0)<br>N = 32,633	Accelerated aging<br>(AnthropoAgeAccel ≥0)<br>N = 24,447	p-value
Missing	1,825	1,039	786	
**Self-reported health**	3.00 (2.00, 4.00)	3.00 (2.00, 4.00)	4.00 (3.00, 4.00)	<0.001
Missing	3,143	1,649	1,494	
**Diabetes mellitus (%)**	7,904 (14%)	3,778 (12%)	4,126 (17%)	<0.001
Missing	1,164	659	505	
**Arterial hypertension (%)**	24,070 (43%)	13,056 (41%)	11,014 (46%)	<0.001
Missing	1,067	604	463	
**Myocardial infarction (%)**	8,737 (16%)	4,783 (15%)	3,954 (16%)	<0.001
Missing	1,099	624	475	
**Cancer (%)**	4,006 (7.2%)	2,338 (7.3%)	1,668 (7.0%)	0.11
Missing	1,096	626	470	
**Chronic lung disease (%)**	5,041 (9.0%)	2,234 (7.0%)	2,807 (12%)	<0.001
Missing	1,085	619	466	
**Stroke (%)**	2,530 (4.5%)	1,300 (4.1%)	1,230 (5.1%)	<0.001
Missing	1,058	603	455	
**Arthritis (%)**	22,237 (40%)	12,128 (38%)	10,109 (42%)	<0.001
Missing	1,060	608	452	
**Multimorbidity (%)**	21,433 (39%)	11,266 (35%)	10,167 (43%)	<0.001
Missing	1,550	879	671	
**Number of deaths (%)**	11,098 (19%)	5,480 (17%)	5,618 (23%)	<0.001
**Follow-up time (years)**	7.5 (3.9, 9.1)	7.8 (4.0, 9.1)	7.1 (3.8, 9.0)	<0.001

Supplementary Table 8. Comparison of Harrell's c-statistic using z-scores comparing chronological age (CA) alone vs CA + AnthropoAgeAccel for prediction of all-cause mortality using Cox models stratified by sex and race/ethnicity and adjusted for education, smoking, drinking, and comorbidities. Results are presented separately for de overall population, by G2A study, by race/ethnicity, by sex, and by number of comorbidities.

Strata	CA alone c-statistic (95% CI)	CA + AnthropoAgeAccel c-statistic (95% CI)	Difference	P-value
By G2A study				
**Overall**	0.801 (0.794-0.808)	0.805 (0.798-0.811)	0.0037	<0.001
**HRS**	0.815 (0.806-0.824)	0.819 (0.810-0.827)	0.0038	<0.001
**ELSA**	0.831 (0.815-0.846)	0.833 (0.818-0.848)	0.0020	0.032
**MHAS**	0.775 (0.747-0.802)	0.776 (0.748-0.803)	0.0009	0.31
**CRELES**	0.703 (0.659-0.747)	0.716 (0.673-0.759)	0.0128	0.018
**CHARLS**	0.782 (0.767-0.797)	0.785 (0.771-0.800)	0.0033	0.005
By race/ethnicity				
**White**	0.817 (0.809-0.825)	0.821 (0.813-0.829)	0.0037	<0.001
**Black**	0.743 (0.720-0.766)	0.743 (0.720-0.767)	0.0007	0.405
**Hispanic/ Latino**	0.767 (0.743-0.790)	0.769 (0.746-0.793)	0.0029	0.064
**Asian**	0.782 (0.767-0.797)	0.785 (0.771-0.799)	0.0032	0.009
By sex				
**Men**	0.784 (0.774-0.795)	0.788 (0.778-0.798)	0.0036	<0.001
**Women**	0.815 (0.806-0.824)	0.819 (0.810-0.828)	0.0040	<0.001
By number of comorbidities				
**0**	0.782 (0.762-0.801)	0.786 (0.766-0.806)	0.0042	0.036
**1**	0.775 (0.759-0.791)	0.781 (0.766-0.796)	0.0063	<0.001
**≥2**	0.763 (0.752-0.773)	0.766 (0.756-0.777)	0.0033	<0.001

Supplementary Table 9. Uno's c-statistics and hazard ratios (HR) with 95% confidence intervals (CI) after removing 2020 and 2021 from follow-up. Results were obtained from Cox regressions to predict all-cause mortality using AnthropoAgeAccel, models were stratified by sex and race/ethnicity and progressively adjusted for 1) chronological age (CA), 2) education, and lifestyle, and 3) comorbidities (T2D: Type 2 Diabetes. MI: Myocardial Infarction). ***A total of 770 participants who died after 2020 were removed from this sensitivity analysis (483 from HRS, 273 from MHAS, 14 from CHARLS).***

Study	All participants		Removing 2020-2021	
	Uno's c-statistic (95% CI)	AnthropoAgeAccel HR (95% CI)	Uno's c-statistic (95% CI)	AnthropoAgeAccel HR (95% CI)
Model 1: AnthropoAgeAccel + CA (stratified by sex and race/ethnicity)				
**Overall**	0.777 (0.796-0.758)	1.064 (1.057-1.070)	0.778 (0.790-0.767)	1.064 (1.058-1.071)
**HRS**	0.784 (0.804-0.764)	1.065 (1.058-1.073)	0.784 (0.793-0.774)	1.065 (1.058-1.073)
**MHAS**	0.712 (0.738-0.686)	1.033 (1.008-1.058)	0.736 (0.763-0.708)	1.035 (1.008-1.062)
**CHARLS**	0.766 (0.779-0.753)	1.089 (1.067-1.112)	0.766 (0.778-0.753)	1.089 (1.067-1.112
Model 2: + Education level + Smoking status + Alcohol consumption				
**Overall**	0.783 (0.804-0.762)	1.054 (1.047-1.061)	0.789 (0.801-0.776)	1.054 (1.047-1.061)
**HRS**	0.799 (0.822-0.776)	1.057 (1.049-1.064)	0.806 (0.815-0.797)	1.056 (1.048-1.063)
**MHAS**	0.723 (0.748-0.698)	1.030 (1.004-1.056)	0.748 (0.775-0.721)	1.030 (1.002-1.060)
**CHARLS**	0.776 (0.790-0.762)	1.084 (1.059-1.110)	0.776 (0.790-0.762)	1.084 (1.059-1.109)
Model 3: + Hypertension + T2D + MI + Stroke + Cancer + Lung Disease + Arthritis				
**Overall**	0.806 (0.822-0.791)	1.047 (1.039-1.054)	0.803 (0.815-0.791)	1.047 (1.040-1.054)
**HRS**	0.819 (0.834-0.805)	1.046 (1.039-1.054)	0.820 (0.828-0.811)	1.046 (1.038-1.054)
**MHAS**	0.744 (0.768-0.719)	1.032 (1.008-1.057)	0.774 (0.798-0.749)	1.034 (1.008-1.061)
**CHARLS**	0.784 (0.798-0.770)	1.080 (1.054-1.107)	0.784 (0.798-0.770)	1.080 (1.054-1.107)

Supplementary Table 10. Added predictive performance of AnthropoAgeAccel, Body Roundness Index (BRI), Weight-Adjusted Waist Index (WWI), and A Body Shape Index (ABSI) compared to models with chronological age (CA) alone for the prediction of 10-year all-cause mortality. All models are stratified by sex and ethnicity, and sequentially adjusted for 1) CA alone, 2) CA + education + lifestyle (smoking status and alcohol intake), and 3) CA + education + lifestyle + comorbidities (hypertension, diabetes, myocardial infarction, stroke, lung disease, cancer, arthritis). P-values were obtained from tests for two correlated ROC curves with bootstrapping (b=1000) with CA + AnthropoAgeAccel as reference.

**Marker (10-year all-cause mortality)**	**Model1: CA alone**			**Model 2: + education and lifestyle**			**Model 3: + Comorbidities**		
	**HR (95% CI)**	**AUROC (95% CI)**	**ROC test p-value**	**HR (95% CI)**	**AUROC (95% CI)**	**ROC test p-value**	**HR (95% CI)**	**AUROC (95% CI)**	**ROC test p-value**
CA alone	-	0.783 (0.778-0.789)	<2e-16	-	0.801 (0.796-0.806)	<2e-16	-	0.820 (0.815-0.825)	1e-13
CA + AnthropoAge Accel	1.27 (1.24-1.31)	0.792 (0.787-0.798)	Ref.	1.23 (1.20-1.27)	0.807 (0.802-0.812)	Ref.	1.20 (1.17-1.23)	0.824 (0.819-0.829)	Ref.
CA + BRI	1.08 (1.05-1.12)	0.784 (0.779-0.790)	<2e-16	1.07 (1.04-1.11)	0.802 (0.797-0.808)	<2e-16	0.99 (0.96-1.03)	0.820 (0.815-0.825)	6e-13
CA + WWI	1.23 (1.19-1.27)	0.787 (0.782-0.793)	4e-13	1.19 (1.16-1.23)	0.804 (0.799-0.809)	8e-12	1.14 (1.10-1.18)	0.821 (0.816-0.826)	4e-10
CA + ABSI	1.25 (1.22-1.29)	0.791 (0.786-0.797)	0.108	1.21 (1.17-1.25)	0.806 (0.801-0.811)	0.008	1.18 (1.15-1.22)	0.823 (0.819-0.828)	0.467

Supplementary Table 11. Hazard ratios (95% CI) extracted from time varying Cox proportional hazard regression models clustered by ID (which account for repeated measures over time). All-cause mortality risk was compared across AnthropoAgeAccel quartiles and across participants with and without accelerated aging (AnthropoAgeAccel values ≥0). Models were stratified by sex and race/ethnicity and progressively adjusted for 1) chronological age and follow-up time (years from baseline), and 2) level of education, smoking and drinking frequency, and comorbidities (hypertension, diabetes, myocardial infarction, stroke, cancer, lung disease, arthritis). Results are displayed for the overall G2A population and for each individual study separately (for MHAS, we also compare results for the 2001 and 2012 cohorts).

**Study**	**Accelerated aging (AAA>0)**	**AnthropoAgeAccel quartiles**		
		**Q2 vs Q1**	**Q3 vs Q1**	**Q4 vs Q1**
Adjustment 1: chronological age and follow-up time				
**Overall G2A**	***1.46 (1.39-1.53)***	***1.41 (1.31-1.52)***	***1.68 (1.57-1.81)***	***1.86 (1.73-2.00)***
**HRS**	***1.59 (1.50-1.69)***	***1.56 (1.43-1.71)***	***1.93 (1.76-2.10)***	***2.18 (2.00-2.38)***
**ELSA**	***1.39 (1.23-1.57)***	***1.85 (1.52-2.26)***	***1.98 (1.63-2.40)***	***2.20 (1.80-2.68)***
**MHAS** *All*	***1.23 (1.03-1.46)***	0.89 (0.67-1.18)	1.13 (0.86-1.50)	1.10 (0.85-1.42)
**MHAS** *2001*	1.08 (0.69-1.69)	1.38 (0.58-3.31)	1.34 (0.58-3.12)	1.27 (0.58-2.79)
**MHAS** *2012*	***1.59 (1.14-2.22)***	0.67 (0.38-1.17)	1.20 (0.71-2.02)	***1.66 (1.002-2.74)***
**CRELES**	***1.82 (1.36-2.42)***	***2.09 (1.28-3.39)***	***2.54 (1.58-4.09)***	***3.16 (1.97-5.08)***
**CHARLS**	***1.42 (1.29-1.57)***	***1.30 (1.12-1.51)***	***1.54 (1.33-1.79)***	***1.73 (1.49-2.00)***
Adjustment 2: + comorbidities, education, smoking, and drinking				
**Overall G2A**	***1.39 (1.32-1.46)***	***1.41 (1.31-1.53)***	***1.61 (1.49-1.74)***	***1.73 (1.61-1.87)***
**HRS**	***1.46 (1.37-1.55)***	***1.59 (1.45-1.75)***	***1.86 (1.70-2.04)***	***1.97 (1.80-2.15)***
**ELSA**	***1.28 (1.10-1.47)***	***1.67 (1.32-2.11)***	***1.72 (1.37-2.16)***	***1.79 (1.42-2.25)***
**MHAS** *All*	***1.30 (1.10-1.54)***	0.83 (0.63-1.10)	1.15 (0.87-1.51)	1.07 (0.83-1.38)
**MHAS** *2001*	1.12 (0.73-1.72)	1.34 (0.53-3.39)	1.40 (0.64-3.08)	1.20 (0.61-2.39)
**MHAS** *2012*	***1.49 (1.06-2.10)***	0.74 (0.44-1.26)	1.10 (0.65-1.88)	***1.75 (1.12-2.75)***
**CRELES**	***1.73 (1.29-2.32)***	***2.30 (1.41-3.77)***	***2.45 (1.51-3.97)***	***3.12 (1.93-5.07)***
**CHARLS**	***1.35 (1.20-1.52)***	***1.31 (1.11-1.55)***	***1.43 (1.21-1.69)***	***1.67 (1.41-1.97)***

Supplementary Table 12. Weighted mean (with 95% confidence interval) of AnthropoAge and AnthropoAgeAccel across different follow-up periods presented as number of years from baseline in the ***overall population and stratified by study***. The change in the mean with respect to baseline for both metrics is also shown; for AnthropoAgeAccel, increases (age acceleration) are shadowed in red, while decreases (age deceleration) are shadowed in green.

Study	Years from baseline	AnthropoAge (years)		AnthropoAgeAccel (years)	
		Weighted mean (95% CI)	Mean change	Weighted mean (95% CI)	Mean change
Overall	0	62.0 (61.9, 62.1)	0.00	-0.07 (-0.08, -0.05)	0.000
	2	63.9 (63.7, 64.1)	1.90	0.12 (0.08, 0.16)	0.186
	4	66.4 (66.3, 66.5)	4.40	0.02 (-0.00, 0.04)	0.083
	8	70.7 (70.5, 70.9)	8.65	0.04 (-0.00, 0.07)	0.101
	12	74.1 (73.7, 74.5)	12.09	0.15 (0.07, 0.24)	0.220
HRS	0	62.5 (62.3, 62.6)	0.00	-0.12 (-0.15, -0.09)	0.000
	4	66.9 (66.7, 67.1)	4.44	0.01 (-0.03, 0.05)	0.132
	8	70.6 (70.4, 70.9)	8.16	0.07 (0.02, 0.12)	0.190
	12	74.1 (73.7, 74.5)	11.64	0.21 (0.12, 0.29)	0.328
ELSA	0	62.2 (62.0, 62.5)	0.00	0.00 (-0.02, 0.03)	0.000
	4	66.6 (66.3, 66.9)	4.37	0.09 (0.05, 0.12)	0.083
	8	70.7 (70.4, 71.1)	8.50	-0.05 (-0.10, -0.00)	-0.054
MHAS	0	64.6 (64.0, 65.2)	0.00	-0.02 (-0.10, 0.06)	0.000
	2	67.1 (66.4, 67.9)	2.55	0.33 (0.13, 0.53)	0.348
	12	73.8 (71.6, 76.0)	9.22	-0.74 (-1.19, -0.29)	-0.722
CRELES	0	72.4 (71.9, 72.8)	0.00	-0.48 (-0.55, -0.40)	0.000
	2	74.5 (74.0, 75.0)	2.15	0.18 (0.10, 0.26)	0.653
	4	76.3 (75.7, 76.8)	3.93	0.47 (0.37, 0.57)	0.948
CHARLS	0	59.2 (59.0, 59.4)	0.00	0.01 (-0.01, 0.03)	0.000
	2	61.6 (61.4, 61.9)	2.45	0.07 (0.04, 0.10)	0.065
	4	63.8 (63.6, 64.1)	4.64	-0.10 (-0.13, -0.06)	-0.103

Supplementary Table 13. Weighted mean (with 95% confidence interval) of AnthropoAge and AnthropoAgeAccel across different follow-up periods presented as number of years from baseline in the ***overall population and stratified by sex***. The change in the mean with respect to baseline for both metrics is also shown; for AnthropoAgeAccel, increases (age acceleration) are shadowed in red, while decreases (age deceleration) are shadowed in green.

Study	Years from baseline	AnthropoAge (years)		AnthropoAgeAccel (years)	
		Weighted mean (95% CI)	Mean change	Weighted mean (95% CI)	Mean change
Overall	0	62.0 (61.9, 62.1)	0.00	-0.07 (-0.08, -0.05)	0.000
	2	63.9 (63.7, 64.1)	1.90	0.12 (0.08, 0.16)	0.186
	4	66.4 (66.3, 66.5)	4.40	0.02 (-0.00, 0.04)	0.083
	8	70.7 (70.5, 70.9)	8.65	0.04 (-0.00, 0.07)	0.101
	12	74.1 (73.7, 74.5)	12.09	0.15 (0.07, 0.24)	0.220
Women	0	62.9 (62.7, 63.1)	0.00	-0.08 (-0.10, -0.06)	0.000
	2	64.6 (64.3, 64.9)	1.68	0.15 (0.09, 0.21)	0.232
	4	67.3 (67.1, 67.5)	4.40	0.04 (0.01, 0.07)	0.122
	8	71.4 (71.2, 71.7)	8.53	0.01 (-0.04, 0.07)	0.095
	12	75.0 (74.5, 75.5)	12.11	0.12 (-0.01, 0.24)	0.198
Men	0	61.0 (60.9, 61.2)	0.00	-0.05 (-0.07, -0.03)	0.000
	2	63.2 (62.8, 63.5)	2.14	0.09 (0.04, 0.13)	0.134
	4	65.4 (65.2, 65.6)	4.37	-0.01 (-0.04, 0.02)	0.039
	8	69.7 (69.4, 70.0)	8.70	0.06 (0.01, 0.12)	0.111
	12	72.9 (72.4, 73.5)	11.92	0.20 (0.08, 0.32)	0.251

Supplementary Table 14. Trends of AnthropoAge per year with sequential adjustments. β-coefficients were obtained from generalized estimating equation models with a Gaussian variance function. A β-coefficient >1 indicates that the rate of population aging occurs, on average, faster than expected with each year of follow-up. In all studies, this association is attenuated after sequentially adjusting for sex, ethnicity, education, lifestyle, and comorbidities.

Adjustment	AnthropoAge increase per year (β with 95% CI)				
	HRS	ELSA	MHAS	CRELES	CHARLS
None	1.17 (1.16-1.18)	1.12 (1.11-1.13)	1.09 (1.01-1.17)	1.33 (1.27-1.39)	1.02 (1-1.04)
+ Sex, race/ethnicity	1.16 (1.15-1.17)	1.11 (1.10-1.12)	1.08 (1.00-1.16)	1.33 (1.27-1.38)	1.04 (1.02-1.05)
+ Education, smoking, alcohol intake	1.06 (1.05-1.07)	1.21 (1.19-1.23)	1.07 (0.99-1.15)	1.33 (1.27-1.39)	1.03 (1.01-1.05)
+ Comorbidities	1.19 (1.14-1.23)	1.11 (1.08-1.14)	1.00 (0.91-1.09)	1.31 (1.24-1.37)	0.98 (0.96-1.00)

Supplementary Table 15. Association between 1y increases of AnthropoAgeAccel and new onset ADL/IADL deficits, poor SRH, diabetes, hypertension, myocardial infarction (MI), stroke, cancer and chronic lung disease. Model were sequentially adjusted as follows: 1) Crude estimates for AnthropoAgeAccel, 2) adjusted for chronological age (CA), sex and race/ethnicity, 3) additionally adjusted for education level, smoking and drinkin g frequency, and 4) select comorbidities (diseases used for each adjustment are specified within the table). Results were obtained from weighted generalized estimating equation models with a Poisson (ADL/IADL) or binomial (SRH, comorbidities) variance function to compute rate ratios (RR) or odds ratios (OR) with 95% confidence interval, respectively.

**Outcome**	**Estimate for AnthropoAgeAccel (1y increase) *RR with 95% CI †OR with 95% CI**				
	**Crude estimates**	**+ CA, Sex, Race/ Ethnicity**	**+ Education, smoking, drinking**	**+ Comorbidities**	
**New onset ADL deficit***	1.137 (1.111-1.164)	1.124 (1.099-1.150)	1.114 (1.089-1.141)	1.108 (1.084-1.132)	**All** (Diabetes, hypertension, MI, stroke, cancer, lung disease, arthritis)
**New onset IADL deficit***	1.145 (1.119-1.171)	1.139 (1.115-1.163)	1.125 (1.101-1.150)	1.116 (1.093-1.140)	
**New onset poor SRH†**	1.119 (1.090-1.148)	1.137 (1.105-1.170)	1.124 (1.091-1.158)	1.108 (1.075-1.142)	
**New onset diabetes†**	1.053 (1.021-1.086)	1.042 (1.013-1.072)	1.041 (1.013-1.071)	1.028 (1.000-1.056)	**All but diabetes**
**New onset hypertension†**	1.046 (1.023-1.070)	1.031 (1.009-1.053)	1.032 (1.010-1.054)	1.026 (1.005-1.048)	**All but hypertension**
**New onset myocardial infarction (MI) †**	1.027 (1.003-1.052)	1.019 (0.997-1.042)	1.019 (0.997-1.042)	1.006 (0.986-1.028)	**All but MI**
**New onset stroke†**	1.085 (1.028-1.144)	1.072 (1.020-1.127)	1.063 (1.011-1.117)	1.044 (0.994-1.097)	**All but stroke**
**New onset cancer†**	1.089 (1.055-1.124)	1.087 (1.054-1.120)	1.087 (1.055-1.119)	1.074 (1.043-1.105)	**All but cancer**
**New onset chronic lung disease†**	1.145 (1.108-1.184)	1.138 (1.099-1.177)	1.124 (1.087-1.162)	1.113 (1.077-1.150)	**All but lung disease**

SUPPLEMENTARY FIGURES

Supplementary Figure 1. Flowchart of participant selection for HRS detailing the reason and number of participants that were removed at each stage.

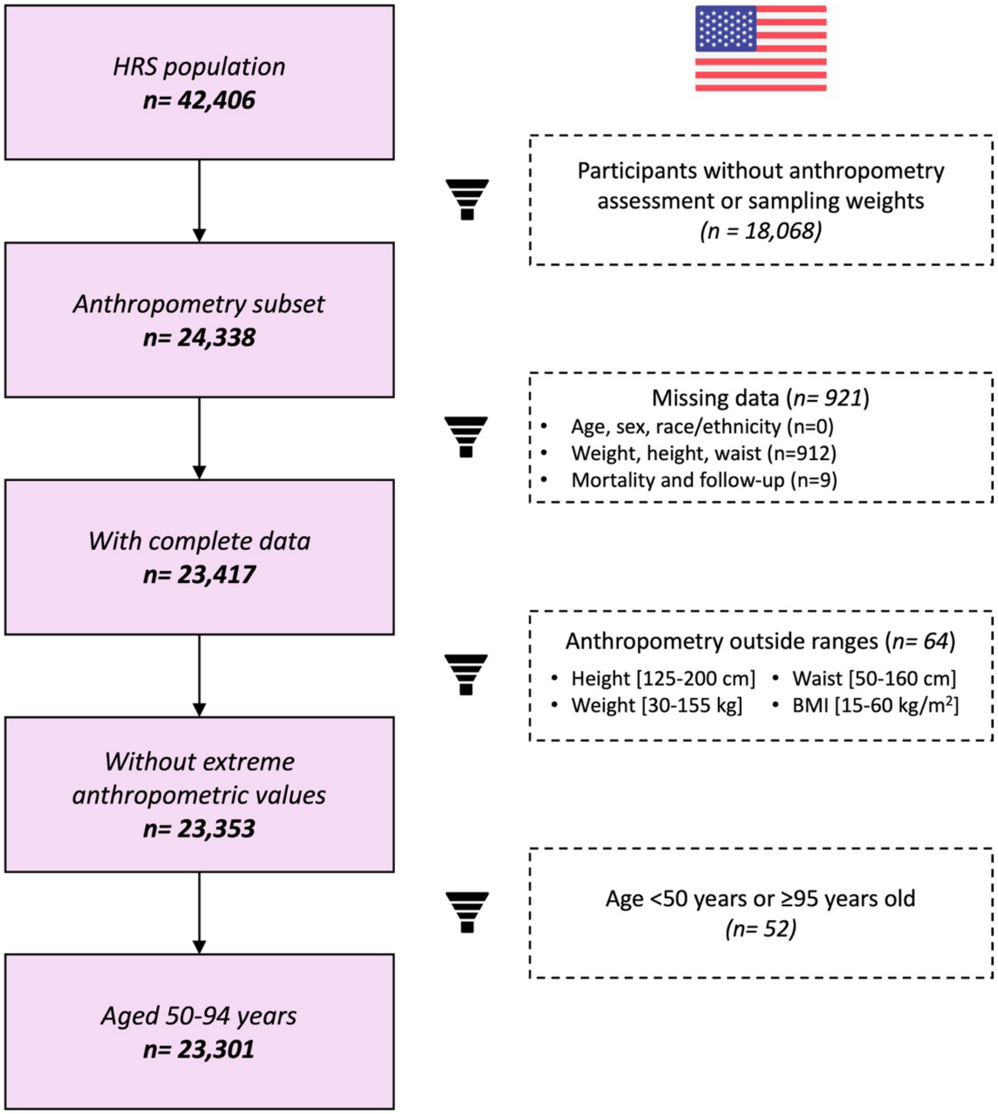

Supplementary Figure 2. Flowchart of participant selection for ELSA detailing the reason and number of participants that were removed at each stage.

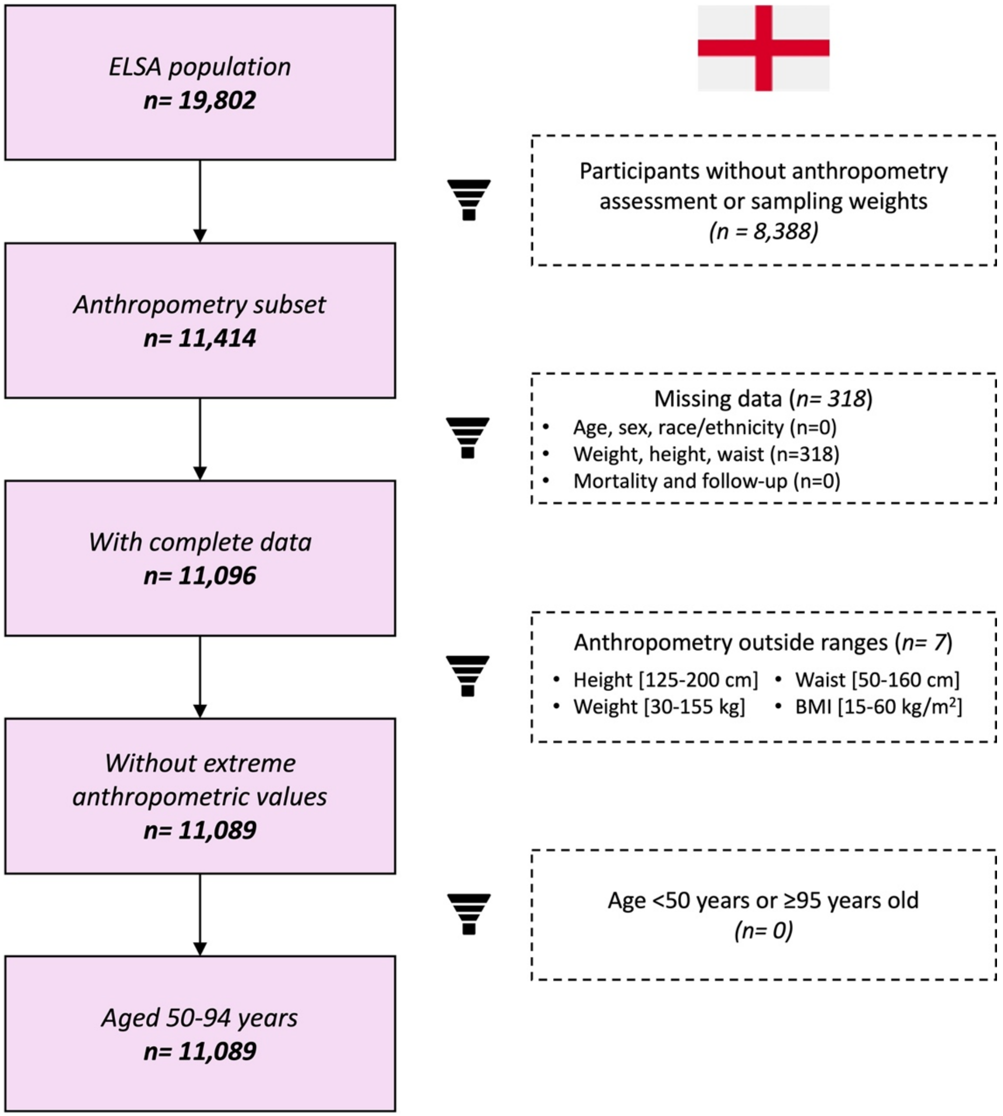

Supplementary Figure 3. Flowchart of participant selection for MHAS detailing the reason and number of participants that were removed at each stage.

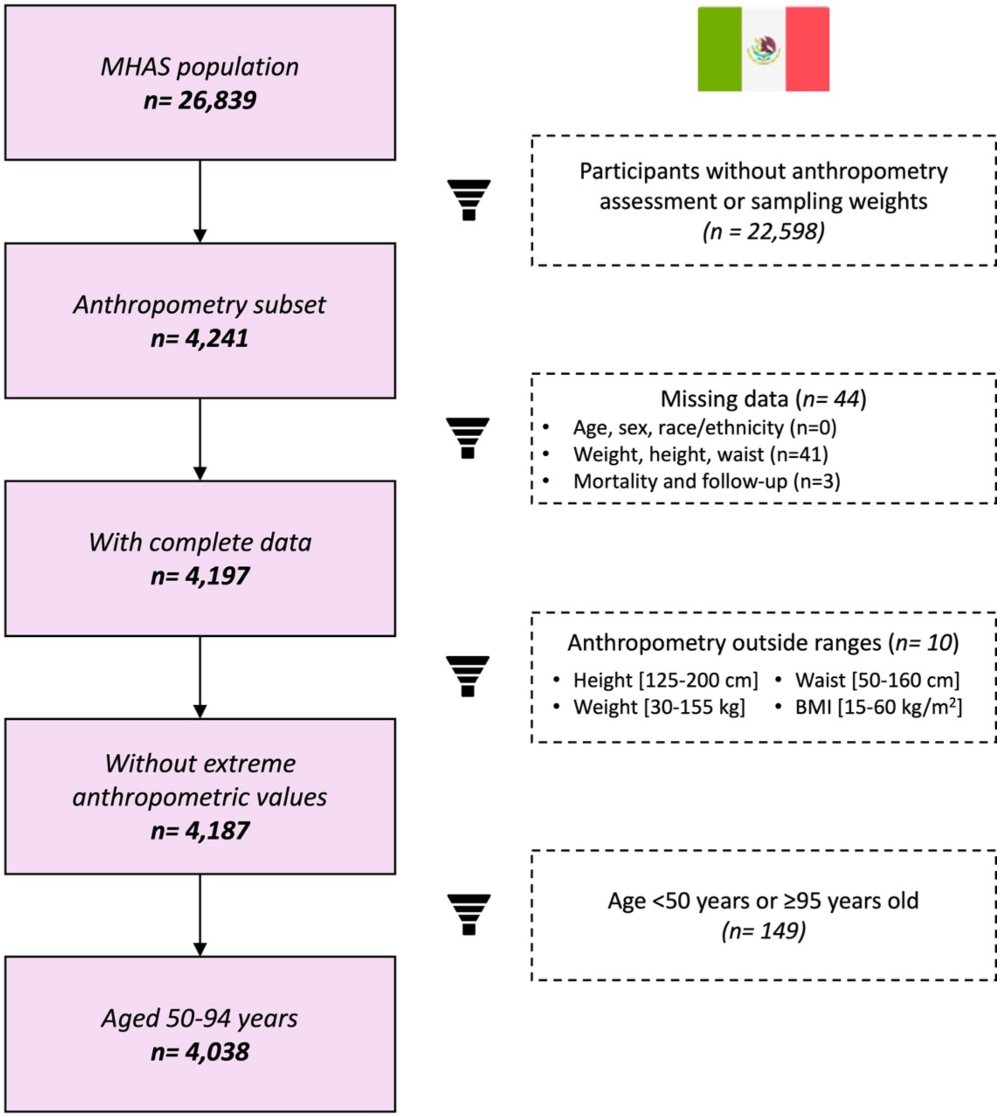

Supplementary Figure 4. Flowchart of participant selection for CRELES detailing the reason and number of participants that were removed at each stage.

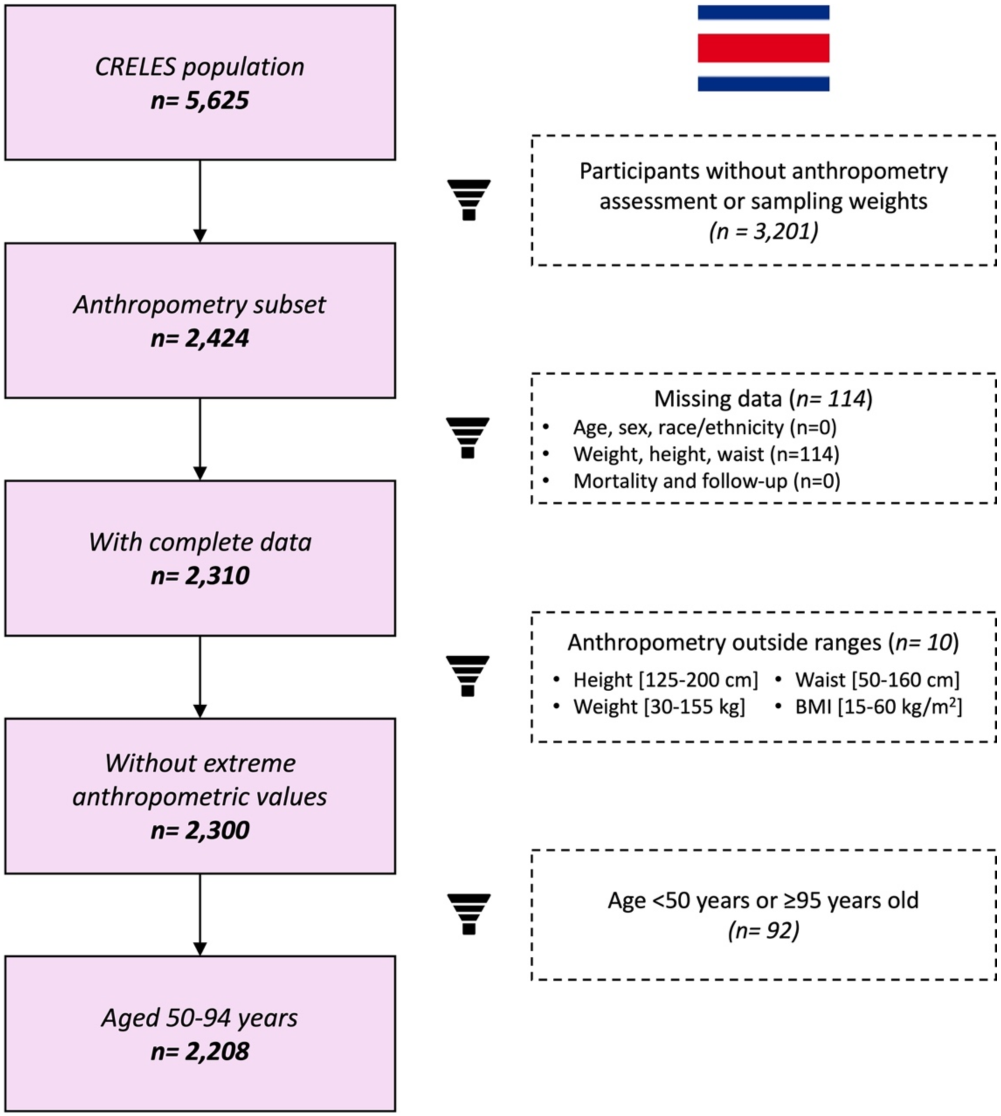

Supplementary Figure 5. Flowchart of participant selection for CHARLS detailing the reason and number of participants that were removed at each stage.

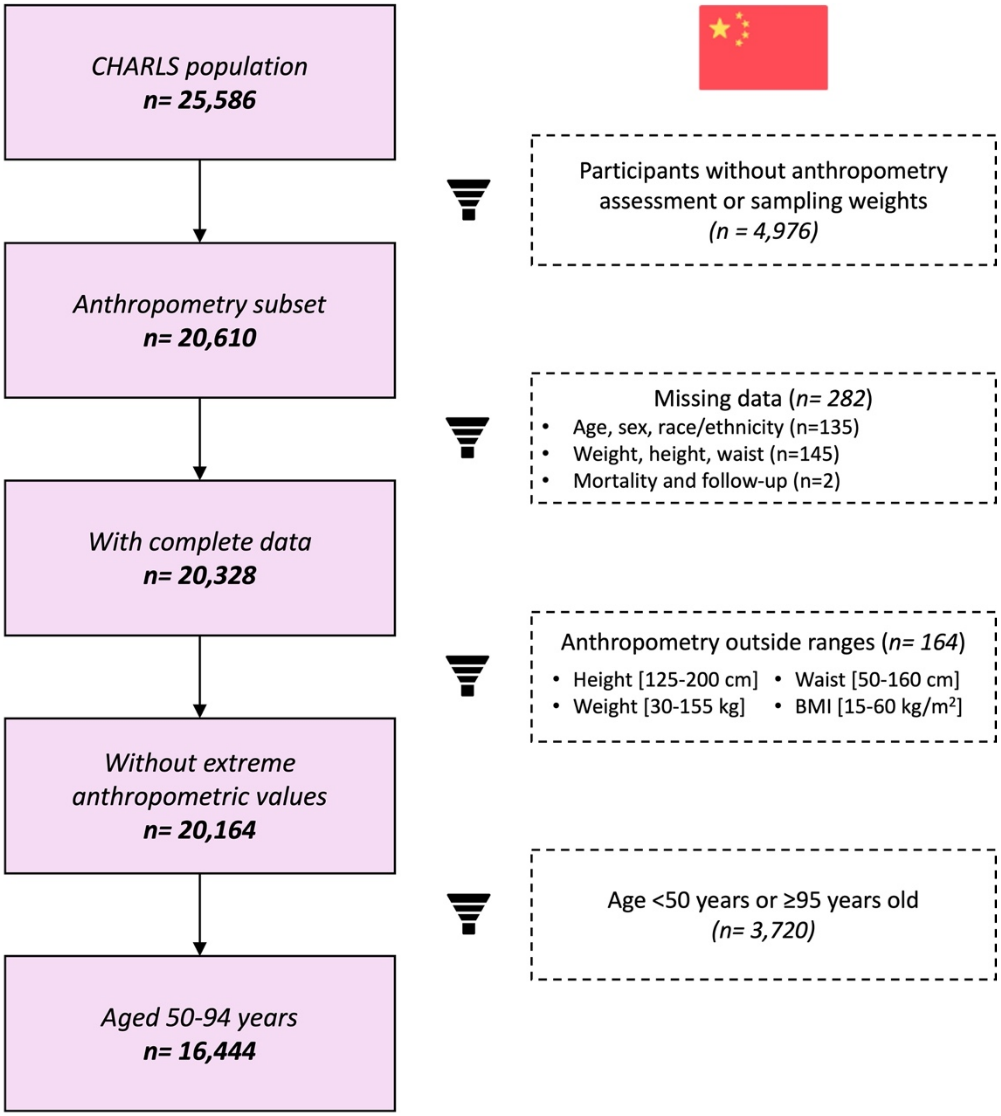

Supplementary Figure 6. Missing data analysis for HRS (before removing missing values of anthropometric and mortality data). We explored whether missingness of anthropometric data (A), sex (B), mortality status (C), and age, weight and waist deciles (D-F) significantly influenced the proportion of missingness for other variables.

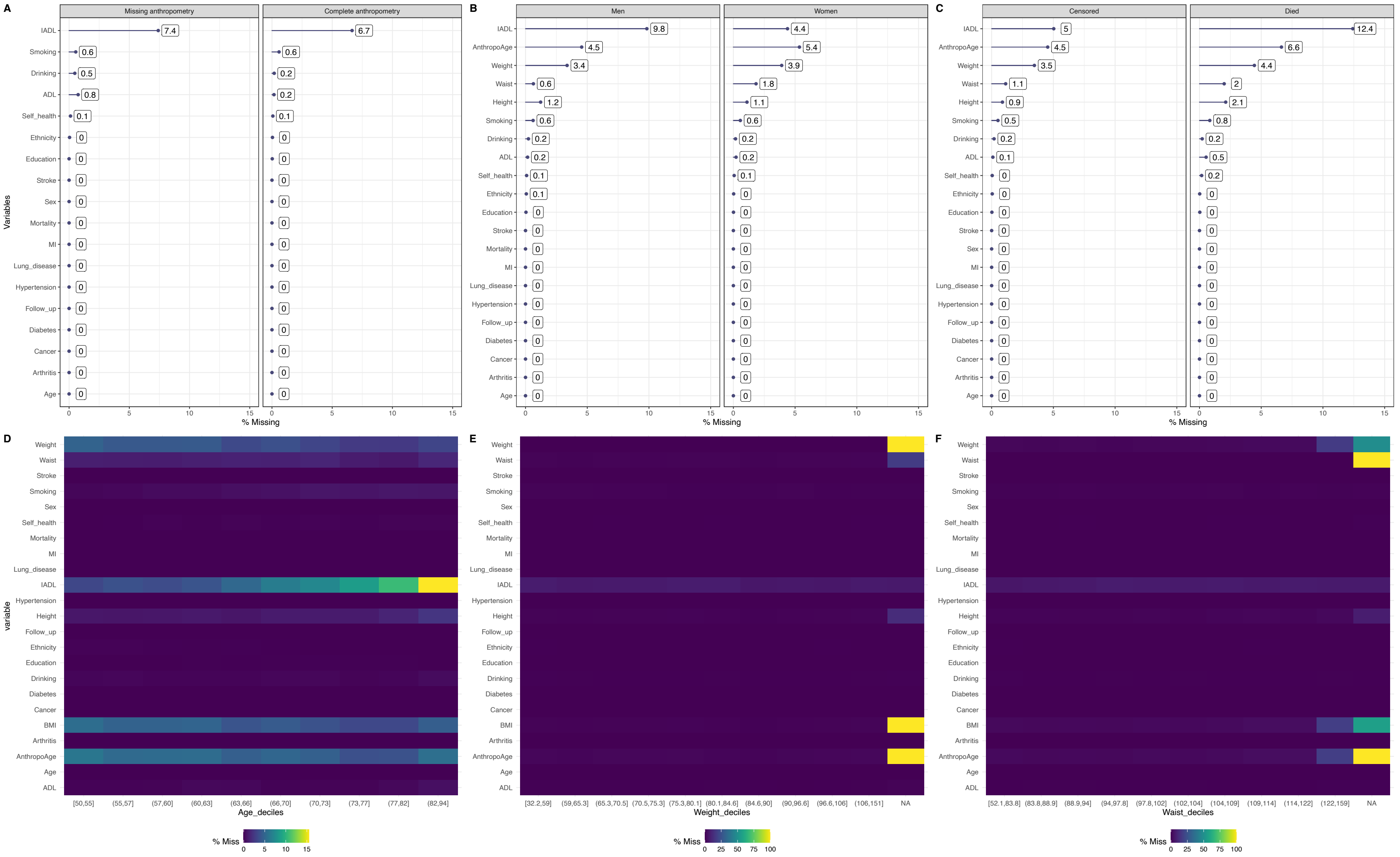

Supplementary Figure 7. Missing data analysis for ELSA (before removing missing values of anthropometric and mortality data). We explored whether missingness of anthropometric data (A), sex (B), mortality status (C), and age, weight and waist deciles (D-F) significantly influenced the proportion of missingness for other variables.

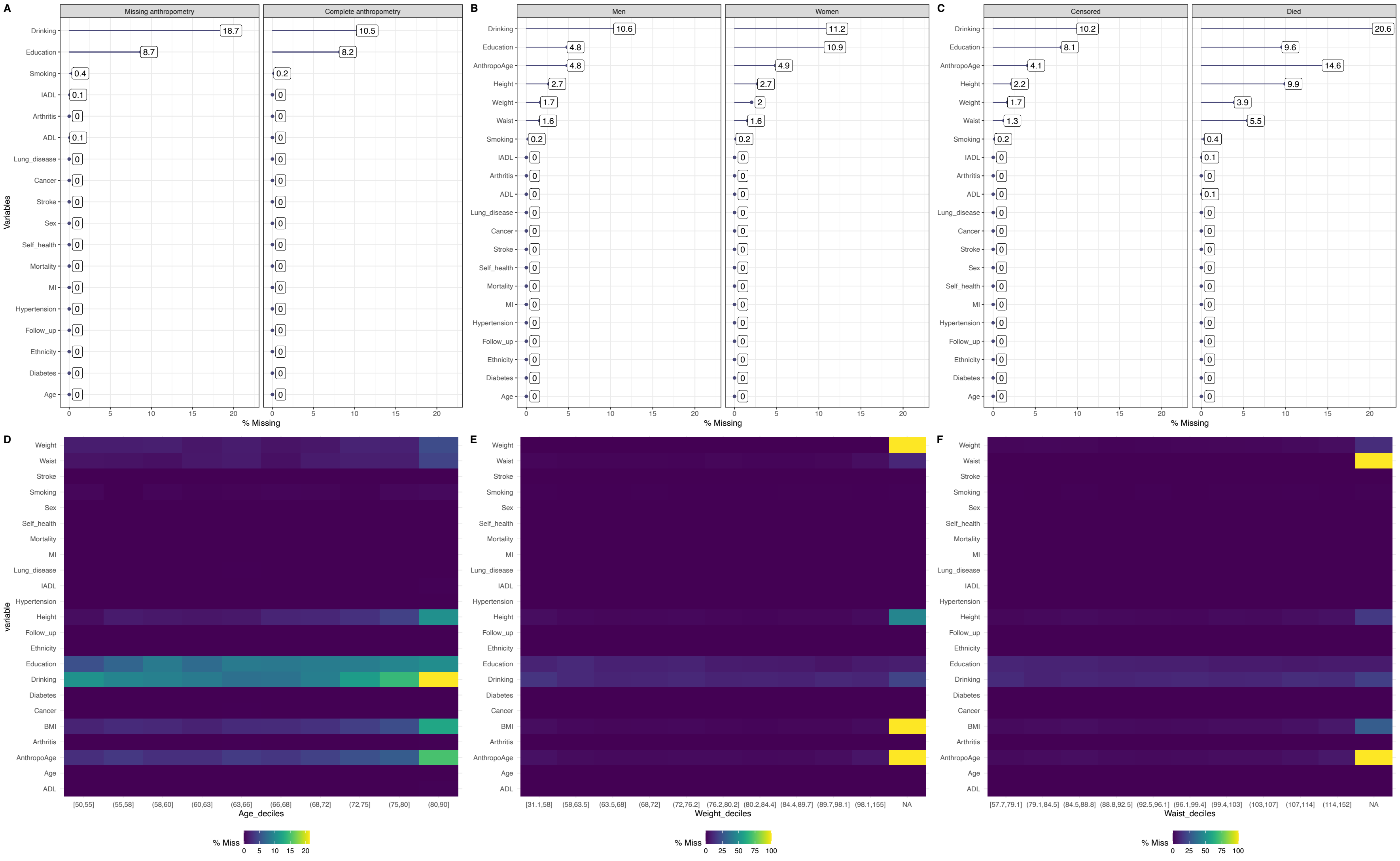

Supplementary Figure 8. Missing data analysis for MHAS (before removing missing values of anthropometric and mortality data). We explored whether missingness of anthropometric data (A), sex (B), mortality status (C), and age, weight and waist deciles (D-F) significantly influenced the proportion of missingness for other variables.

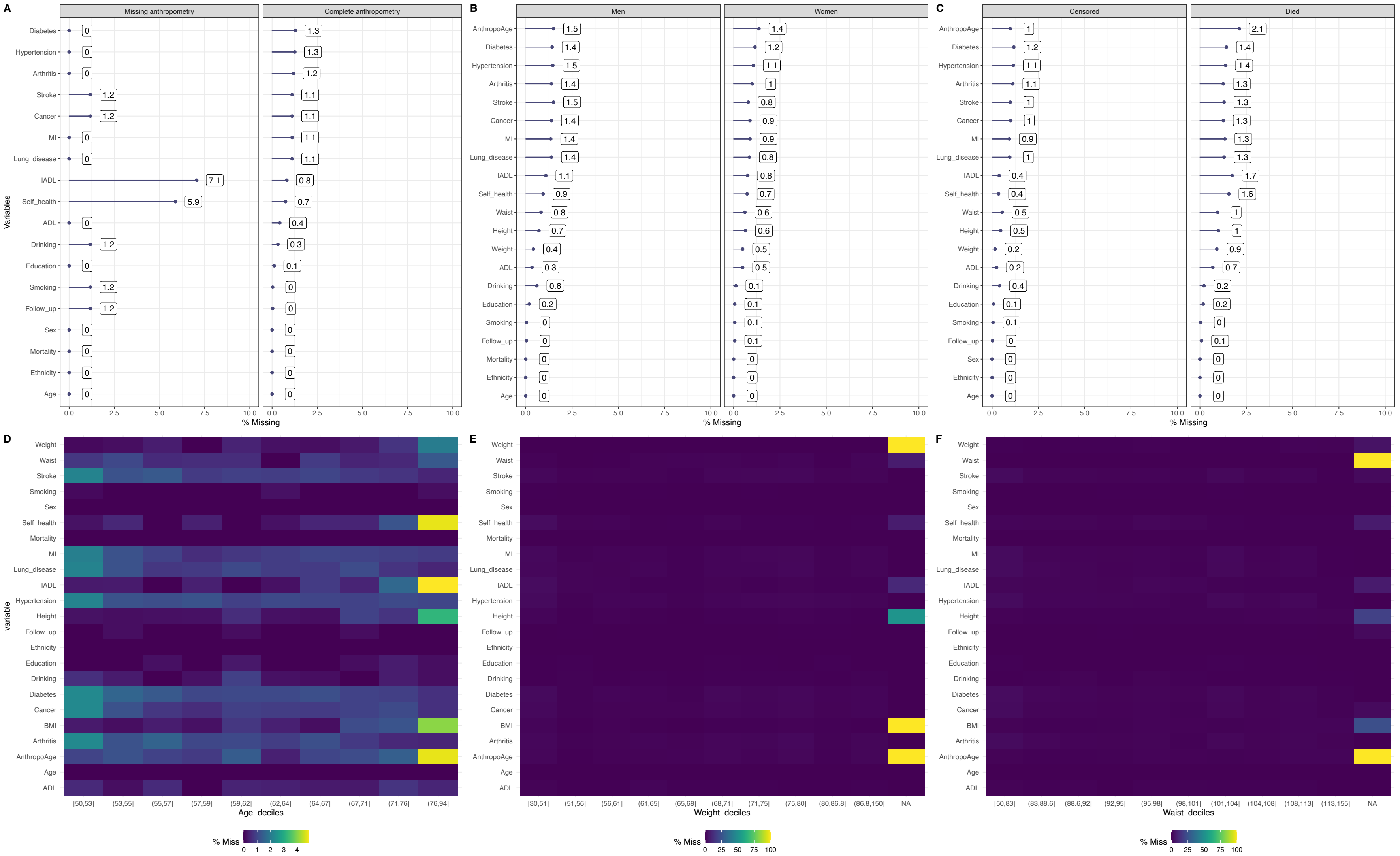

Supplementary Figure 9. Missing data analysis for CRELES (before removing missing values of anthropometric and mortality data). We explored whether missingness of anthropometric data (A), sex (B), mortality status (C), and age, weight and waist deciles (D-F) significantly influenced the proportion of missingness for other variables.

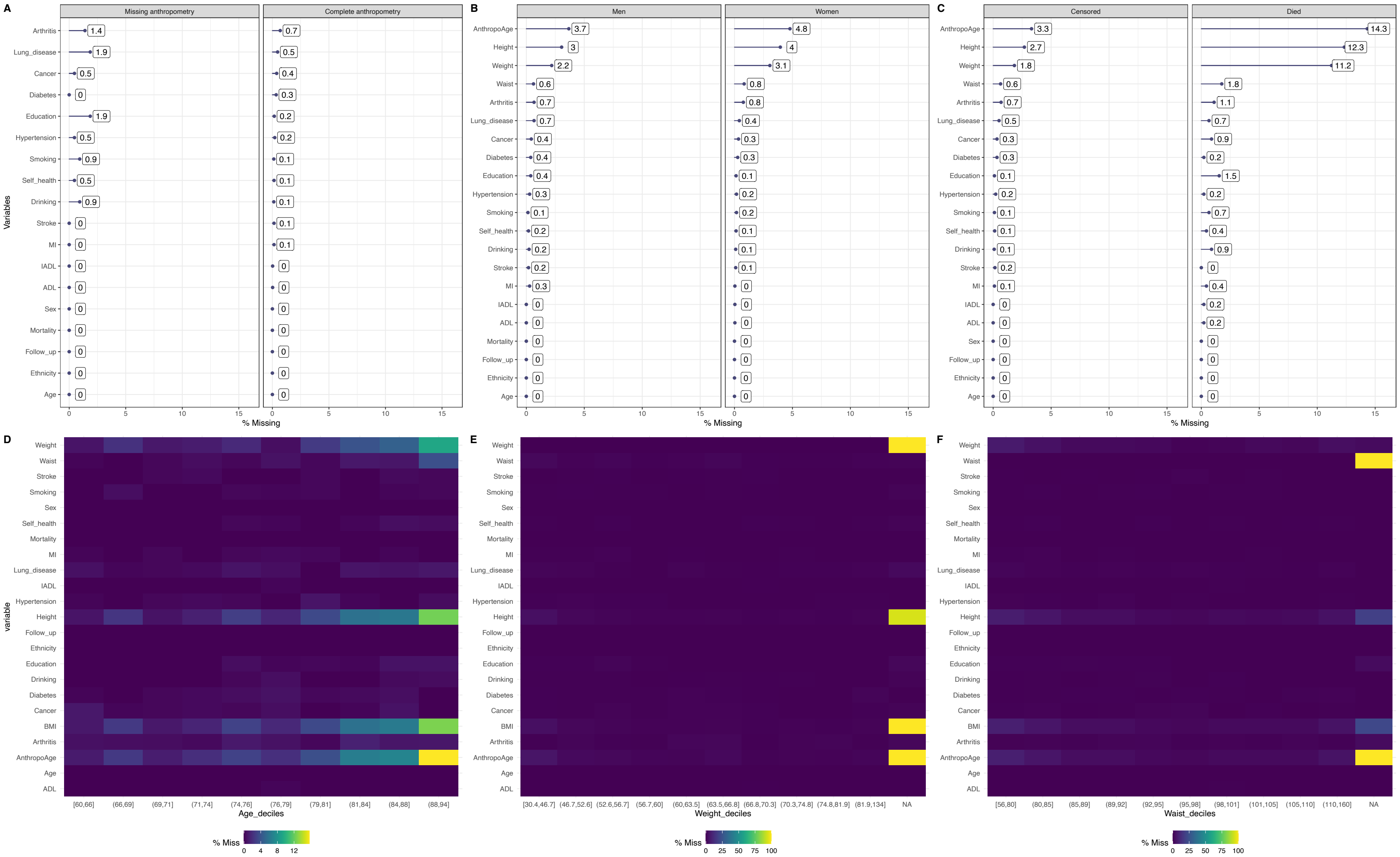

Supplementary Figure 10. Missing data analysis for CHARLS (before removing missing values of anthropometric and mortality data). We explored whether missingness of anthropometric data (A), sex (B), mortality status (C), and age, weight and waist deciles (D-F) significantly influenced the proportion of missingness for other variables.

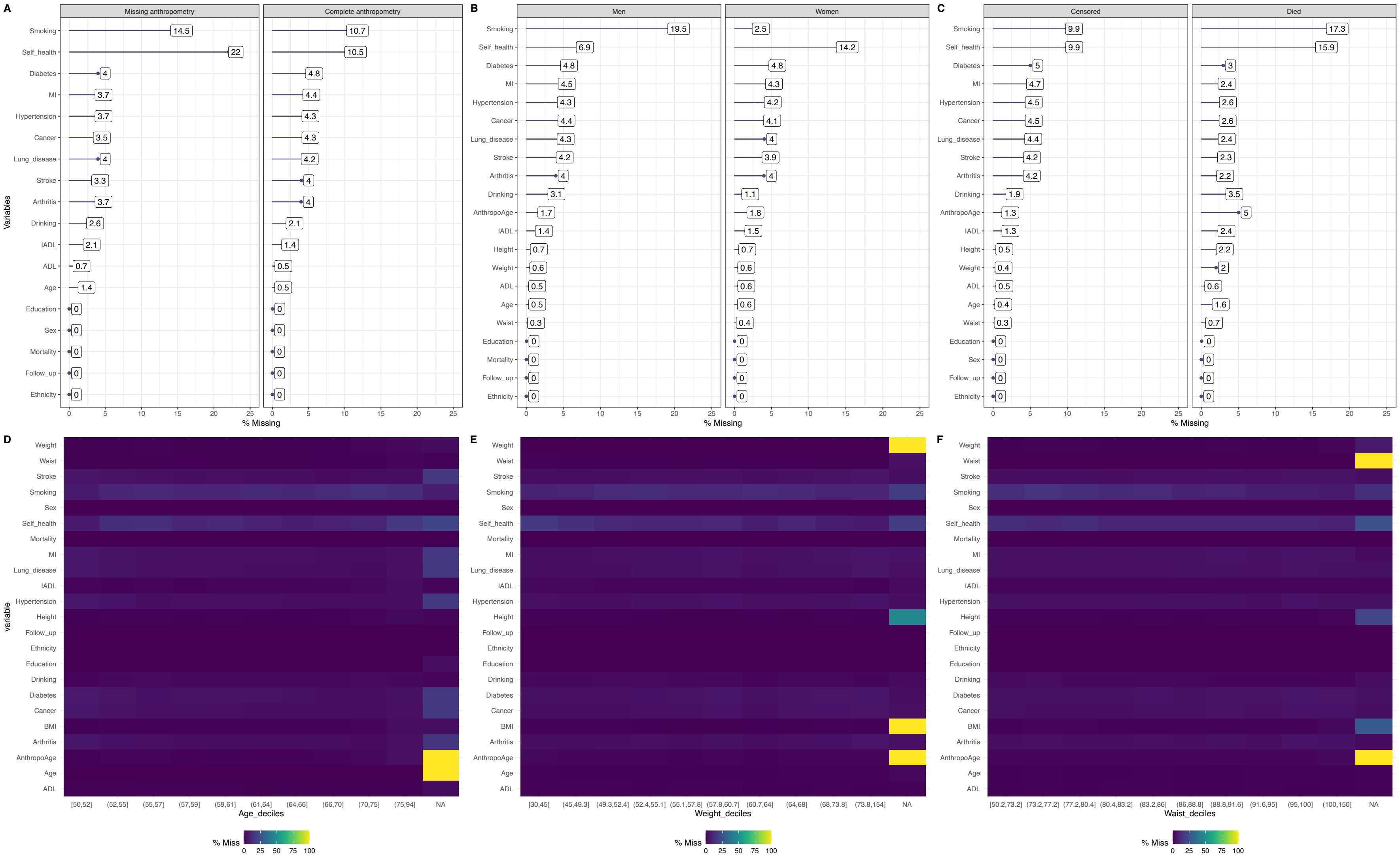

Supplementary Figure 11. Uno's c-statistic for prediction of all-cause mortality using Cox regression models adjusted for sex, race/ethnicity, education level, alcohol consumption, smoking, and comorbidities (baseline model). Each panel compares the prediction of the baseline model alone versus models adding body roundness index (BRI), weight-adjusted waist index (WWI), a body shape index (ABSI), chronological age (CA) alone, and CA + AnthropoAgeAccel. Results are stratified by age quintiles (**A**), race/ethnicity (**B**), number of comorbidities (**C**), sex (**D**), body-mass index (BMI) quintiles (**E**) and waist-to-height ratio (WHtR) quintiles (**F**).

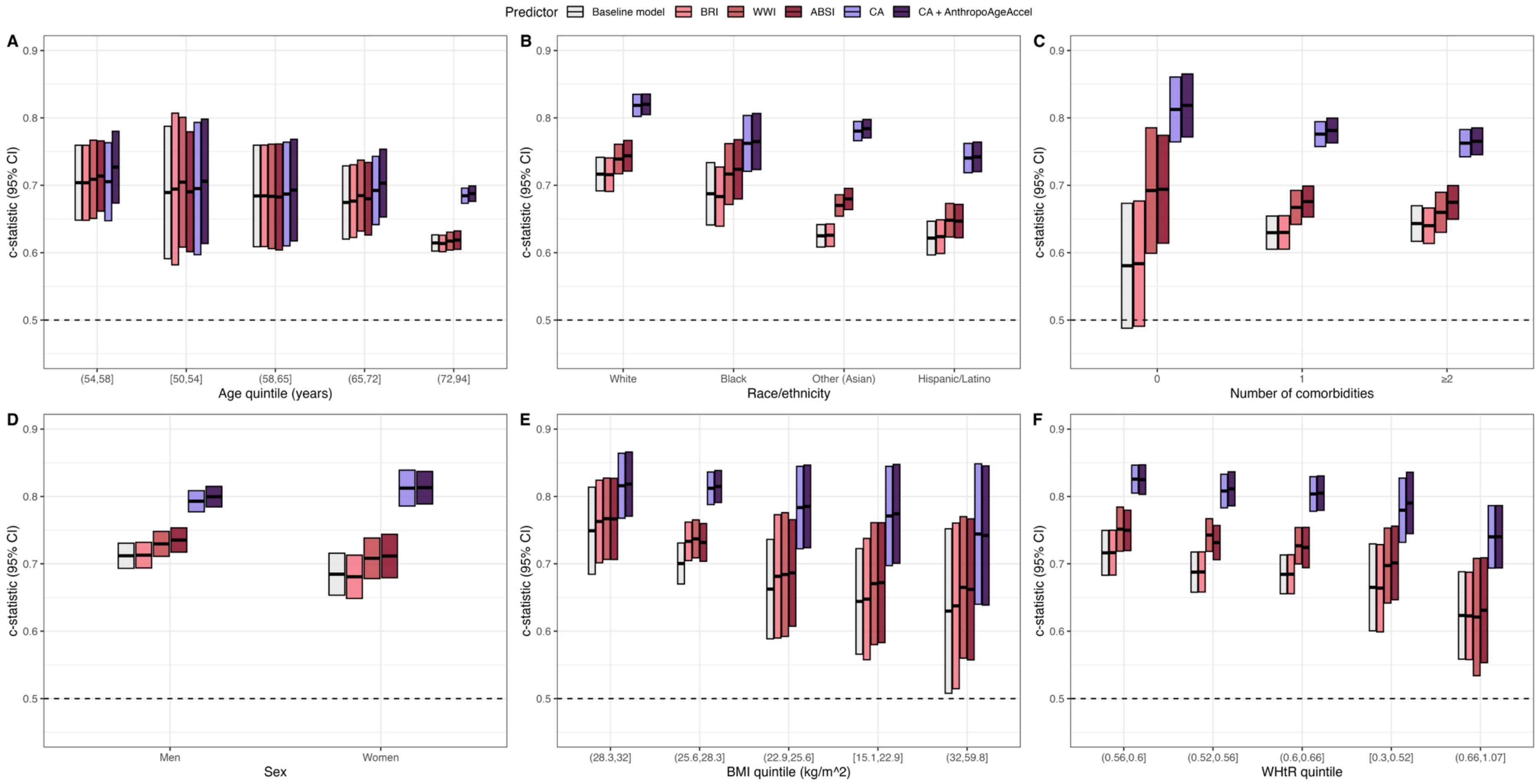

Supplementary Figure 12. Time-dependent area under the receiving operating characteristic curves (tAUC) with inverse probability of censoring weights for prediction of all-cause mortality comparing CA alone vs. CA + AnthropoAgeAccel in MHAS (assessed over 12 years) (**A**), HRS (12 years) (**B**), CHARLS (8 years) (**C**), ELSA (8 years) (**D**), and CRELES (4 years) (**E**); studies are sorted by length of follow-up. The differences in tAUC (delta tAUC, with CA as the reference value) are displayed below (**F-J**). All models are adjusted by sex, race/ethnicity, education level, smoking, alcohol consumption, comorbidities.

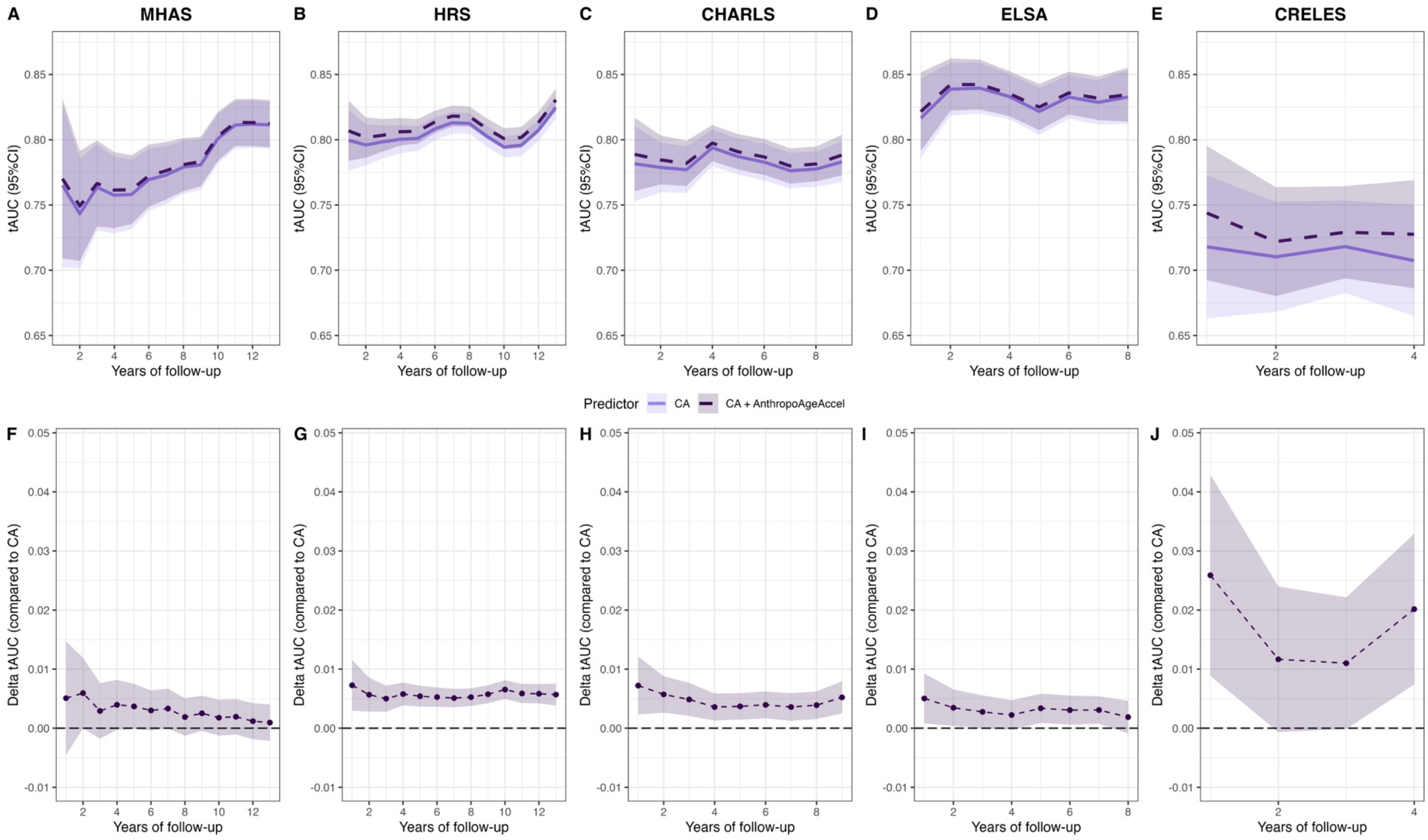

Supplementary Figure 13. The overall MHAS study comprises a first cohort than began follow-up in 2001 and a second cohort that began follow-up in 2012 with differences between how the anthropometric subset was selected (supplementary methods). Here, we compare Uno's c-statistic between the overall MHAS, MHAS 2001, and MHAS 2012 cohorts (**A**); as well as time-dependent area under the receiving operating characteristic curves (tAUC) for MHAS 2001 over 12 years of follow-up (**B**) and MHAS 2012 over 8 years of follow-up (**C**), along with their respective delta tAUC comparing AnthropoAge versus CA (**D-E**).

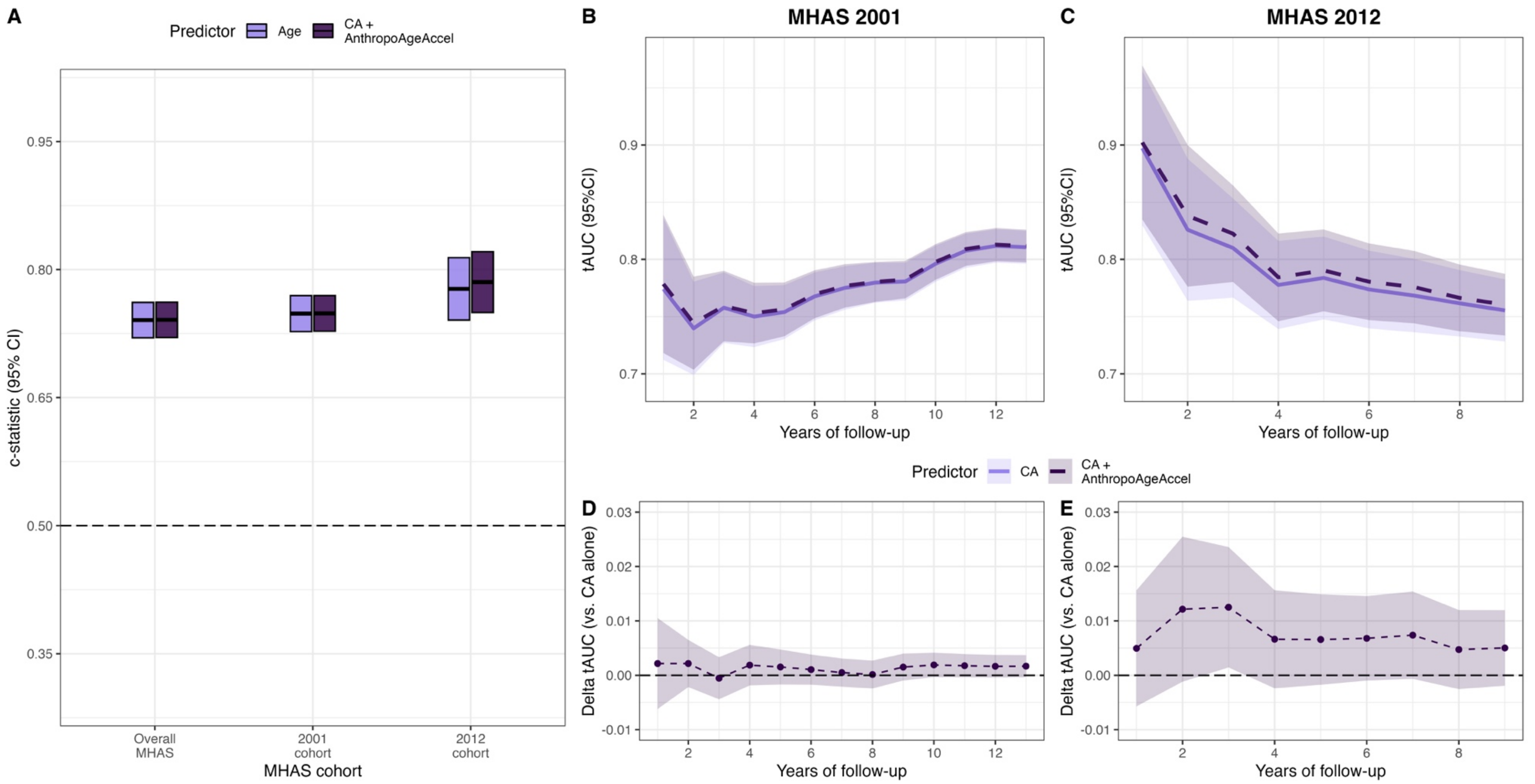

Supplementary Figure 14. Associations of AnthropoAge (**A**), AnthropoAgeAccel (**B**), BMI (**C**), WHtR (**D**), Weight (**E**), Height (**F**), Waist circumference (**G**) and ABSI (**H**) with mortality using restricted cubic splines Cox regression models with 4 knots at the 5th, 35th, 65th, 95th percentiles of each predictor (with respect to the median) using the "*plotRCS*" R package. All models were adjusted for chronological age, sex, race/ethnicity, education level, smoking status, alcohol consumption and comorbidities. Solid blue lines represent hazard ratios (HR), while the shaded areas indicate the 95% confidence intervals (CI). Vertical dotted lines are the median value of each variable, and horizontal dotted lines represent the null hazard ratio (HR = 1).

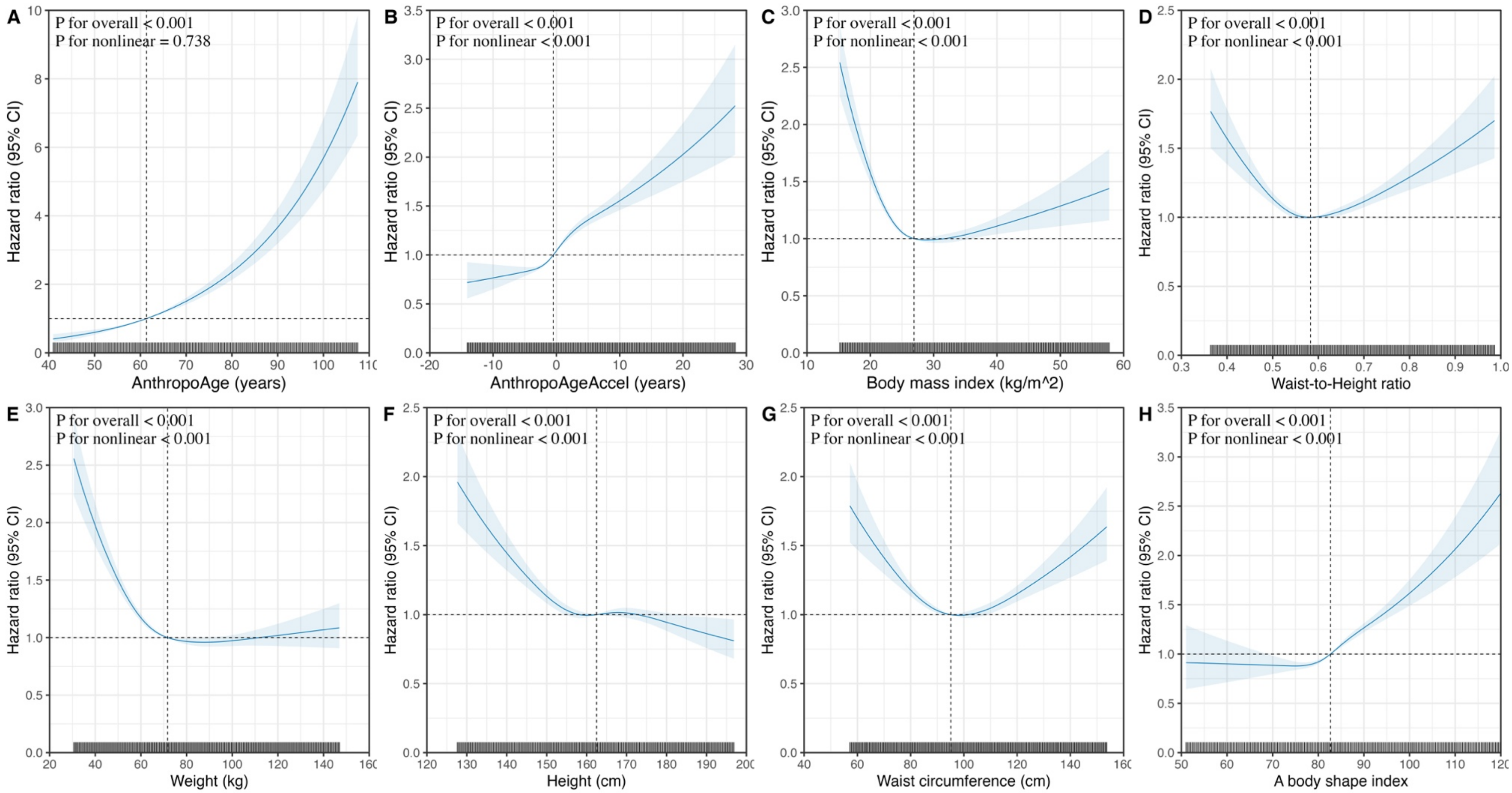

Supplementary figure 15. Kaplan-Meier curves comparing cumulative mortality risk of individuals with and without accelerated aging (AnthropoAgeAccel ≥0) and with or without of multimorbidity (≥2 comorbidities) for each study separately. Studies were arranged by follow-up time: 12 years for MHAS (**A**) and HRS (**B**), 8 years for CHARLS (**C**) and ELSA (**D**), and 4 years for CRELES (**E**). Shown p-values are from log-rank tests for comparison of survival curves.

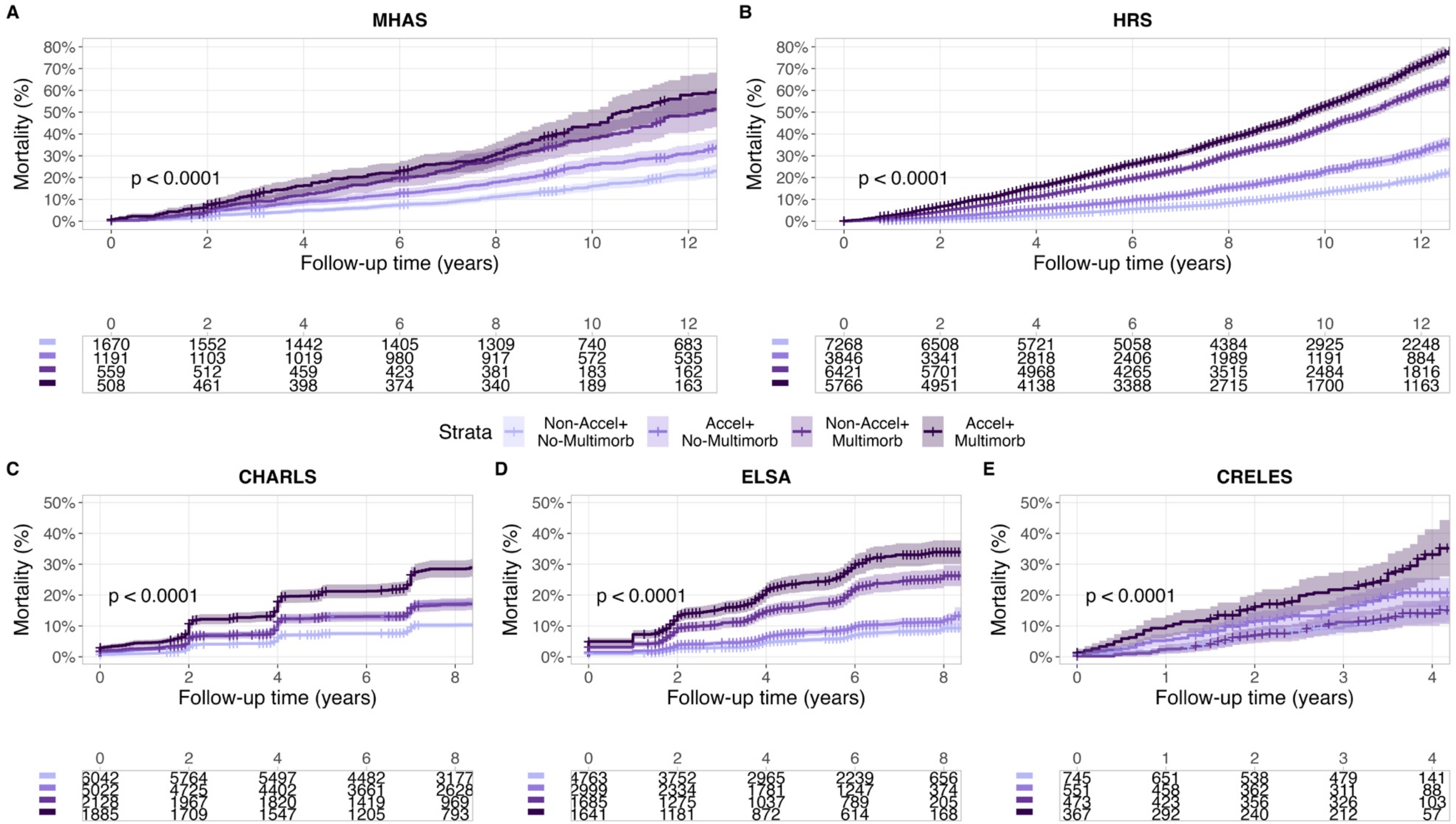

Supplementary Figure 16. Adjusted hazard ratios (95% CI) of all-cause mortality predicted by accelerated aging (AnthropoAgeAccel >0), obtained from Cox models stratified by sex and race/ethnicity, and adjusted for chronological age, education level, smoking and drinking frequency and comorbidities. Estimates were obtained separately for participants with and without multimorbidity (≥2 comorbidities). Larger top numbers represent HR, and the smaller numbers represent the number of deaths in each group.

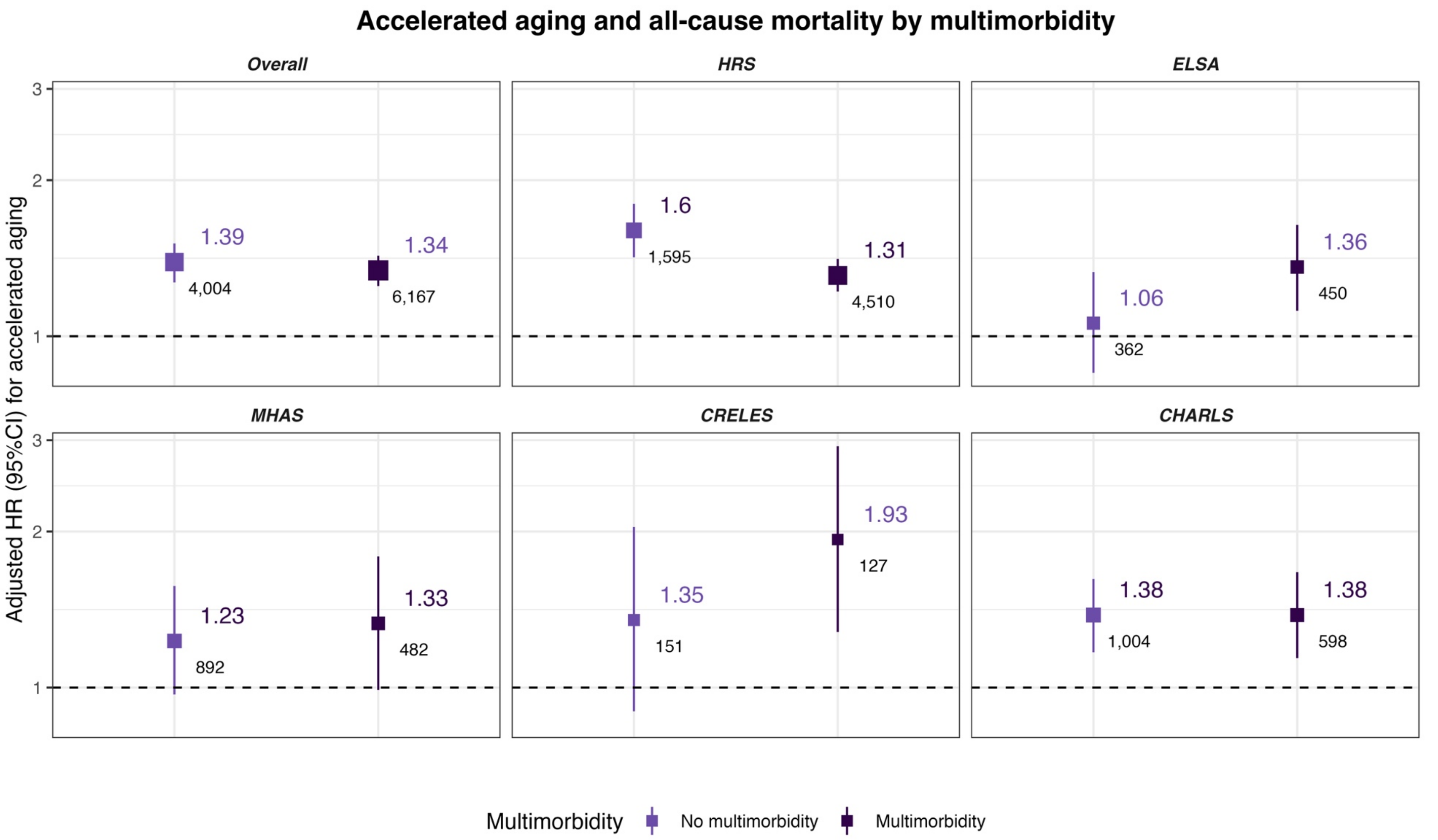

Supplementary Figure 17. Trends of mean chronological age compared to mean AnthropoAge over follow-up time (years from baseline) for each G2A study, ***stratified by sex***. A β-coefficient >1 indicates that the rate of population aging occurs, on average, faster than expected with each year of follow-up; β-coefficients were derived from GEE models for individual countries. Means with 95% confidence intervals were calculated using survey weights.

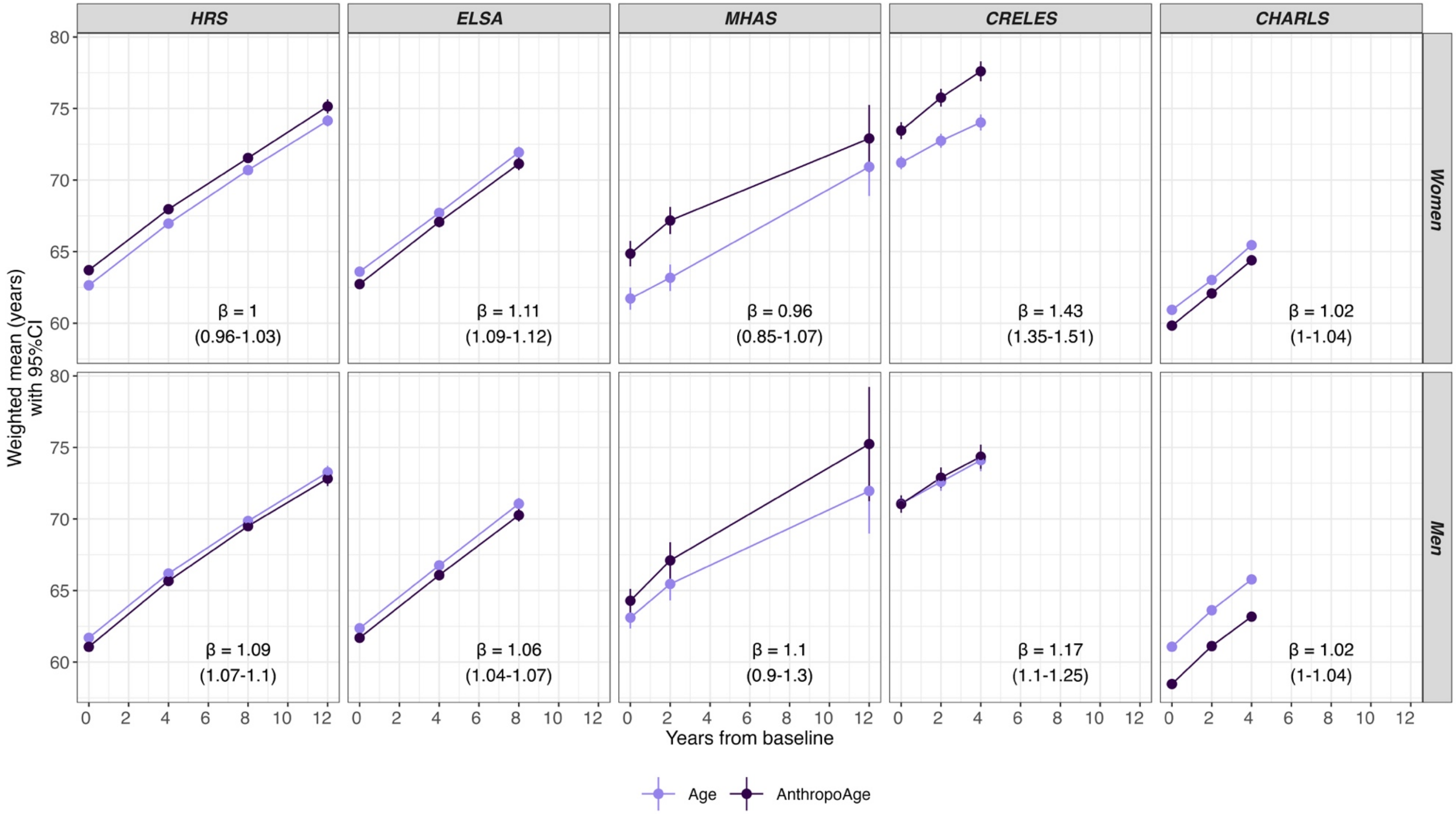

Supplementary Figure 18. Bar graphs showing weighted prevalence (with 95% confidence intervals) of accelerated aging, defined as AnthropoAgeAccel ≥0, over follow-up time (years from baseline), for each G2A study, ***stratified by sex***.

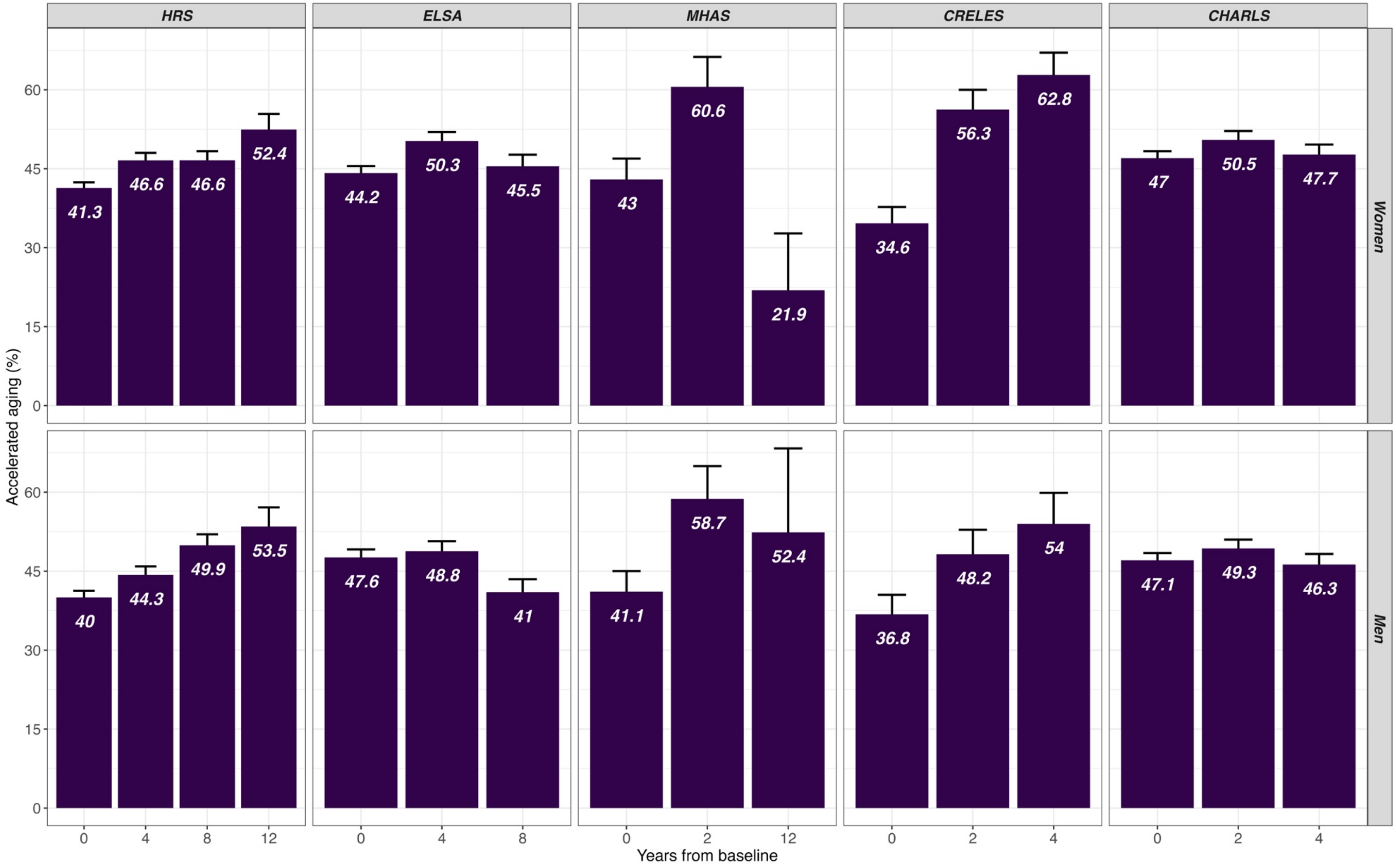

Supplementary Figure 19. Trends of mean chronological age compared to mean AnthropoAge over follow-up time (years from baseline) for each G2A study. AnthropoAge estimates are stratified by the upper vs. lower quartiles of age acceleration: ***AnthropoAgeAccel first quartile (acceleration Q1) vs AnthropoAgeAccel fourth quartile (acceleration Q4)***. Means with 95% confidence intervals were calculated using survey weights.

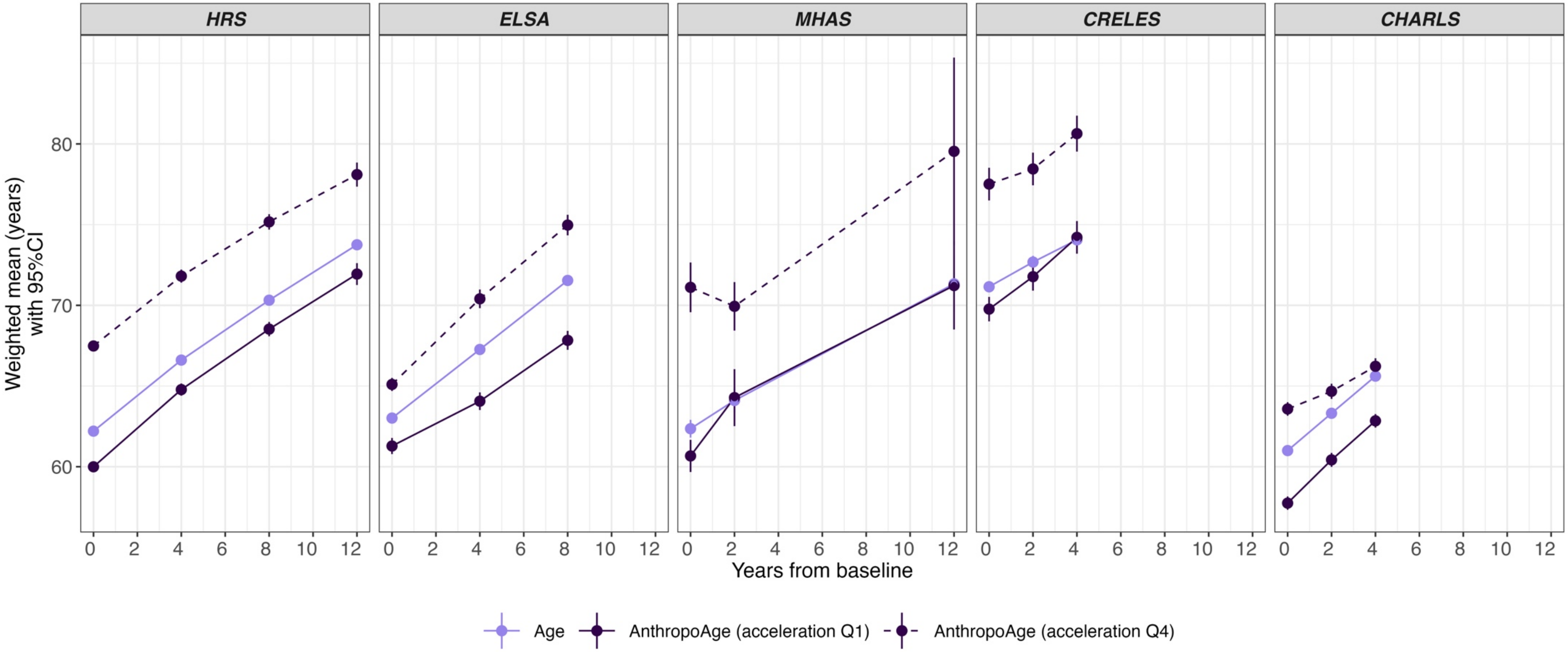

Supplementary Figure 20. Baseline association of AnthropoAgeAccel with the number of comorbidities (**A**) and self-reported health (**B**) in participants aged <70 and ≥70 years old. This association is also reflected in the percentage of participants with accelerated aging (AnthropoAgeAccel >0) for each category (**C-D**).

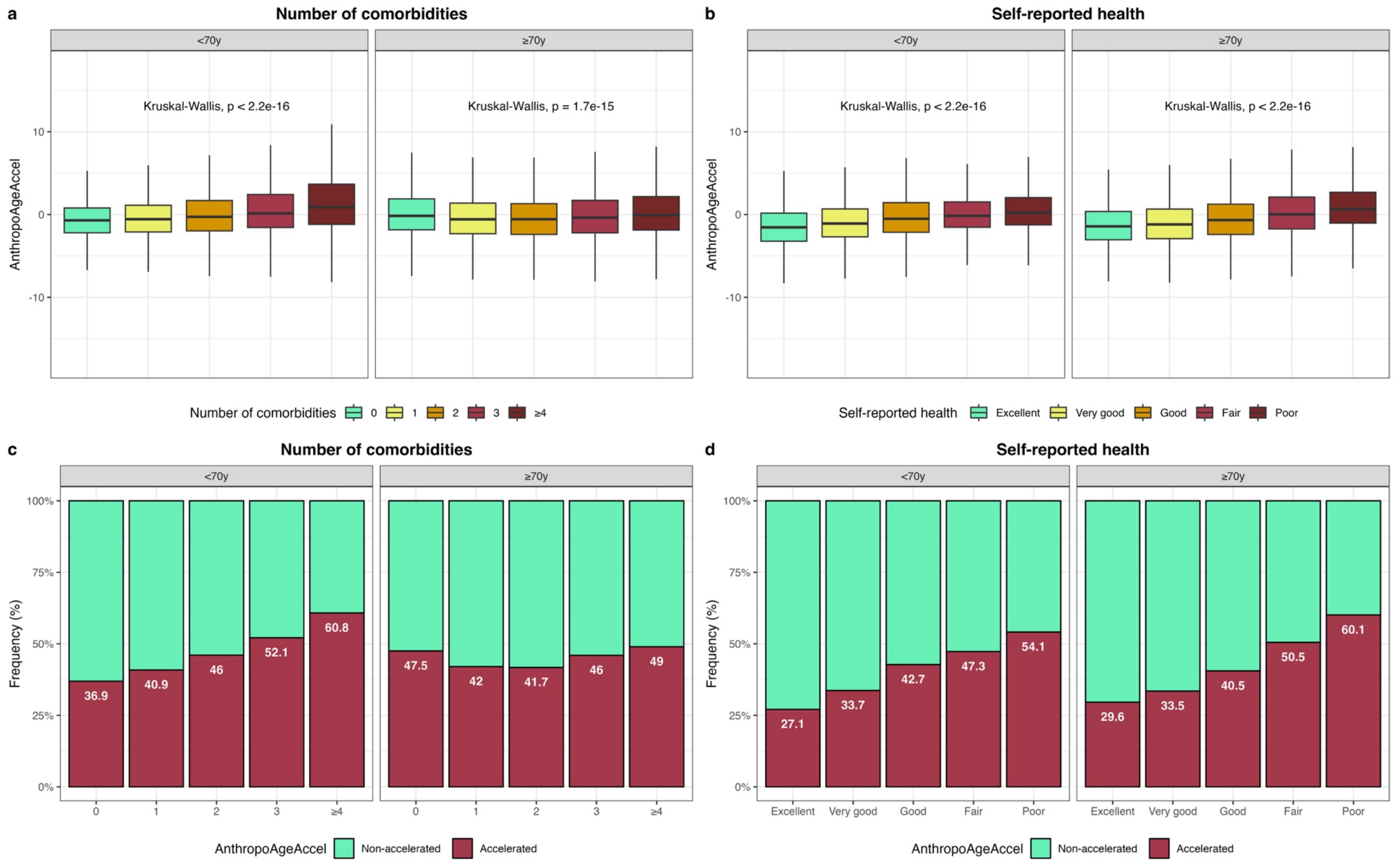

Supplementary Figure 21. Longitudinal associations between AnthropoAgeAccel (weighted mean with 95% confidence interval) and the number of comorbidities (**A**) and self-reported health (**B**) across different follow-up times (years from baseline). Results are stratified by participants who were aged <70 vs ≥70 years old at baseline.

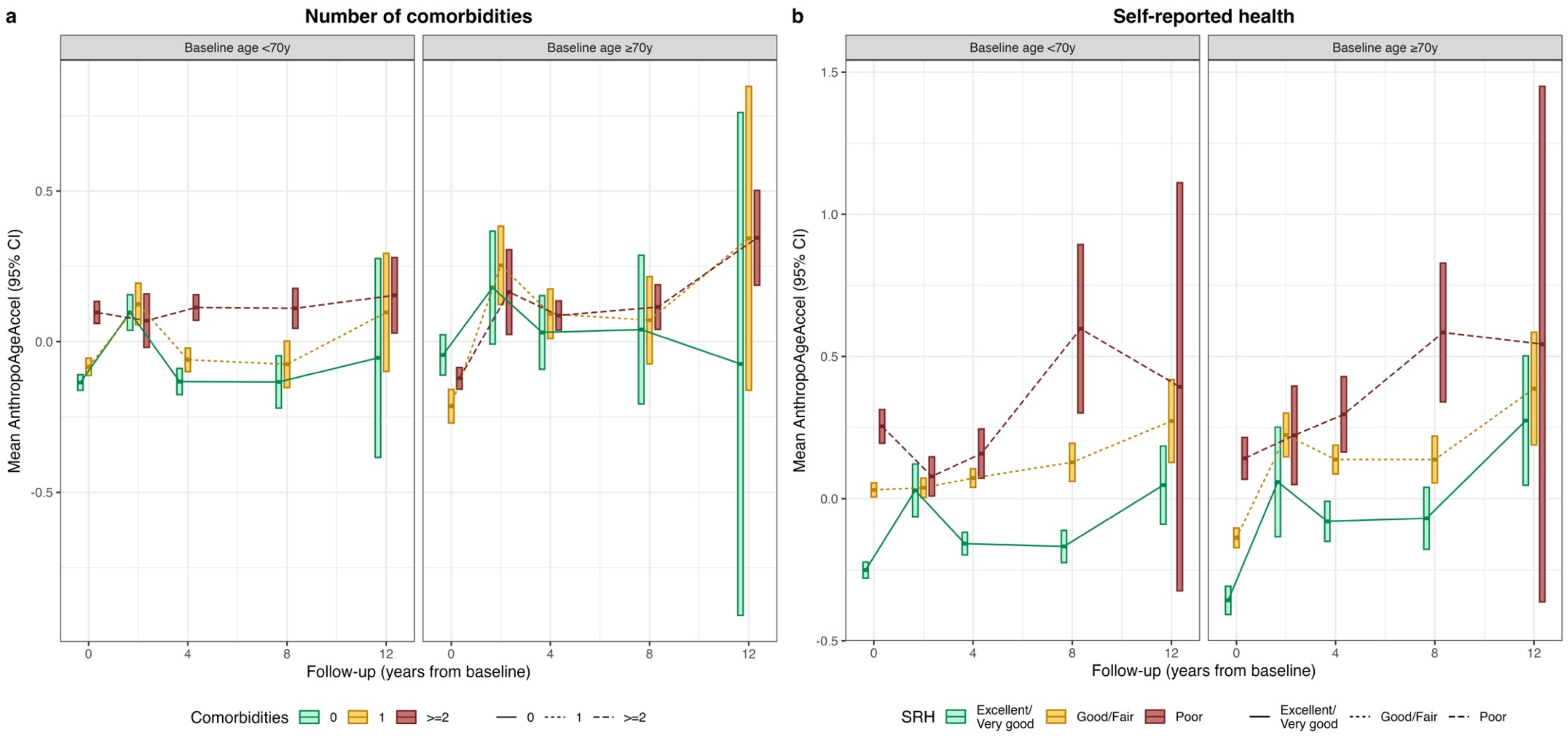

Supplementary Figure 22. Adjusted rate ratio (95% CI) associated to 1-year increases in AnthropoAgeAccel for changes in self-reported health, ADL/IADL deficits and number of comorbidities. Results obtained from GEEs with a Poisson variance function. In this sensitivity analysis, we did not exclude participants who already had the outcome at baseline to improve statistical power.

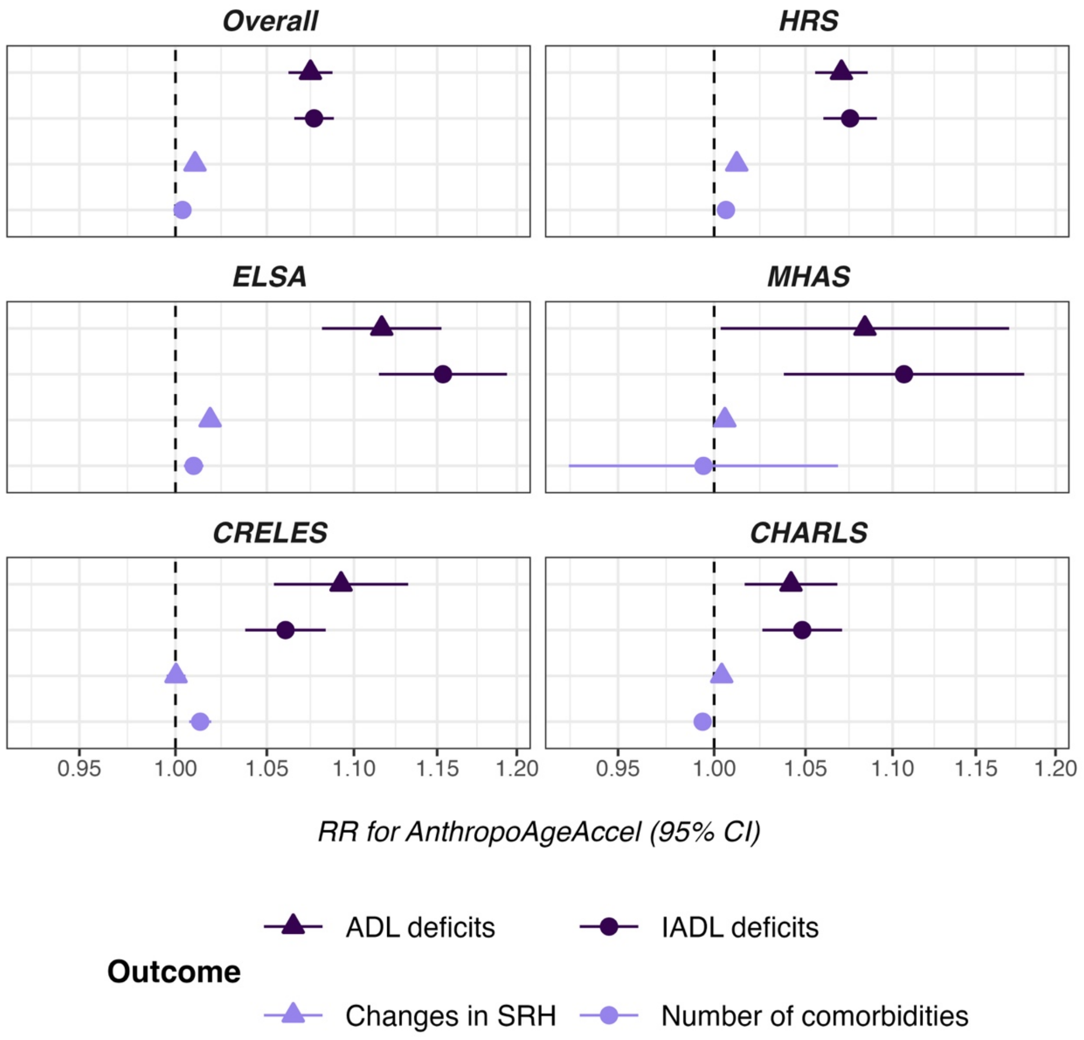

Supplementary Figure 23. Graphical test of proportional hazards using scaled ***Schoenfeld residuals for AnthropoAgeAccel***, along with a smooth curve. Results were obtained from Cox models to predict all-cause mortality stratified by sex and race/ethnicity, and adjusted for CA, education level, smoking, alcohol consumption, and comorbidities. If the proportional hazards assumption holds, the β(t) coefficient vs. follow-up time function should be an approximately horizontal line (p-values for the test of slope=0). We included plots for the overall G2A population (**A**), HRS (**B**), ELSA (**C**), MHAS (**D**), CRELES (**E**), and CHARLS (**F**).

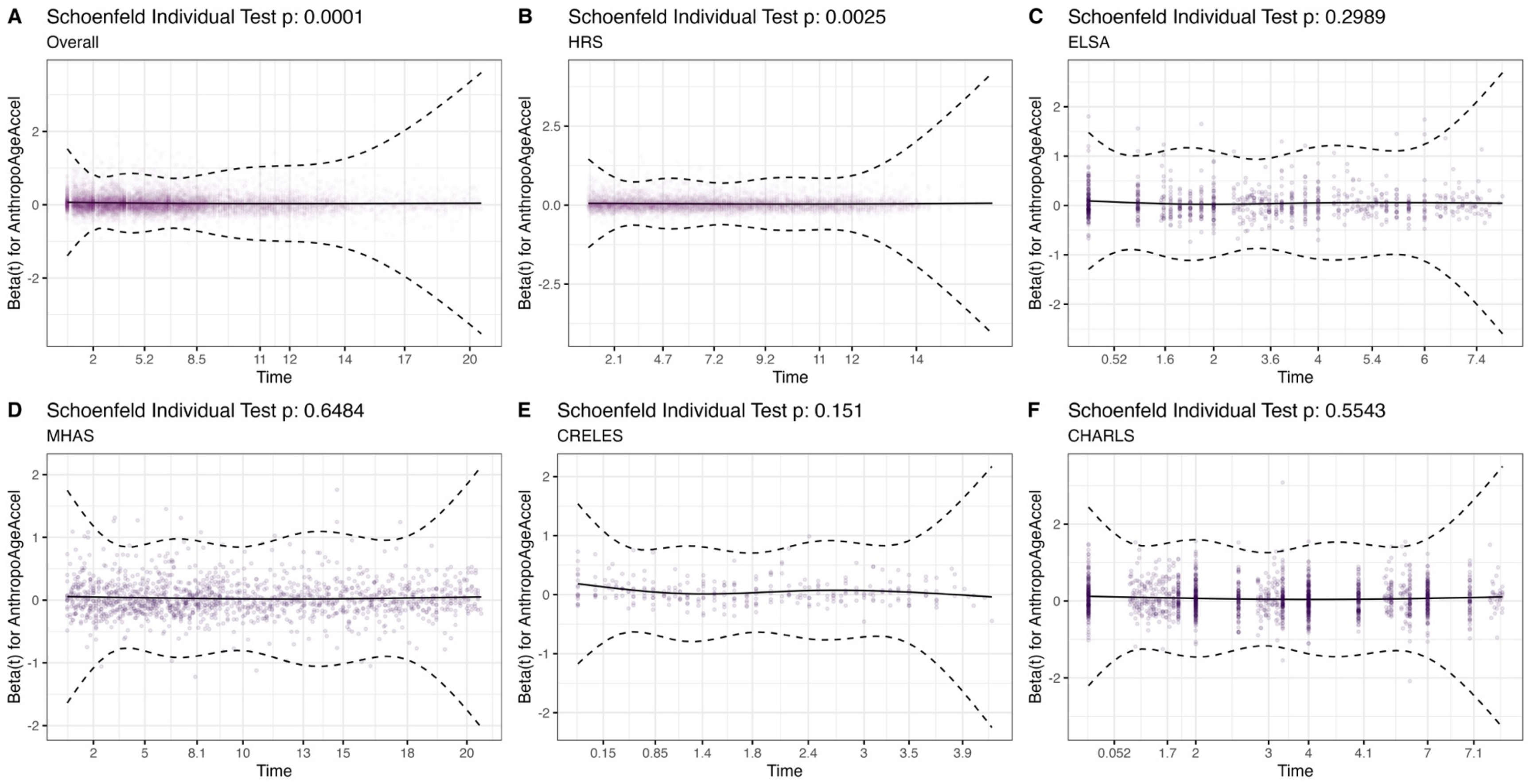

Supplementary Figure 24. Graphical test of proportional hazards using scaled ***Schoenfeld residuals for accelerated aging (AnthropoAgeAccel >0)***, along with a smooth curve. Results were obtained from Cox models to predict all-cause mortality stratified by sex and race/ethnicity, and adjusted for CA, education level, smoking, alcohol consumption, and comorbidities. If the proportional hazards assumption holds, the β(t) coefficient vs. follow-up time function should be an approximately horizontal line (p-values for the test of slope=0). We included plots for the overall G2A population (**A**), HRS (**B**), ELSA (**C**), MHAS (**D**), CRELES (**E**), and CHARLS (**F**).

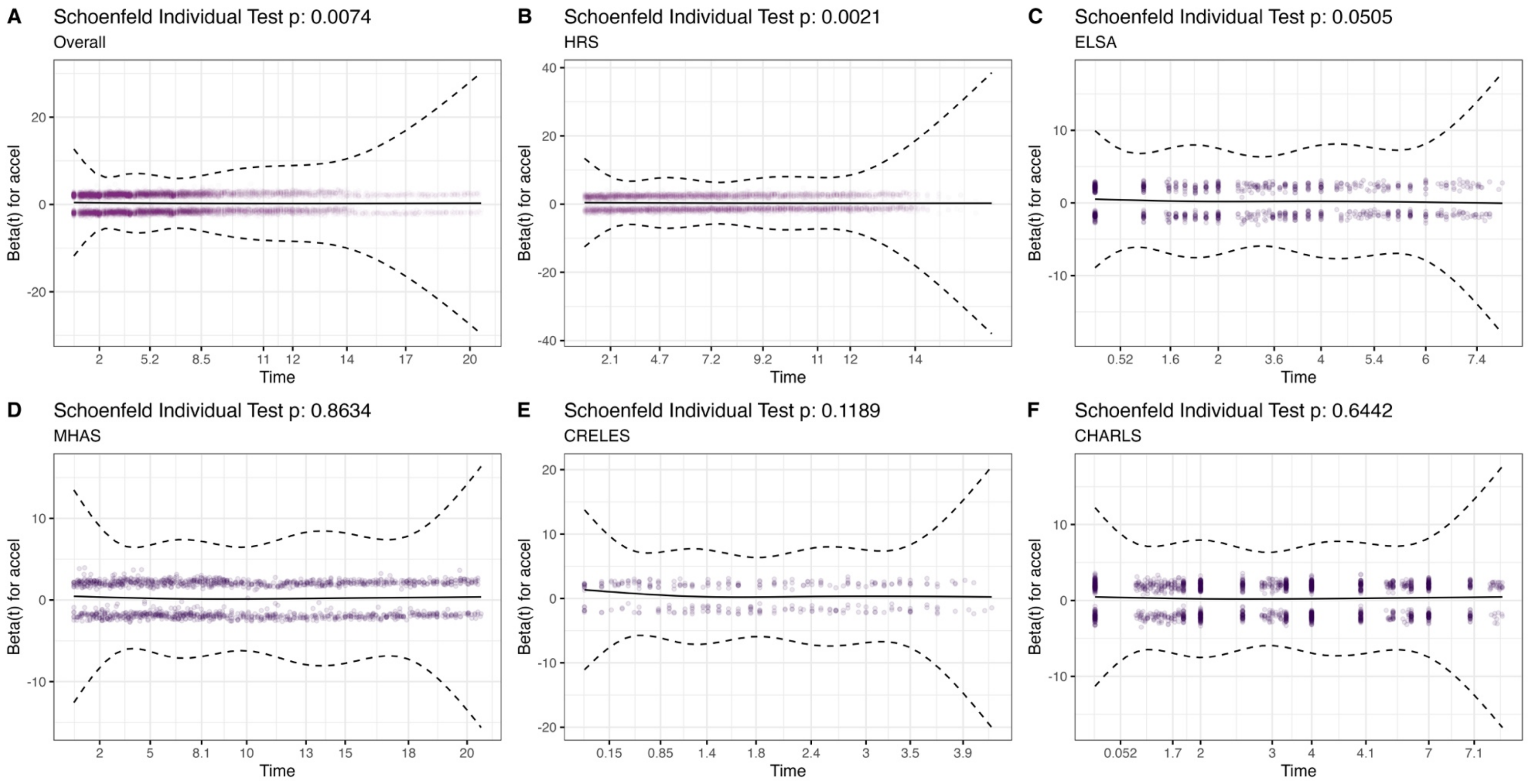

Supplement: Supplementary file 1 — Supplementary Material_V4.0 [file 41514_2025_232_MOESM1_ESM.pdf]
